# YB-1 Mediates TNF-Induced Pro-Survival Signaling by Regulating NF-κB Activation

**DOI:** 10.3390/cancers12082188

**Published:** 2020-08-05

**Authors:** Aneri Shah, Carlos Plaza-Sirvent, Sönke Weinert, Jörn H. Buchbinder, Inna N. Lavrik, Peter R. Mertens, Ingo Schmitz, Jonathan A. Lindquist

**Affiliations:** 1 Clinic of Nephrology and Hypertension, Diabetes and Endocrinology, Otto-von-Guericke University Magdeburg, 39120 Magdeburg, Germany; Aneri.Shah@ovgu.de (A.S.); peter.mertens@med.ovgu.de (P.R.M.); 2 Institute for Molecular and Clinical Immunology, Otto-von-Guericke University Magdeburg, 39120 Magdeburg, Germany; Carlos.PlazaSirvent@rub.de; 3 Systems-Oriented Immunology and Inflammation Research Group, Department of Experimental Immunology, Helmholtz Centre for Infection Research, 38124 Braunschweig, Germany; 4 Department of Molecular Immunology, ZKF2, Ruhr-University Bochum, D-44780 Bochum, Germany; 5 Department of Cardiology and Angiology, Otto-von-Guericke University Magdeburg, 39120 Magdeburg, Germany; Soenke.Weinert@med.ovgu.de; 6 Translational Inflammation Research, Institute of Experimental Internal Medicine, Otto-von-Guericke University Magdeburg, 39120 Magdeburg, Germany; joernbuchbinder@outlook.de (J.H.B.); inna.lavrik@med.ovgu.de (I.N.L.)

**Keywords:** cold shock proteins, TNF, apoptosis

## Abstract

Cell fate decisions regulating survival and death are essential for maintaining tissue homeostasis; dysregulation thereof can lead to tumor development. In some cases, survival and death are triggered by the same receptor, e.g., tumor necrosis factor (TNF)-receptor 1 (TNFR1). We identified a prominent role for the cold shock Y-box binding protein-1 (YB-1) in the TNF-induced activation and nuclear translocation of nuclear factor kappa-light-chain-enhancer of activated B cells (NF-κB) p65. In the absence of YB-1, the expression of TNF receptor-associated factor 2 (TRAF2), a central component of the TNF receptor signaling complex required for NF-κB activation, is significantly reduced. Therefore, we hypothesized that the loss of YB-1 results in a destabilization of TRAF2. Consistent with this hypothesis, we observed that YB-1-deficient cells were more prone to TNF-induced apoptotic cell death. We observed enhanced effector caspase-3 activation and could successfully rescue the cells using the pan-caspase inhibitor zVAD-fmk, but not necrostatin-1. Taken together, our results indicate that YB-1 plays a central role in promoting cell survival through NF-κB activation and identifies a novel mechanism by which enhanced YB-1 expression may contribute to tumor development.

## 1. Introduction

Y-box binding protein-1 (YB-1) belongs to the family of cold shock proteins that are characterized by an evolutionary and structurally conserved cold shock domain [1]. Since YB-1 is a transcriptional and translational regulator, it has pleiotropic functions in cell cycle progression, differentiation, stress responses, DNA damage repair, and inflammation [2,3]. In cultured cells, YB-1 is primarily cytoplasmic; however, upon activation, YB-1 translocates to the nucleus [4,5]. Cell stress occurs in multiple forms, e.g., as lipopolysaccharide produced by bacteria, interferons released in response to viral infection, or as cytokine storms released by immune cells during inflammation. Binding of these factors to their receptors induces kinase activation, which phosphorylates YB-1 [6]. The same pathways also activate nuclear factor kappa-light-chain-enhancer of activated B cells (NF-κB), a well-known transcription factor, which regulates genes involved in immune responses and inflammation.

Functional NF-κB proteins are dimers, possessing Rel homology domain at their N-terminus, hence their classification as NF-κB/Rel proteins. There are five proteins in the NF-κB family: Rel A (p65), RelB, c-Rel, p50/p105, and p52/p100. In the canonical NF-κB pathway, pro-inflammatory cytokines, such as tumor necrosis factor α (TNFα) and interleukin (IL)-1β, activate the IκB (IKK) complex, which is comprised of kinase subunits IKKα, IKKβ, and the regulatory subunit NF-κB essential modifier (NEMO or IKKγ). Once activated, the IKK complex phosphorylates the inhibitor of NF-κB (IκBα), which is subsequently ubiquitinated and degraded by the 26S proteasomal pathway. NF-κB is located in the cytoplasm of resting cells as an inactive complex. The degradation of IκBα results in the release of NF-κB, which then translocates to the nucleus, resulting in the transcriptional activation of its various target genes. These signaling pathways are tightly controlled processes regulated by reversible post-translational modifications, such as protein phosphorylation and ubiquitination.

Life or death decisions within cells are critical for development as well as in the defense against infectious diseases and cancer formation. Apoptosis has long been considered the only form of programmed cell death occurring during development and disease progression. Now, in addition to apoptosis, necroptosis (a programmed form of necrosis) can also be initiated by activation of death receptors [7]. Survival, apoptosis, and necroptosis are all triggered by the same cell surface receptors e.g., tumor necrosis factor receptor 1 (TNFR1). Binding of TNF-α to its receptor TNFR1 leads to the sequential recruitment of various adaptor proteins including TNF-receptor associated death domain protein (TRADD), receptor-interacting serine/threonine-protein kinase 1 (RIPK1), and TNF-receptor associated factor 2 (TRAF2) to the plasma membrane (complex I; see Graphical Abstract). Here, TRAF2, an E3 ubiquitin ligase, plays a central role in the activation of NF-κB [8,9]. TRAF2 recruits the cellular inhibitor of apoptosis protein (cIAP1/2), which ubiquitinates RIPK1. Ubiquitinated RIPK1 serves as a scaffold for the recruitment of the linear ubiquitin assembly complex (LUBAC), composed of Heme-Oxidized IRP2 Ubiquitin Ligase 1 (HOIL-1)/ HOIL-1-Interacting Protein (HOIP)/ SHANK Associated RH Domain Interactor (SHARPIN). LUBAC generates M1-linked linear ubiquitin chains, which recruit the IKK complex (IKK1/IKK2 and NEMO) as well as TGF-beta activated kinase binding protein (TAB) 2 and TAB3. The recruitment of these kinase complexes leads to the activation of NF-κB and mitogen-activated protein kinase (MAPK) signaling [10,11]. In most cells, TNFR1 engagement promotes cell survival via the induction of NF-κB activation [12,13]. Activation of NF-κB mediates pro-survival signaling by means of cIAP1/2 and the cellular FLICE-like inhibitory protein (cFLIP) [14,15]. In the absence of NF-κB activation, TNF stimulation triggers caspase-8-mediated apoptosis by a defined death effector complex II, composed of TRADD, Fas-associated death domain (FADD), RIPK1, and procaspase-8 [16,17].

Previously, YB-1 was identified as an essential factor for NF-κB activation downstream of the IL-1β receptor [18,19]. Therefore, we speculated that YB-1 might also play a role in TNF receptor-mediated NF-κB activation. Our study reveals a crucial role of YB-1 in TNFR1-mediated cell fate decisions. Genetic deletion of Ybx1 abrogates activation of NF-κB, thereby promoting programmed cell death via the activation of complex II. However, complex II can lead to either apoptosis or necroptosis. Activation of necroptosis is initiated by the deubiquitination of RIPK1 by cylindromatosis (CYLD); this displaces RIPK1 from complex I, allowing it to then form complex II (together with TRADD, FADD, and caspase-8). Active caspase-8 cleaves RIPK1 and RIPK3; however, inhibition of caspase-8 facilitates the interaction of RIPK1 and RIPK3 (i.e., the necrosome) [20]. RIPK1 activates RIPK3, which in turn phosphorylates the pseudokinase mixed-lineage domain-like protein (MLKL) [21,22,23]. Phosphorylation of MLKL facilitates its oligomerization and insertion into lipid membranes, thereby inducing pore formation that results in necrotic cell death [24]. The application of inhibitors, e.g., pan-caspase inhibitor zVAD-fmk (ZVAD) and necrostatin-1 (Nec-1), allows one to distinguish whether cells are dying via apoptosis or necroptosis [25].

## 2. Results

### 2.1. YB-1 Was Responsible for TNF-Induced NF-κB Activation

**Figure 1:**
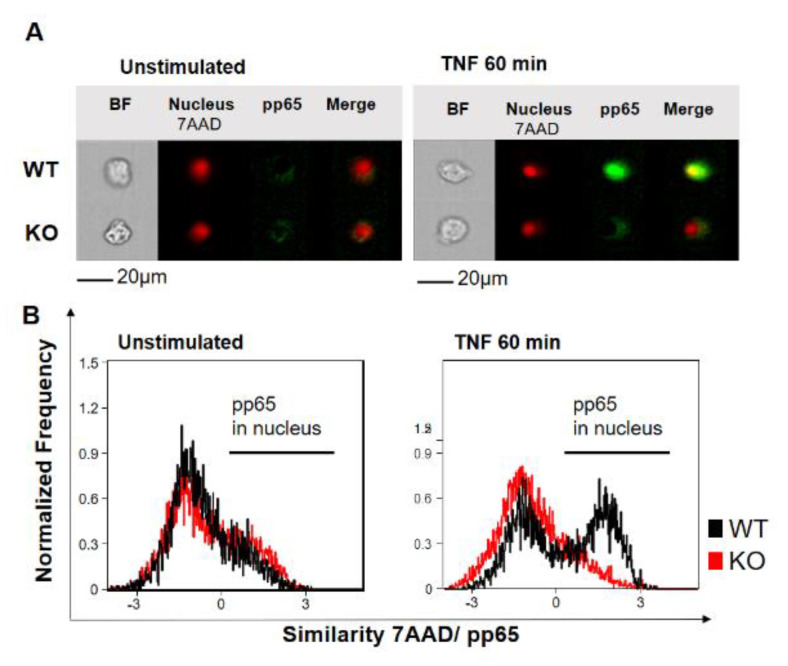
Y-box binding protein-1 (YB-1) is essential for tumor necrosis factor (TNF)-induced NF-κB nuclear translocation in bone marrow-derived macrophages. (**A**) Imaging flow cytometry showing one representative cell. The nucleus stained positive for 7-Amino-actinomycin D (7AAD) (red). Staining for NF-κB phospho-p65 activation (pp65) is shown with and without stimulation (green). Colocalization (yellow) indicates nuclear translocation. (**B**) Normalized frequency of wild type (WT) (black) and YB-1 knockout (KO) (Ybx1^ΔLysM^) cells (red) showing nuclear translocation of activated NF-κB p65 with and without TNF stimulation (20 ng/mL).

**Figure 2:**
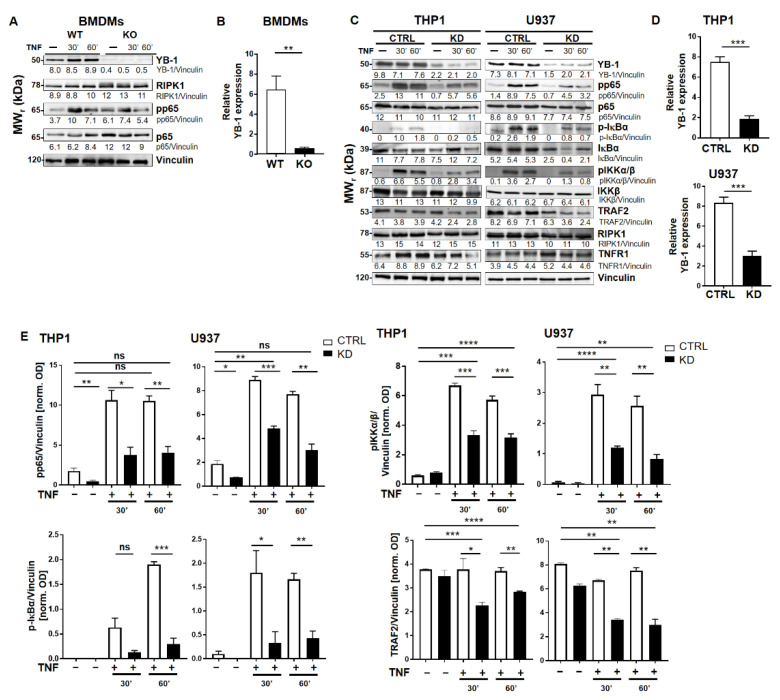
YB-1 was required for TNF-induced NF-κB activation. (**A**) Bone marrow-derived macrophages (BMDM, WT, and YB-1 KO) were stimulated with TNF (20 ng/mL) for the indication of time periods to activate the NF-κB signaling pathway. Protein expression was analyzed by immunoblotting using the indicated antibodies. Vinculin was used as the loading control. The relative band intensities are indicated. (**B**) Relative YB-1 expression is shown for three independent experiments. WT (white) and KO (black bars). (**C**) Control and YB-1 knockdown (KD) cells (THP-1 and U937) were stimulated with TNF (20 ng/mL) to activate tumor necrosis factor receptor 1 (TNFR1). Proteins were analyzed by immunoblotting for expression of the indicated proteins. Vinculin was used as loading control. Control (CTRL; white) and knockdown (KD; black bars). (**D**) Relative YB-1 expression in KD cells is shown for three independent experiments. (**E**) Relative pp65, phospho- inhibitor of NF-κB alpha (p-IκBα), phospho-IkB kinase α/β (pIKKα/β), and TNF receptor-associated factor 2 (TRAF2) expression in WT and KD THP-1 and U937 cells are shown for three independent experiments. Control (CTRL; white) and knockdown (KD; black bars). Error bars specify the standard error of the mean (SEM). Statistical significance was calculated using an unpaired *t*-test, *n* = 3. Data represent the mean ± SEM. * *p* < 0.05, ** *p* < 0.01, *** *p* ≤ 0.001, **** *p* ≤ 0.0001.

To investigate the role of YB-1 within the TNFR signaling pathway, we isolated primary bone marrow-derived macrophages (BMDMs) from either wild type (WT) or Ybx1^ΔLysM^ knockout (KO) mice [26]. After 10 days in culture with granulocyte/macrophage-colony stimulating factor (GM-CSF), macrophages were harvested and stimulated with TNF (20 ng/mL). The nuclear translocation of NF-κB phospho-p65 (pp65) was observed using imaging flow cytometry (Figure 1A,B). Wild type cells showed the expected translocation of NF-κB into the nucleus after 60 min of TNF stimulation, whereas in KO cells, no NF-κB translocation was detected (Figure 1B). The data obtained in BMDMs were confirmed in human knockdown cells (Figure S1). Analysis of the BMDMs by Western blot showed that the loss of YB-1 did not influence p65 expression, only its activation (Figure 2A,B). To validate this observation in human cells, we suppressed YB-1 expression in two monocytic cell lines using specific lentiviral short-hairpin RNA (shRNA), as previously published [27]. Western blot analysis showed that in the absence of YB-1, both p65 and TNF receptor 1 expression remained unchanged in both cell types (Figure 2C). Quantification showed that a ≈75% reduction in YB-1 expression is achieved compared to control cells (Figure 2D). Following TNF stimulation, knockdown cells showed a compromised phosphorylation of the NF-κB inhibitor IκBα. As a result, the TNF-induced phosphorylation and activation of NF-κB p65 was significantly reduced in knockdown (KD) cells compared to control. The protein kinase upstream of IκBα, i.e., IKKα/β, also showed compromised phosphorylation. Thus, we hypothesized that the recruitment of components to the receptor complex may have been involved. Therefore, we investigated the TRAF2 protein (Figure 2C), since it is important for the recruitment of NF-κB to the TNFR signaling complex [11,12]. Following TNF stimulation of YB-1 KD cells, we observed a significant reduction of TRAF2 protein (Figure 2E). This observation supported our hypothesis and pointed towards a defect in TNF receptor complex formation (see Graphical Abstract).

### 2.2. In the Absence of YB-1, TNFR Activation Induced Apoptosis

**Figure 3:**
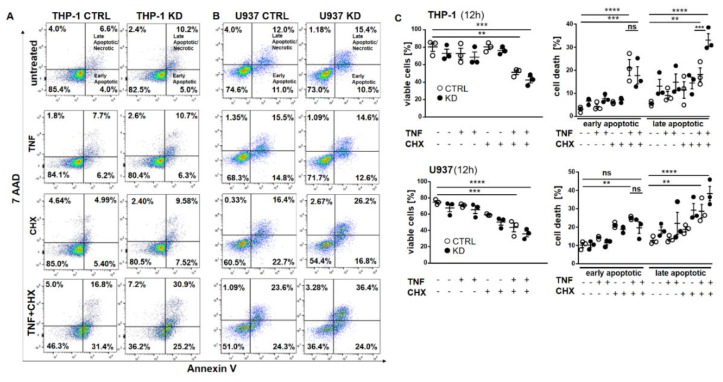
YB-1 was protective against cell death in vitro. (**A**,**B**) Representative flow cytometry dot plot analyses of THP-1 (**A**) and U937 cells (**B**). Cells were treated with or without TNF (20 ng/mL) and cycloheximide (10 ng/mL) alone or together overnight. Cells were harvested and stained for annexin V and 7AAD. (**C**) Graphs representing flow cytometry analysis of live and dead cells in THP-1 and U937 cells. Statistical significance was calculated by two-way ANOVA with Bonferroni post hoc test, *n* = 3. Data represent the mean ± SEM. ** *p* < 0.01, *** *p* ≤ 0.001, **** *p* ≤ 0.0001.

**Figure 4:**
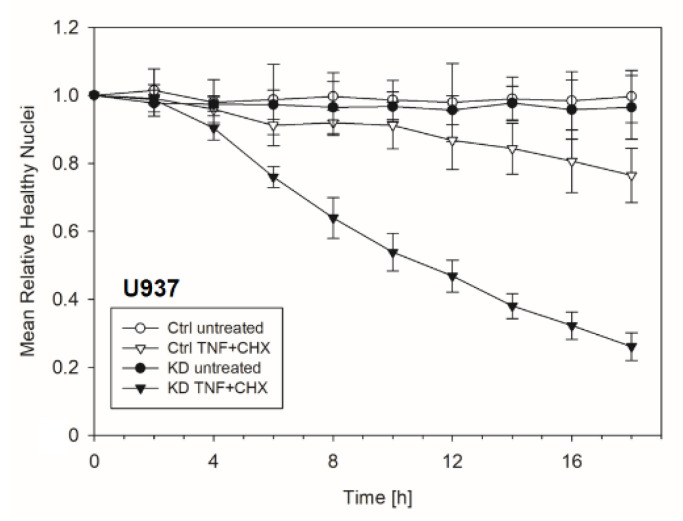
Enhanced apoptosis upon TNF + cycloheximide (CHX) stimulation of YB-1-deficient cells. The graph showing the normalized number of healthy nuclei per field of view over indicated period of time. The total number of healthy nuclei decreased after 4 h of TNF + CHX treatment.

**Figure 5:**
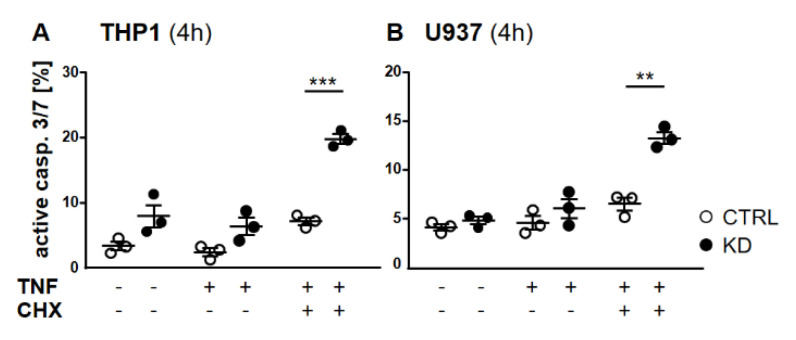
Depletion of YB-1 led to effector caspase activation. (**A**,**B**) Flow cytometry analyses of caspase-3/7 activation in THP-1 (**A**) and U937 cells (**B**). Cells were treated with or without TNF (20 ng/mL) alone or together with cycloheximide (10 ng/mL) for 4 h. Cells were harvested and stained for caspase-3/7 activation. Error bars specify the standard error mean (SEM) of at least three experiments. Statistical significance was calculated by unpaired *t*-test, *n* = 3. Data represent the mean ± SEM. ** *p* < 0.01, *** *p* ≤ 0.001.

Frequently, NF-κB signals for survival, and therefore inactivation of NF-κB leads to enhanced apoptotic cell death [13]. Taking into consideration that TNF-induced NF-κB activation is defective in YB-1-deficient cells, we hypothesized that the loss of YB-1 might sensitize cells to TNFR-induced apoptosis. To investigate this, the monocytic cell lines (THP-1 and U937) were left untreated, treated overnight with either TNF or cycloheximide (CHX) alone, or treated together. CHX inhibits protein synthesis, in particular the pro-survival gene product c-FLIP [14]. Cells were then analyzed by flow cytometry using annexin V and 7-Amino-actinomycin D (7AAD) staining to distinguish apoptotic and/or necrotic cells (Figure 3A,B). Compared to WT, untreated YB-1-deficient cells appeared to show an increased basal rate of cell death. The population of early apoptotic cells that were annexin-positive and 7AAD-negative remained unaltered. TNF or CHX alone do not induce cell death in either the presence or absence of YB-1. We observed a ≈50% increase in late apoptotic/necrotic cells upon combined treatment. The percentage of viable cells was reduced in the absence of YB-1 (Figure 3C). The percentage of double positive cells (late apoptotic) was higher in KD compared to control cells (Figure 3C). Visual observation of the cells supported the flow cytometry results, suggesting that YB-1-deficient cells are more susceptible to cell death. To confirm this, we performed time lapse microscopy (Figure 4, Figure S2, Videos S1–S4), which showed that TNF+CHX-treated YB-1-deficient cells died faster than control cells. Since we observed enhanced cell death, we next investigated whether we could also detect enhanced effector caspase-3/7 activation (Figure 5A,B). Effector caspase-3/7 are activated by initiator caspases, such as caspase-8, and cleave a number of different target proteins that play an important role in mediating apoptotic cell death [28,29]. Activation of caspase-3/7 was enhanced in the YB-1 KD cells (Figure 5A,B). Taken together, we observed a dramatic upregulation of cell death upon sensitization with CHX, which was augmented by the loss of YB-1.

### 2.3. YB-1-Deficient Cells Died Largely Via Apoptosis

**Figure 6:**
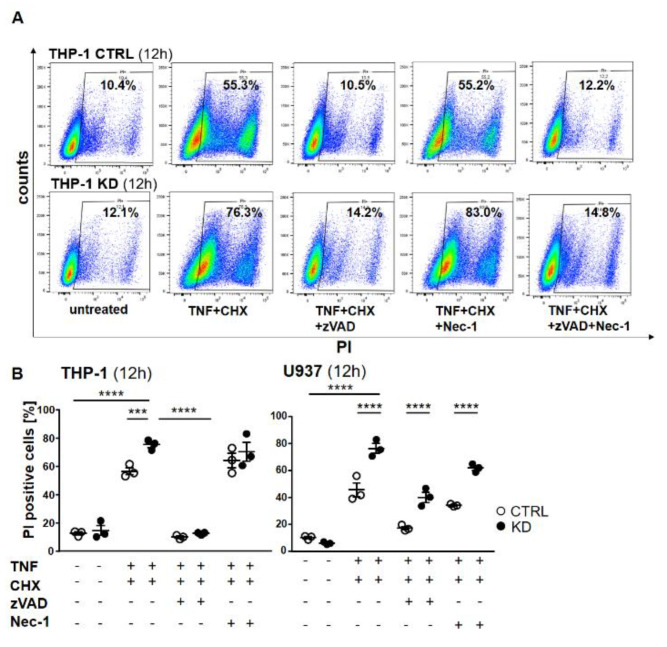
Cell death was rescued using the pan-caspase inhibitor zVAD-fmk (ZVAD). (**A**) THP-1 cells were pretreated with zVAD and/or necrostatin-1 (Nec-1) for 1 h and treated with TNF and CHX overnight. The cells were harvested, stained with propidium iodide (PI), and analyzed by flow cytometry. The percentage of dead cells following treatment is indicated. (**B**) Graphs representing flow cytometry analysis of THP-1 (left panel) and U937 (right panel). Error bars specify the standard error mean (SEM) of at least three experiments. Statistical significance was calculated by two-way ANOVA with Bonferroni post hoc test, *n* = 3. Data represent the mean ± SEM. *** *p* ≤ 0.001, **** *p* ≤ 0.0001.

To distinguish apoptosis from necroptosis, we pre-treated the cells with either the pan-caspase inhibitor Z-Val-Ala-Asp-fluoromethyl ketone (Z-VAD) or the RIPK1 inhibitor necrostatin 1 (Nec-1) or their combination, and then stimulated the cells with TNF + CHX to induce cell death. As shown in Figure 3, TNF + CHX treatment dramatically induced cell death. Pre-treatment with zVAD completely rescued both control and KD cells from dying (Figure 6A), whereas cells treated with Nec-1 were still prone to cell death. Western blotting confirmed the activation of caspase-8 and -3, as well as the efficacy of the inhibitor (Figure S3 and Figure S4), thus indicating that in the absence of YB-1, THP-1 cells died via apoptosis and not necroptosis (Figure 6A). Dot plots show a significant difference upon TNF + CHX treatment in control and KD cells (Figure 6B). Surprisingly, the effect of the inhibitors was somewhat different with U937 cells. Here, zVAD offered better protection against TNF + CHX-induced death in terms of controlling cells when compared with Nec-1. Although the pattern was similar, YB-1-deficient cells continued to show an enhanced propensity for cell death, suggesting that in the absence of YB-1 an alternative cell death pathway was activated.

## 3. Discussion

The process of ubiquitination plays a central role in determining the cellular response to TNFR1 signaling. Upon ligand binding, RIPK1 is rapidly recruited and actively ubiquitinated within complex I. Ubiquitinated RIPK1 then serves as a scaffold for the recruitment of downstream components such as LUBAC. Linear ubiquitination of distinct components of complex I (RIPK1, TRADD, and TNFR1) by LUBAC reinforces complex I, allowing efficient recruitment of the IKK complex leading to the activation of NF-κB [30]. It is known that the ubiquitination and deubiquitination events on RIPK1 are critical for determining cell fate [31]. In the absence of ubiquitination, RIPK1 dissociates from complex I and recruits FADD and pro-caspase-8 to form complex II. Caspase-8, an initiator caspase, auto-activates through oligomerization at complex II and subsequently activates downstream caspases, e.g., the effector caspases-3 and -7. As the expression of RIPK1 is equivalent upon TNF stimulation in YB-1 KO/KD cells (Figure 2), we suspect that a dysregulated RIPK1 ubiquitination could explain the shift to complex II formation. However, whether this is due to changes in E3 ligase activity (cIAP and LUBAC) or deubiquitinating enzymes (CYLD, tumor necrosis factor alpha-induced protein 3 (Tnfip3 or A20), and OTU deubiquitinase with linear linkage specificity (OTULIN)) is unclear. YB-1 had previously been reported as an integral component of the TNFR signaling pathway, however, its position within the network is unclear. Within YB-1, five sites of Ser phosphorylation were identified upon TNF stimulation, including Ser165 and Ser176 [32].

Prabhu et al. showed that phosphorylation at Ser165 and Ser176 within YB-1 is required for NF-κB activation via the IL-1βR [18,19]. However, how phosphorylation at these sites contributes to NF-κB activation is unclear. Importantly, phosphorylation at these sites promotes tumor formation by differentially regulating the expression of different subgroups of NF-κB target genes. In addition to phosphorylation, YB-1 was also reported to be ubiquitinated and functionally interact with other proteins upon TNF stimulation [32]. Therefore, we suspect that an imbalance within the ubiquitination status caused by the loss of YB-1 led to the apoptotic phenotype observed.

Multiple sites of ubiquitination have been identified within YB-1 [33]. Interestingly, three of these sites were also identified to be ubiquitinated upon TNF stimulation [32]. The Homologous to E6AP C-terminus (HECT) domain E3 ligase HECT domain and ankyrin repeat containing E3 ubiquitin protein ligase 1 (HACE1) is a central gatekeeper for TNFR1-induced cell death. HACE1 is required for the ubiquitination of TRAF2 and thereby the formation of the apoptotic complex [34]. We showed that YB-1 plays an important role in regulating the expression and/or stability of TRAF2 (Figure 2). However, the mechanism by which YB-1 influences TRAF2 expression is unclear. HACE1 has been reported to interact with YB-1, however, this involves the polyubiquitination of the YB-1 protein through K27-linked ubiquitin chains leading to protein secretion [35]. In addition to HACE1, YB-1 also interacts with the deubiquitinase otubain-1 (OTUB1) [36]. OTUB1 is responsible for removing K48-linked ubiquitin chains from YB-1 and thereby stabilizes YB-1 expression. Since OTUB1 also modulates the stability of cIAP1, it is a likely candidate that would explain the observed phenotype. Thus, the YB-1-dependency for NF-κB activation clearly points to an important role for YB-1 in recruiting other components to complex I in the TNFR signaling cascade (see Graphical Abstract). If this is true, then why have other studies not identified YB-1 as a component of the TNF receptor signaling complex? The answer is that they have, but due to YB-1′s plethora of activities, it was excluded from the results [37].

Identifying the molecular mechanism of YB-1-mediated NF-κB activation may identify therapeutic targets for interventions in cancer. We envision that targeting YB-1 will sensitize cells to TNF-induced apoptosis. Here, however, we envision the requirement for a second hit, as our data show that the loss of YB-1 alone is not sufficient for TNF-induced apoptosis.

## 4. Materials and Methods

### 4.1. Cell Culture and Stimulation

The cell lines THP-1 (TIB-202) and U937 (CRL-1593) were obtained from the American Type Culture Collection (ATCC, Manassas, VA). THP-1 cells were cultured in Roswell Park Memorial Institute (RPMI) medium and supplemented with 10% fetal calf serum (FCS), 1% penicillin/streptomycin, and 50 µM β-mercaptoethanol, and U937 cells were cultured in RPMI medium and supplemented with 10% fetal calf serum and 1% penicillin under humidified conditions at 37 °C and 5% CO_2_. Cells were stimulated with recombinant TNF-α (20 ng/mL; R&D Systems) for the indicated time points and were subsequently washed with ice-cold phosphate-buffered saline. Cells were lysed with Radio-Immunoprecipitation Assay (RIPA) lysis buffer (50 mM Tris Base, 150 mM NaCl, 1 mM ethylenediaminetetraacetic acid (EDTA), 1% Nonidet (NP-40), 0.25% sodium deoxycholate) supplemented with complete mini protease cocktail inhibitor cocktail (Roche, Mannheim, Germany) Phospho-stop (Roche). Lysates were cleared by centrifugation at 14,000× *g*. Protein concentrations were estimated using Lowry assay.

### 4.2. Bone Marrow-Derived Macrophages

Bone marrow-derived macrophages (BMDMs) were generated from wild type or Ybx1^ΔLysM^-deficient mice by flushing femur and tibia with sterile Dulbecco’s phosphate-buffered saline (DPBS). Erythrocytes were lysed under hypotonic conditions and were cells seeded with 2 × 10^6^ cells/mL in RPMI growth media supplemented with 10% FCS, 1% penicillin/streptomycin, and murine macrophage colony-stimulating factor (10 ng/mL; M-CSF, 315-02, Peprotech). Cells were cultivated under humidified conditions at 37 °C and 5% CO_2_. Cells were fed every 2 days until fully differentiated (after 7 days) [38].

Animals were maintained according to the FELASA guidelines (Federation of European Laboratory Animal Science Association) in a 12 h/12 h light/dark cycle at 22 °C in the Central Animal Facility of the Otto-von-Guericke University Magdeburg under specific pathogen-free (SPF) conditions using individual ventilated cages (IVC, Techniplast, Buguggiate, Italy) with free access to food and water. All experimental procedures were conducted in accordance with the German National Guidelines for the Use of Experimental Animals (Animal Protection Act) and approved by the State of Saxony-Anhalt (AZ UniMD 42502-2-1401).

### 4.3. Lentiviral Transduction of YB-1

The plasmids pLKO and pLKO-YB-1 shRNA were from Sigma-Aldrich. For knockdown, YB-1 shRNA and scrambled shRNA were used. (shRNA:CCGGCCAGTTCAAGGCAGAAATATCTCGAGATATTTACTGCCTTGAACTGG-TTTTTG). Human embryonic kidney cells (HEK293T; 8 × 10^5^) were seeded in 6-well plates the day before viral transduction and co-transfected with 2 µg YB-1 construct plasmid, 1 µg psPAX2, and 1 µg pVSV-G with calcium phosphate precipitates. Chloroquine (25 µM) was added to the cells immediately before transfection. After 1 day, the medium was exchanged with Dulbecco’s Modified Eagle Medium (DMEM) supplemented with 10% FCS, 1% penicillin, 10 mM hydroxyethyl piperazineethanesulfonic acid (HEPES) buffer (Gibco, Dublin, Ireland), and 1 mM sodium butyrate (Sigma, Darmstadt, Germany). Virus-containing supernatants were harvested and filtered with 0.45 µm filter, and 4 µg/mL polybrene was added, and the target cells (THP-1 and U937) were infected. After 6 h of transduction, an equal volume of fresh medium was added, and the medium was exchanged with fresh medium the following day. Stably transduced cell lines were selected using puromycin (1.5 µg/mL) for 7–14 days. Cells were harvested 3 days after transduction for Western blot analysis.

### 4.4. SDS-PAGE and Western Blotting

Proteins were resolved using 10% sodium dodecyl sulfate polyacrylamide gel electrophoresis and blotted onto nitrocellulose membranes. The membranes were blocked with 5% dry milk in Tris-buffered saline-Tween. Primary antibodies were incubated overnight at 4 °C and diluted according to the manufacturer’s instructions. The following antibodies were used: anti-NF-κB p65 (6956, Cell Signaling, Frankfurt am Main, Germany), anti-phospho-NF-κB p65 (pp65; 3033, Cell Signaling), anti-YB-1 (Eurogentec, Liège, Belgium), anti-TRAF2 (558,890, BD Biosciences), anti-RIP (610,459, BD Biosciences, Heidelberg, Germany), IκBα (9242, Cell Signaling), anti-p-IκBα (9246, Cell Signaling), anti-TNFR1 (8436, Santa Cruz, CA, USA), anti-vinculin (59,803, Santa Cruz), anti-mono- and polyubiquitinated protein (clone FK2, Enzo), anti-cleaved caspase-3 (9661, Cell Signaling), anti-caspase-8 (4790, Cell Signaling), anti-cleaved caspase-8 (9496, Cell Signaling), and anti-pIKKα/β (2697, Cell Signaling). Secondary antibodies coupled to horseradish peroxidase (Southern Biotech, Birmingham, AL, USA) were used for immuno-detection. The detection was performed using Pierce ECL Western blotting substrate (32106, Thermo Fischer Scientific, Waltham, MA, USA).

### 4.5. Imaging Flow Cytometry

BMDMs and THP-1 cells were cultured and stimulated as described above. Cells were centrifuged at 500× *g* at 4 °C for 5 min and washed twice with 0.5 mL incubation buffer [5 g/L bovine serum albumin (BSA) in phosphate buffer saline (PBS)]. After washing, the pellet was suspended in 50 μL incubation buffer and stained with anti-NF-κB phospho-S529 p65 phycoerythrin (PE) antibody (clone REA348, Miltenyi). After 1 h incubation at RT in the dark, the cells were washed with 0.5 mL incubation buffer again and suspended in PBS. 7AAD (BioLegend) was added at least 5 min before measuring. The cells were measured with an Amnis FlowSight (Merck Millipore). 7AAD was measured in channel 5 (642–745 nm) and PE in channel 3 (560–595 nm). The nuclear translocation of NF-κB phospho-p65 was determined by the overlap between phospho-p65 and 7AAD.

### 4.6. Cell Death Assays

To quantify apoptosis, we seeded THP-1 and U937 cells at 1 × 10^6^ cells per well in 24-well plates. Cells were pretreated with pan caspase inhibitor z-VAD (1:1000) and RIPK1 inhibitor Nec-1 (50 µM, Calbiochem, USA) or BV6 (5 µM, Adooq Bioscience, Canada) for 1 h and then stimulated with TNF (20 ng/mL) and cycloheximide (10 µg/mL) for the indicated time points. To detect dead cells, the samples were stained with propidium iodide (10 µg/mL) and measured by flow cytometry. To assess apoptosis, we stained untreated and treated cells (as mentioned above) with annexin V (BD Biosciences) and 7AAD (Enzo Life Sciences, Germany) to discriminate between apoptotic and necrotic cells. The cleavage of caspase-3/7 was determined using Caspase-3/7 Green Reagent (Invitrogen). After stimulation (as mentioned above), the cells were incubated with diluted reagent (1:5000) for 10 min and measured with flow cytometry.

### 4.7. Time-Lapse Microscopy

For each stimulation condition, we separated a Fluorodish into two small and one big volume using 2-well silicone inserts. The two small compartments were coated with a Retronectin solution, overnight. The compartments were washed with PBS, and 28,000 cells of each control and knockdown U937 cells were seeded into small compartments using RPMI medium, supplemented with 10% fetal calf serum, 1% penicillin and Hoechst 33,342 (5 µg/mL), and propidium iodide (1 µg/mL). After 2 h of adhesion, non-adherent cells were washed away, and the surrounding compartment was filled with same medium along with and without TNF (20 ng/mL) and cycloheximide (10 µg).

Subsequently, the silicone insert was removed thereby both control and knockdown cells were exposed to the surrounding cell culture medium. Within each of the four fields, fields of view were defined. Repetitive images of the Hoechst 33,342 and propidium iodide (PI) staining were acquired along with a phase contrast for all of the 20 fields of view for every 150 s for 18 h.

### 4.8. Image Segmentation

To analyze cell death in U937 cells, we exported the 26 k single channel images as unaltered 8-bit tiff files. The images were analyzed in cell profiler software using pipeline consisting of an Otsu two class threshold, with a size exclusion for the diameter of 10–40 pixels (6.5–26 µm) for the healthy nuclei, an Otsu two class threshold for PI-positive objects as well as PI-positive nuclei fragments per image. The number of healthy nuclei was normalized to nuclei number in the first frame.

## 5. Conclusions

In conclusion, we identified YB-1 as a key component modulating TNFR complex I formation that plays an essential role in initiating TNF-induced NF-κB activation. Whether YB-1 is directly involved in complex I or merely modifies complex I components, similar to OTULIN [39], has yet to be determined. In the absence of YB-1, sensitized cells showed an enhanced rate of cell death. Our ability to “rescue” cells using the pan-caspase inhibitor zVAD, but not necrostatin-1, clearly identified the mechanism of cell death as apoptosis, which indicated a switch from complex I to complex IIa. Unraveling the molecular mechanism underlying this phenotype is essential in order to determine whether interventions targeting YB-1 may be of clinical benefit.

## Supplementary Materials

The following are available online at https://www.mdpi.com/2072-6694/12/8/2188/s1, Figure S1: YB-1 is essential for TNF-induced NF-κB nuclear translocation in a human monocytic cell line (THP-1). Figure S2: Representative single images each selected from fields of view from the time lapse microscopy, Figure S3: YB-1 knockdown enhanced caspase activation, Figure S4: YB-1 knockdown enhanced caspase activation; whole blots are provided, Supplementary movies from the time lapse microscopy are also provided. Video S1: CTRL. Video S2: KD. Video S3: CTRL TNF + CHX. Video S4: KD TNF + CHX.
