# Supplementary material for: YB-1 Mediates TNF-Induced Pro-Survival Signaling by Regulating NF-κB Activation
Source: Cancers (Basel). 2020 Aug 5;12(8):2188. doi: 10.3390/cancers12082188 (PMC7464034; doi:10.3390/cancers12082188)
Supplement: Supplementary file 1 [file cancers-12-02188-s001.zip › cancers-860606 supplementary resubmitted.docx]

**Supplementary Materials:**

YB-1 Mediates TNF-Induced Pro-Survival Signaling By Regulating NF-κB Activation


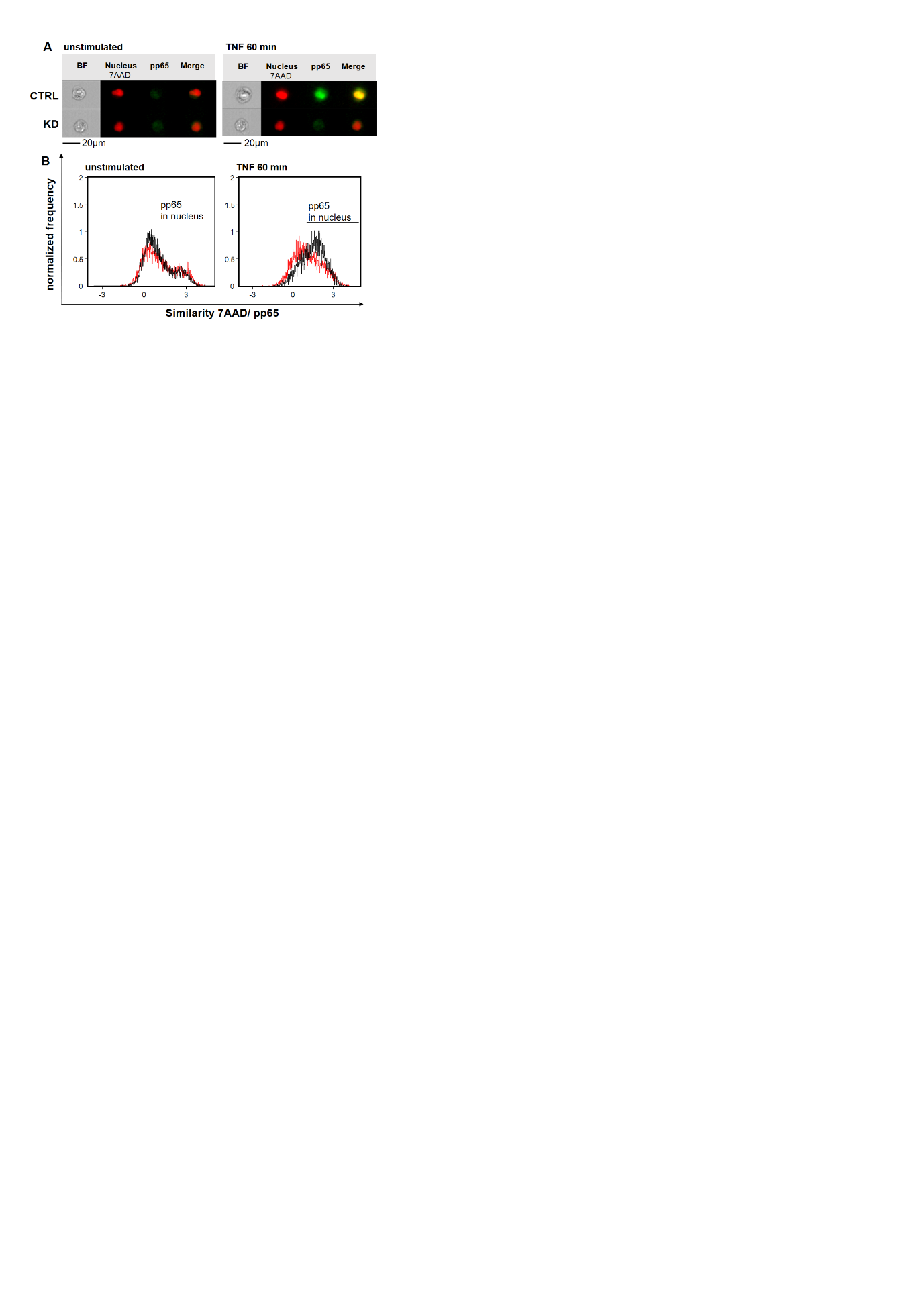


**Figure S1.** YB-1 is essential for TNF-induced NF-κB nuclear translocation in a human monocytic cell line (THP-1). (**A**) Imaging flow cytometry showing one representative cell. The nucleus stains positive for 7AAD (red). Staining for NF-kB p65 activation (pp65) is shown with and without stimulation (green). Colocalization (yellow) indicates nuclear translocation. (**B**) Normalized frequency of CTRL (black) and YB-1 KD cells (red) showing nuclear translocation of activated NF‑κB p65 with and without TNF stimulation (20 ng/mL).


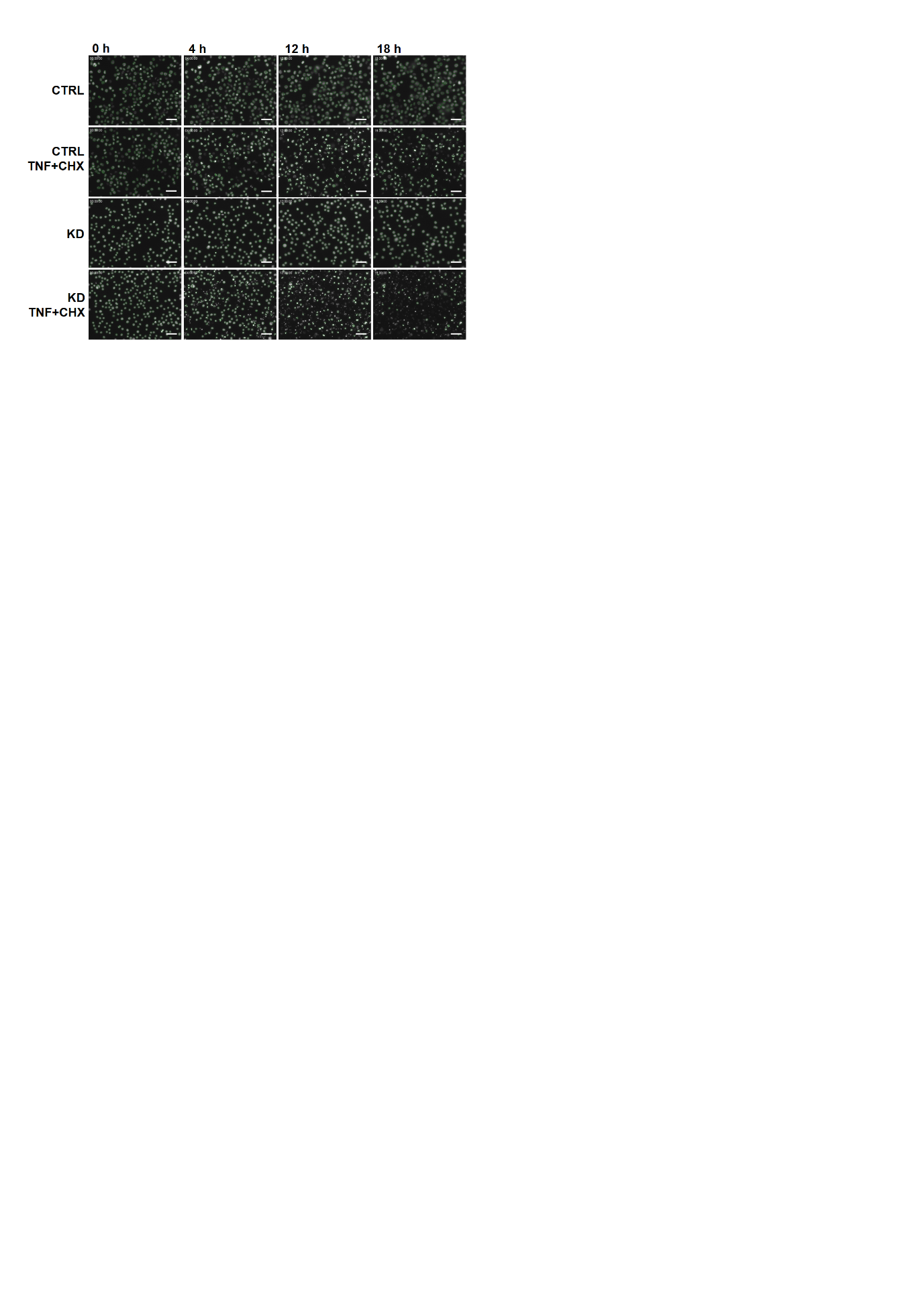


**Figure S2.** Representative single images each selected from fields of view from the time lapse microscopy. The knockdown cells showing significant cell death compared to the control cells only after treatment. The similar trend is also reflected in the reduced number of tiny DNA particles and their lower distribution over the fields of view at the end of the 18 h time-lapse microscopy. Scale bar 50 µm. Supplementary movies are also provided.


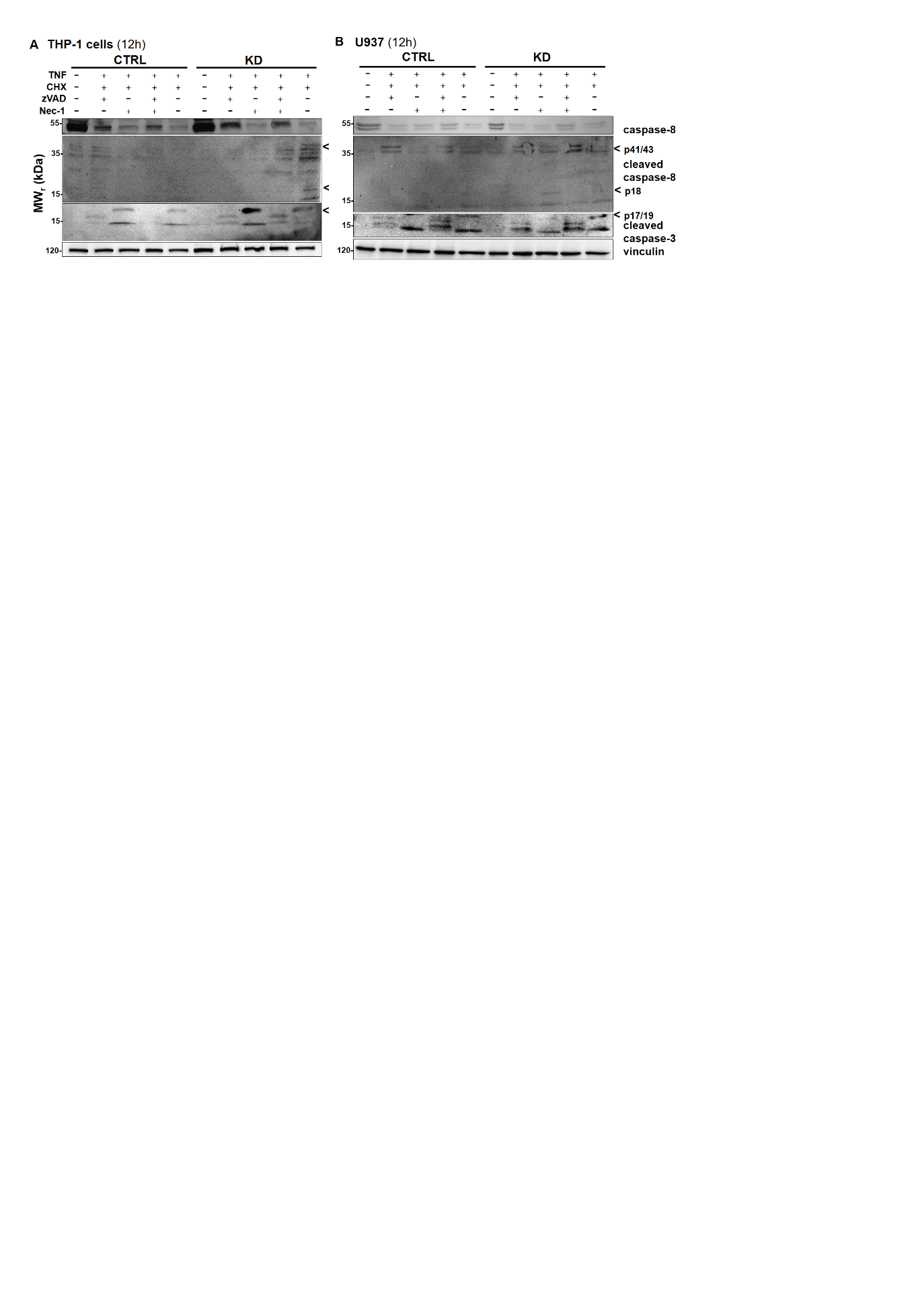


**Figure S3.** YB-1 knockdown enhances caspase activation. (A-B) Western blot analyses of caspase activation in THP-1 (**A**) and U937 cells (**B**). Cells were pretreated with zVAD and/or Nec1 for 1 hour, and treated with TNF (20 ng/mL) and cyclohexamide (CHX; 10 ng/mL) for 12 hours.

**Figure S4.** YB-1 knockdown enhanced caspase activation; whole blots are provided.

**Video S1:** CTRL**. Video S2**: KD. **Video S3**: CTRL TNF + CHX. **Video S4**: KD TNF + CHX.
