# Supplementary material for: YB-1 Mediates TNF-Induced Pro-Survival Signaling by Regulating NF-κB Activation
Source: Cancers (Basel). 2020 Aug 5;12(8):2188. doi: 10.3390/cancers12082188 (PMC7464034; doi:10.3390/cancers12082188)
Supplement: Supplementary file 1 [file cancers-12-02188-s001.zip › Figure S4 Western blots/THP1/Quantification/p-Ikba.pdf]

Single Lane Report with Profile Project 2019-10-18 Stimulation 30 60#pp65-p-Ikba,13-7.

Project Data:

Name:	2019-10-18 Stimulation 30 60#pp65-p-Ikba,13-7. scan_raw
Project Status:	private
User:	anshah
Date:	28.05.2020, 10:07
Created at:	28.05.2020, 10:07
Type of Project:	Protein Gel
Comment:	No Arguments

Gel Image:

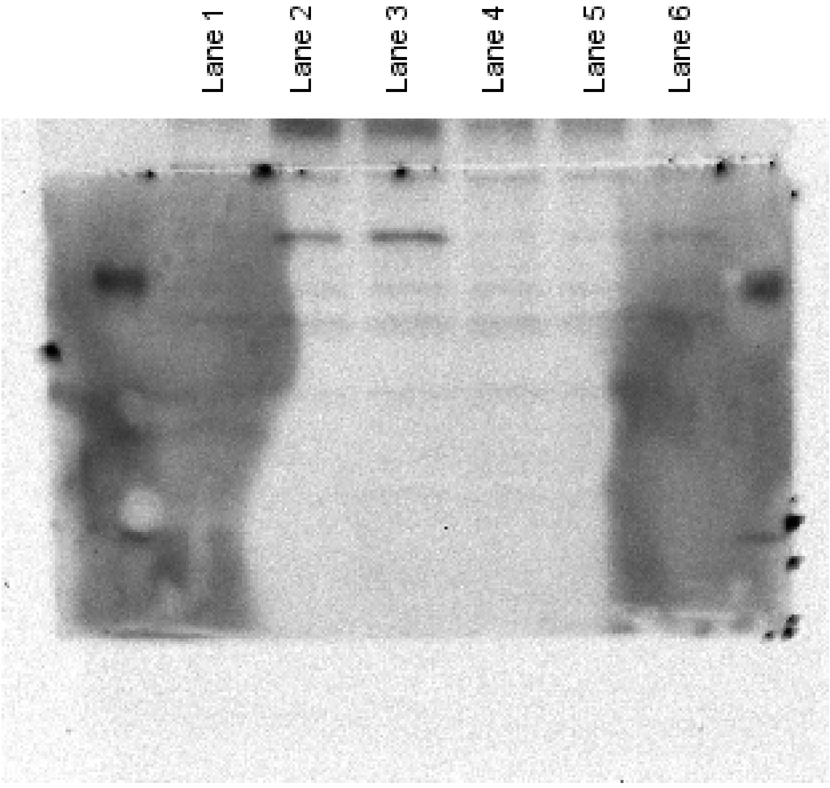

Lane 1: Lane 1

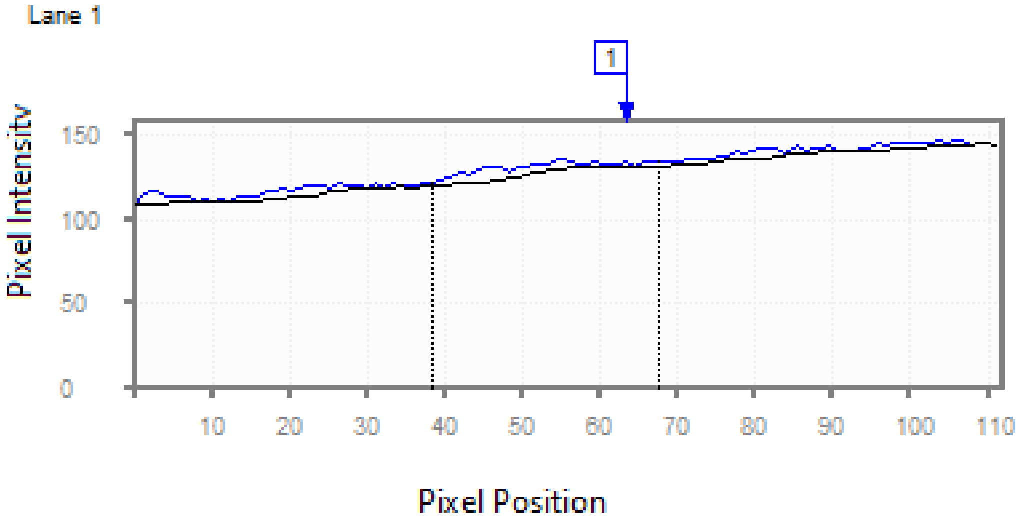

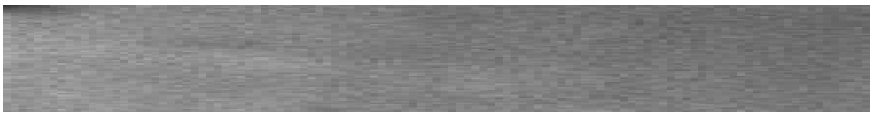

Method: Rolling Ball, Parameter: 20

Band Nr.	Band N.	Band Vol.	Backgr. Vol.	RF	MW
Band 1	1	8,439.000	245,310.000	0.568	--

Band Nr.	Cal. Band Vol.
Band 1	0.000

Lane 2: Lane 2

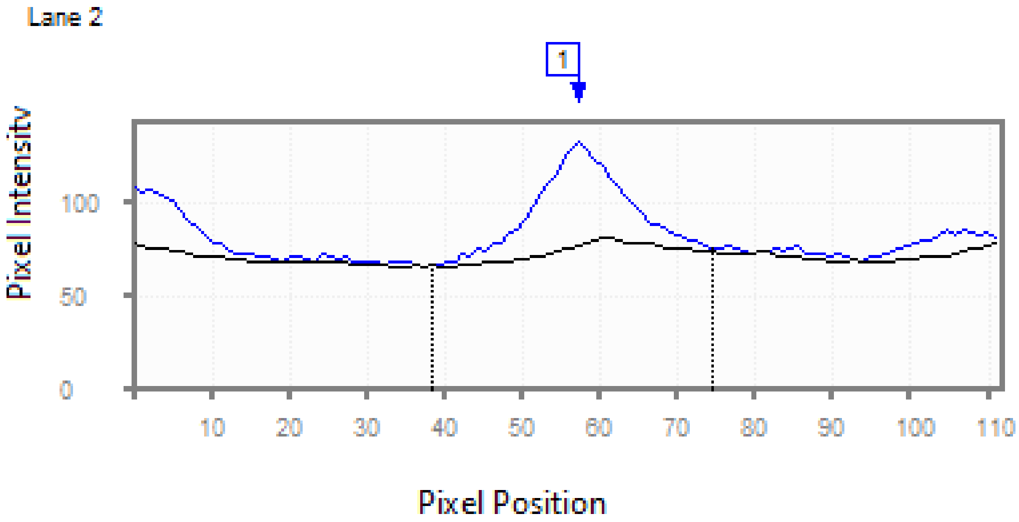

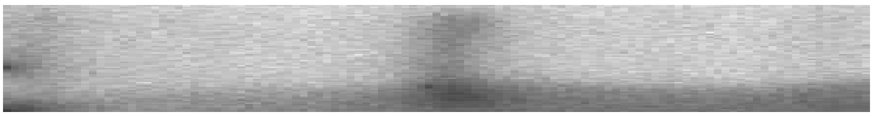

Method: Rolling Ball, Parameter: 20

Band Nr.	Band N.	Band Vol.	Backgr. Vol.	RF	MW
Band 1	1	48,513.000	176,823.000	0.514	--

Band Nr.	Cal. Band Vol.
Band 1	0.000

Lane 3: Lane 3

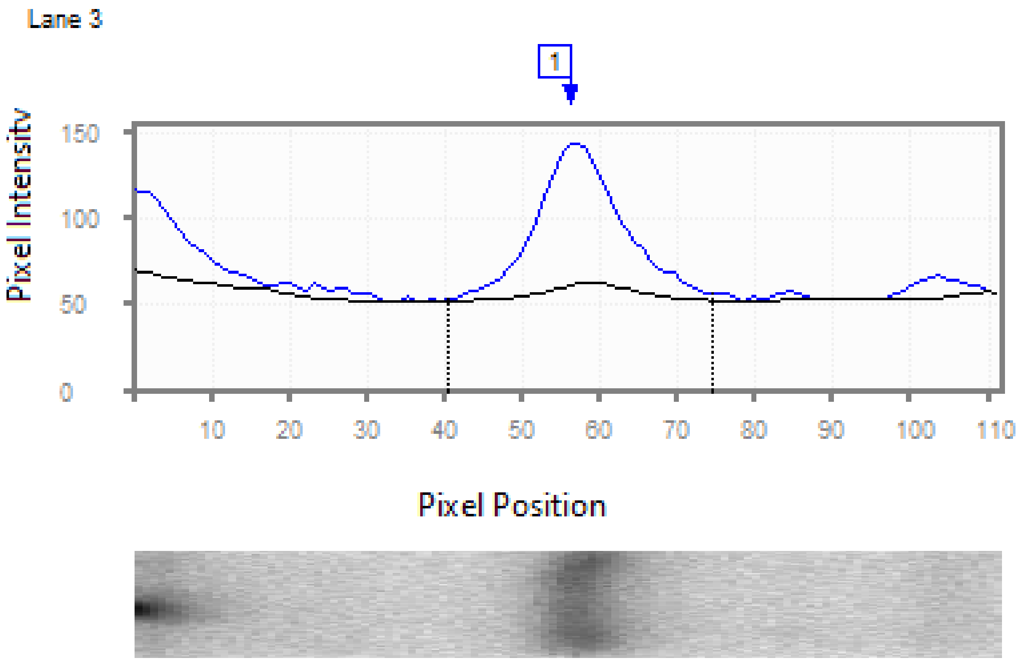

Method: Rolling Ball, Parameter: 20

Band Nr.	Band N.	Band Vol.	Backgr. Vol.	RF	MW
Band 1	1	70,117.000	128,009.000	0.505	--

Band Nr.	Cal. Band Vol.
Band 1	0.000

Lane 4: Lane 4

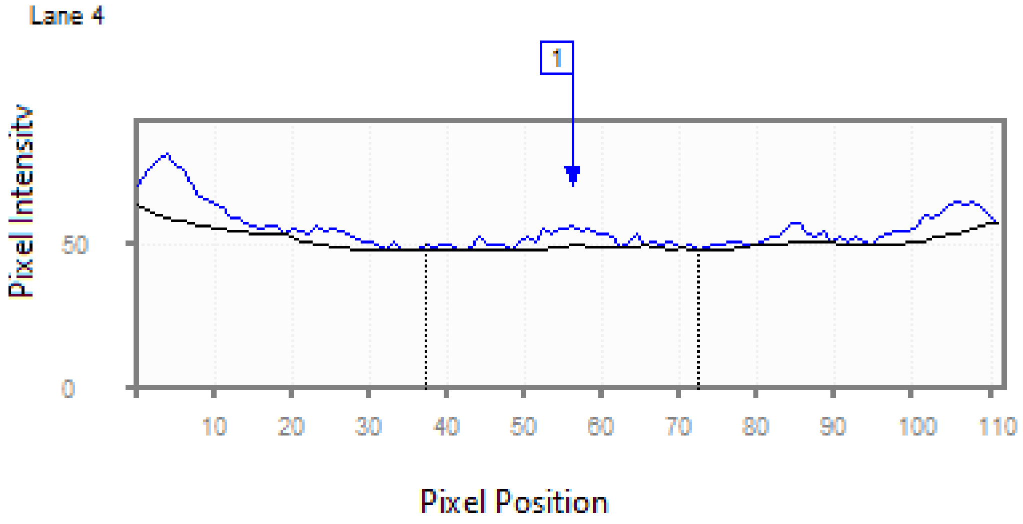

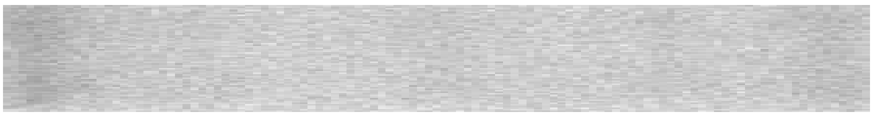

Method: Rolling Ball, Parameter: 20

Band Nr.	Band N.	Band Vol.	Backgr. Vol.	RF	MW
Band 1	1	6,333.000	112,977.000	0.505	--

Band Nr.	Cal. Band Vol.
Band 1	0.000

Lane 5: Lane 5

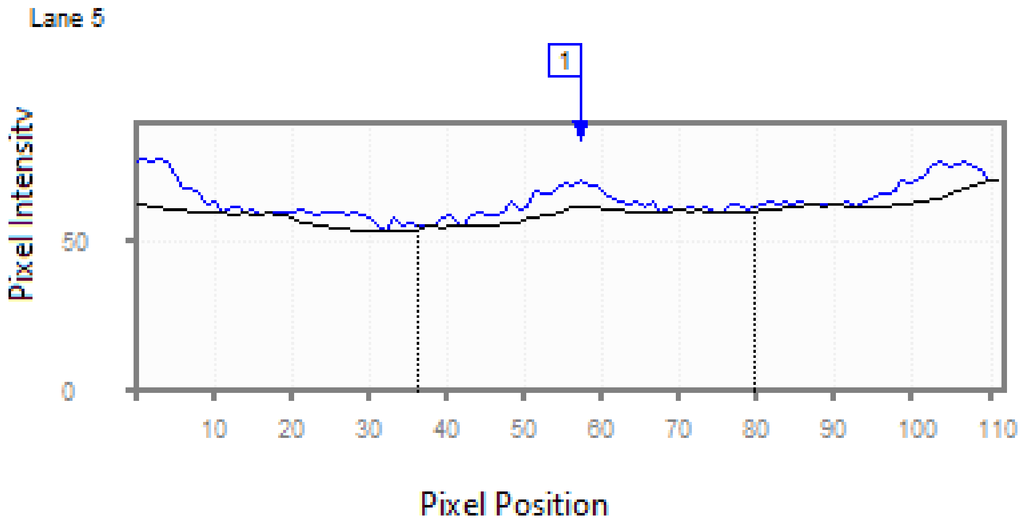

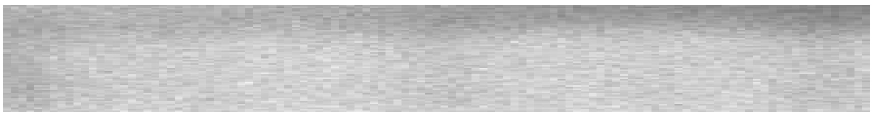

Method: Rolling Ball, Parameter: 20

Band Nr.	Band N.	Band Vol.	Backgr. Vol.	RF	MW
Band 1	1	10,172.000	169,193.000	0.514	--

Band Nr.	Cal. Band Vol.
Band 1	0.000

Lane 6: Lane 6

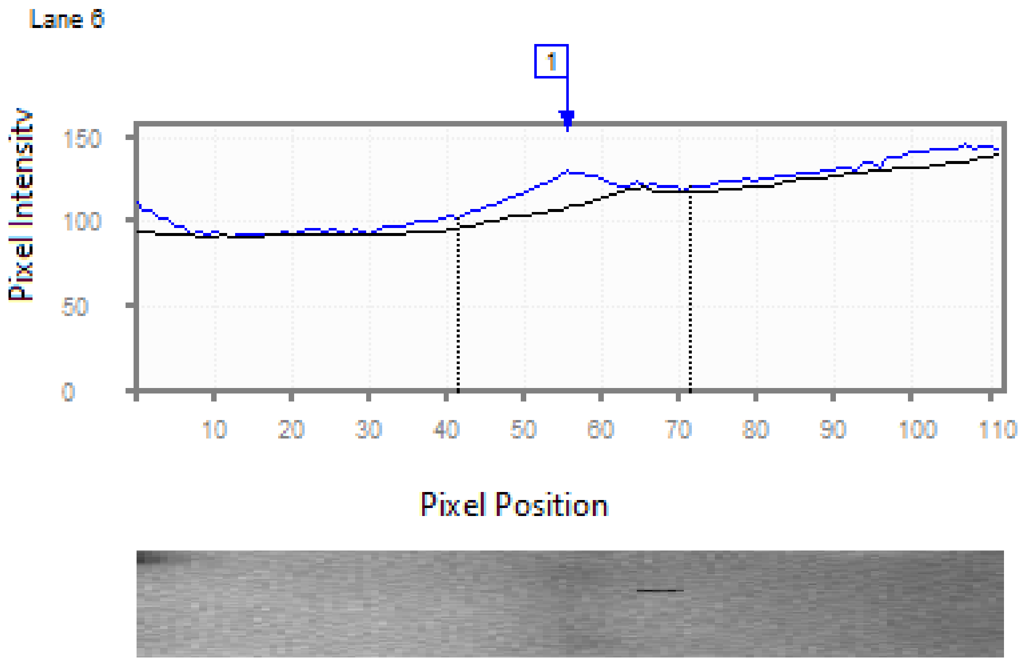

Method: Rolling Ball, Parameter: 20

Band Nr.	Band N.	Band Vol.	Backgr. Vol.	RF	MW
Band 1	1	19,343.000	219,052.000	0.495	--

Band Nr.	Cal. Band Vol.
Band 1	0.000
