# Supplementary material for: YB-1 Mediates TNF-Induced Pro-Survival Signaling by Regulating NF-κB Activation
Source: Cancers (Basel). 2020 Aug 5;12(8):2188. doi: 10.3390/cancers12082188 (PMC7464034; doi:10.3390/cancers12082188)
Supplement: Supplementary file 1 [file cancers-12-02188-s001.zip › Figure S4 Western blots/THP1/Quantification/p65.pdf]

Single Lane Report with Profile Project p65

Project Data:

Name:	p65
Project Status:	private
User:	anshah
Date:	28.05.2020, 09:48
Created at:	28.05.2020, 09:48
Type of Project:	Protein Gel
Comment:	No Arguments

Gel Image:

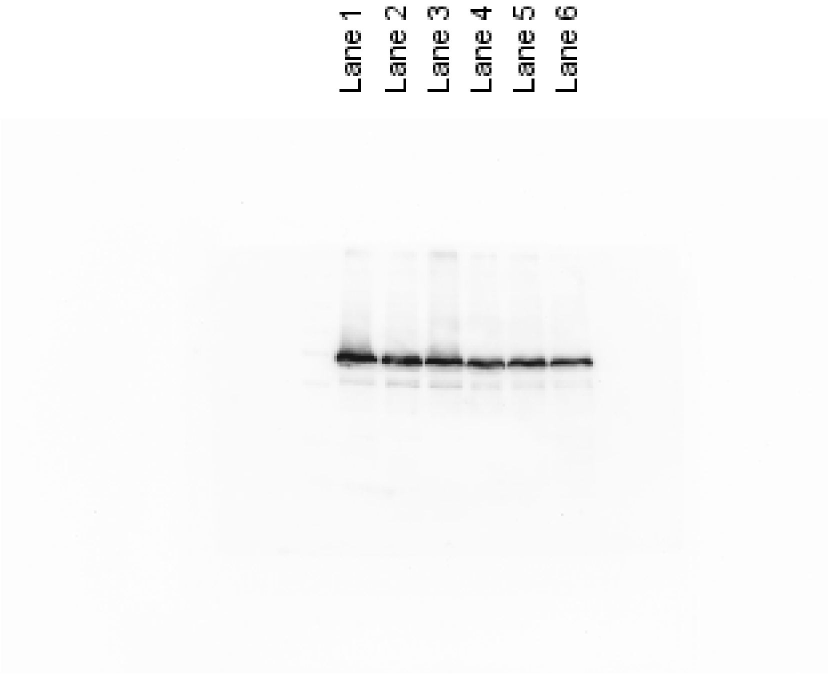

Lane 1: Lane 1

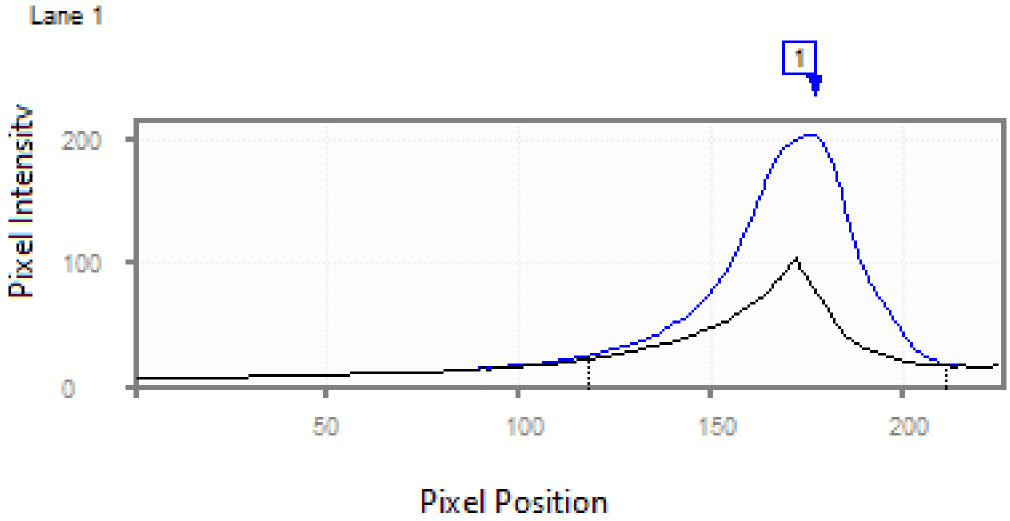

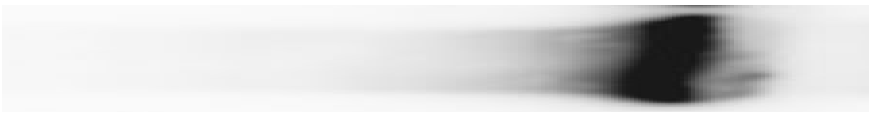

Method: Rolling Ball, Parameter: 20

Band Nr.	Band N.	Band Vol.	Backgr. Vol.	RF	MW
Band 1	1	560,102.000	545,429.000	0.782	--

Band Nr.	Cal. Band Vol.
Band 1	0.000

Lane 2: Lane 2

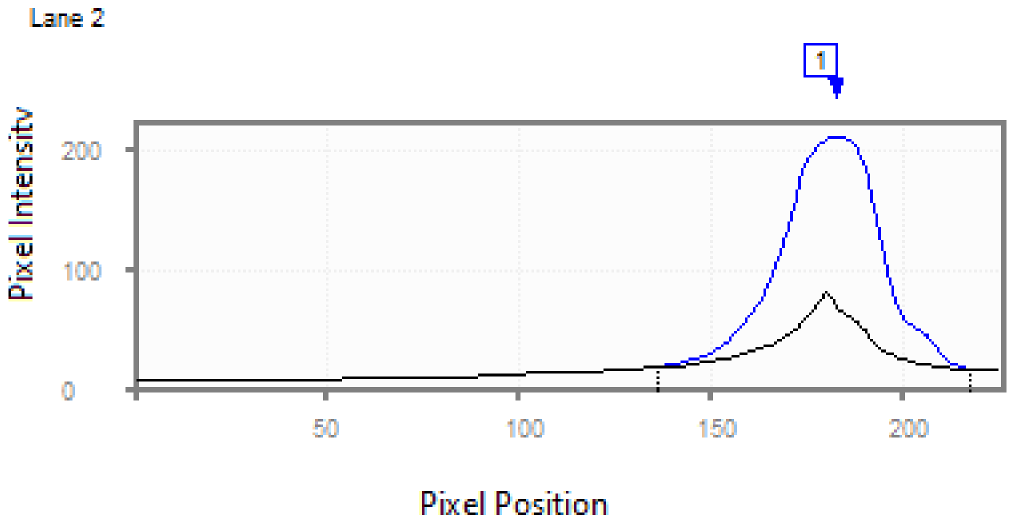

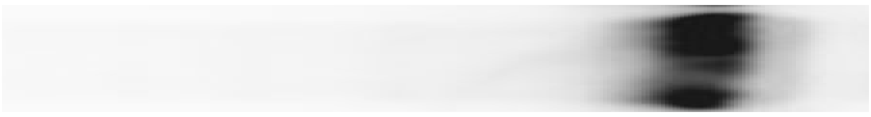

Method: Rolling Ball, Parameter: 20

Band Nr.	Band N.	Band Vol.	Backgr. Vol.	RF	MW
Band 1	1	517,709.000	330,183.000	0.809	--

Band Nr.	Cal. Band Vol.
Band 1	0.000

Lane 3: Lane 3

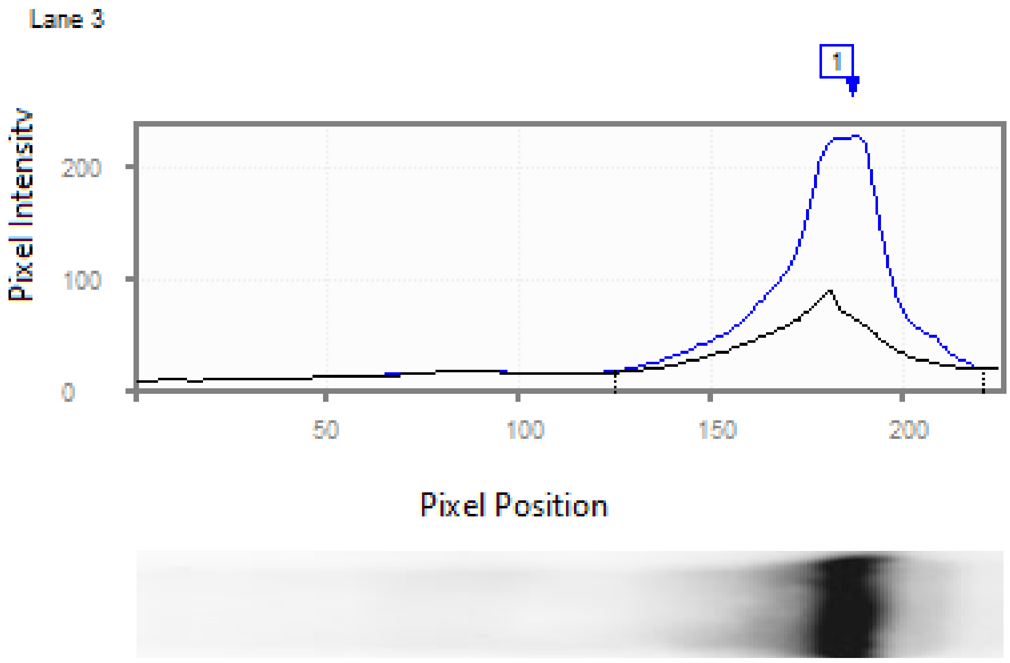

Method: Rolling Ball, Parameter: 20

Band Nr.	Band N.	Band Vol.	Backgr. Vol.	RF	MW
Band 1	1	438,187.000	384,429.000	0.827	--

Band Nr.	Cal. Band Vol.
Band 1	0.000

Lane 4: Lane 4

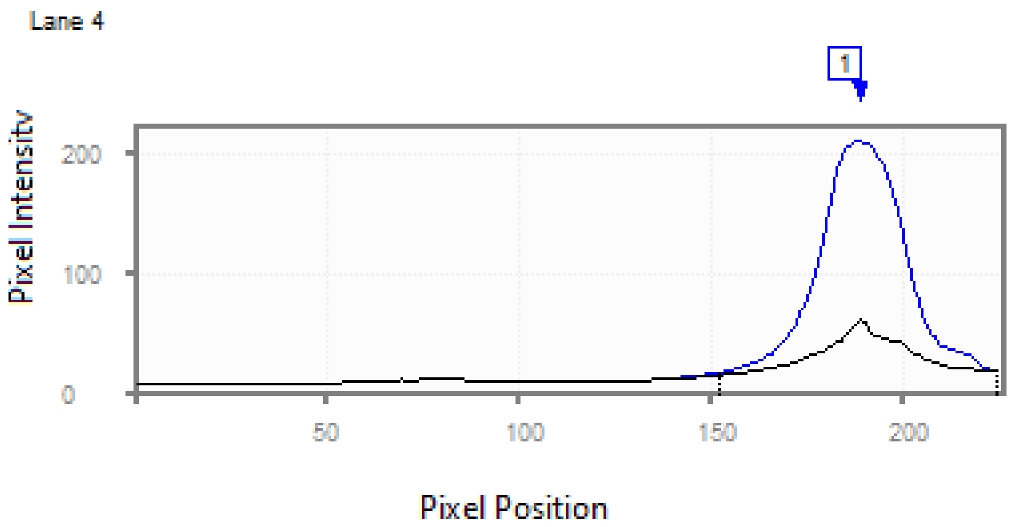

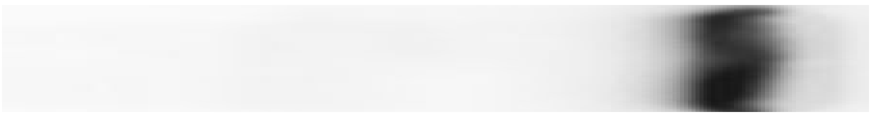

Method: Rolling Ball, Parameter: 20

Band Nr.	Band N.	Band Vol.	Backgr. Vol.	RF	MW
Band 1	1	405,963.000	222,797.000	0.836	--

Band Nr.	Cal. Band Vol.
Band 1	0.000

Lane 5: Lane 5

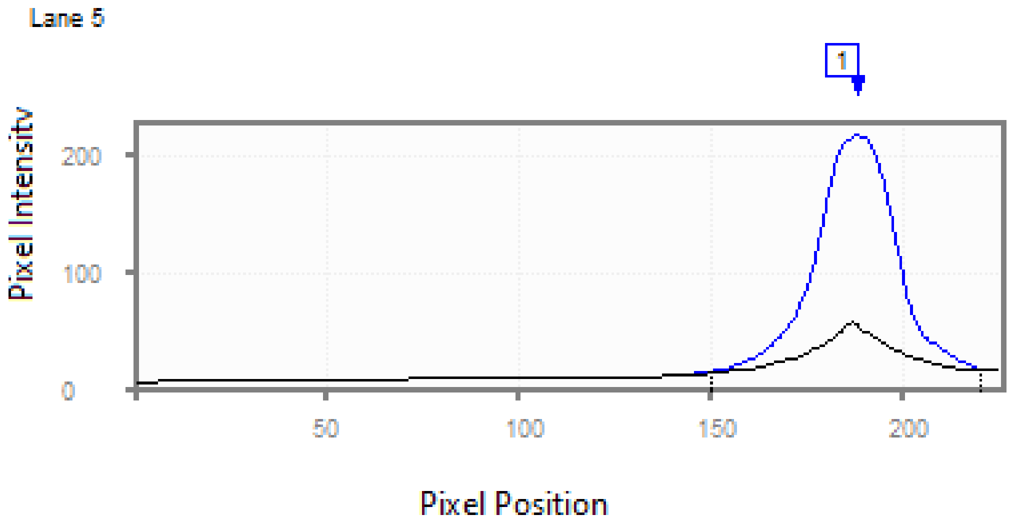

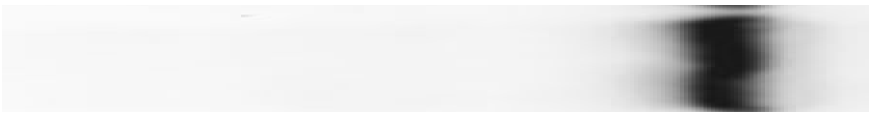

Method: Rolling Ball, Parameter: 20

Band Nr.	Band N.	Band Vol.	Backgr. Vol.	RF	MW
Band 1	1	440,218.000	224,647.000	0.831	--

Band Nr.	Cal. Band Vol.
Band 1	0.000

Lane 6: Lane 6

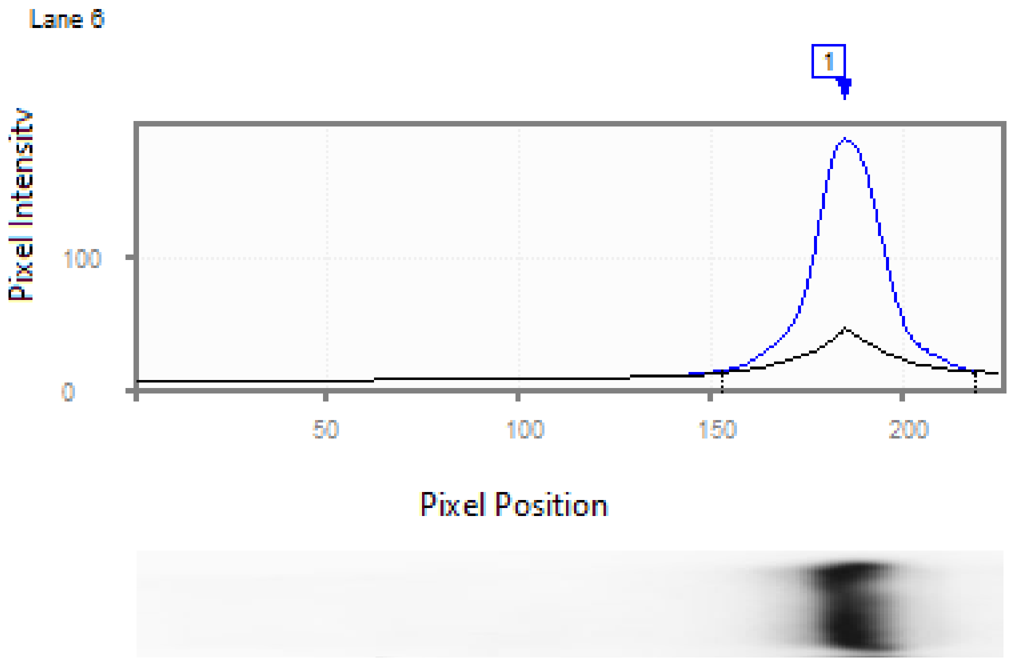

Method: Rolling Ball, Parameter: 20

Band Nr.	Band N.	Band Vol.	Backgr. Vol.	RF	MW
Band 1	1	417,178.000	214,077.000	0.818	--

Band Nr.	Cal. Band Vol.
Band 1	0.000
