# Supplementary material for: YB-1 Mediates TNF-Induced Pro-Survival Signaling by Regulating NF-κB Activation
Source: Cancers (Basel). 2020 Aug 5;12(8):2188. doi: 10.3390/cancers12082188 (PMC7464034; doi:10.3390/cancers12082188)
Supplement: Supplementary file 1 [file cancers-12-02188-s001.zip › Figure S4 Western blots/THP1/Quantification/TNFR1.pdf]

Single Lane Report with Profile Project 2020-01-14 THP1 stimulation # TNFR1,19-10.

Project Data:

Name:	2020-01-14 THP1 stimulation # TNFR1,19-10. scan_raw
Project Status:	private
User:	anshah
Date:	28.05.2020, 09:54
Created at:	28.05.2020, 09:54
Type of Project:	Protein Gel
Comment:	No Arguments

Gel Image:

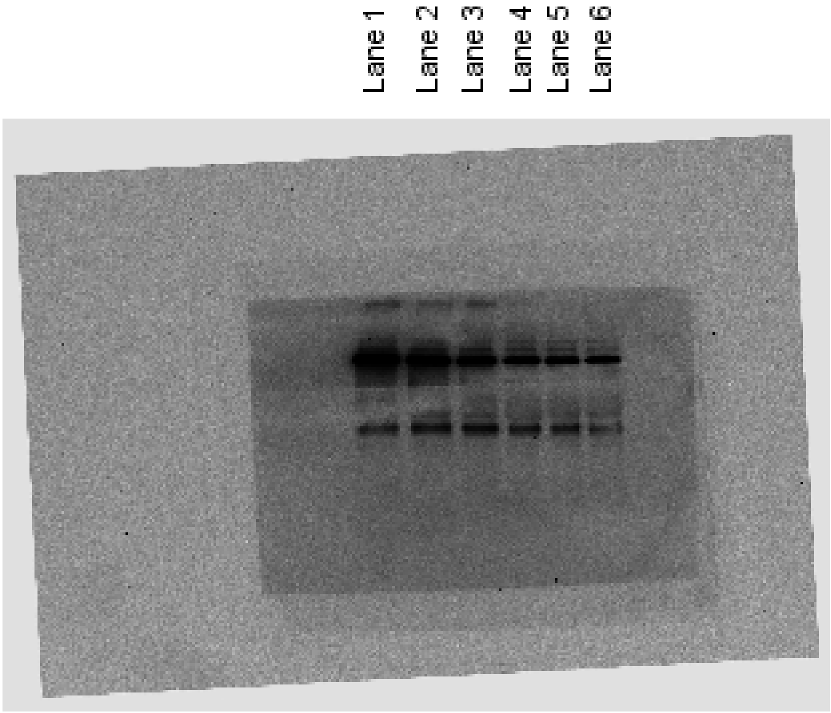

Lane 1: Lane 1

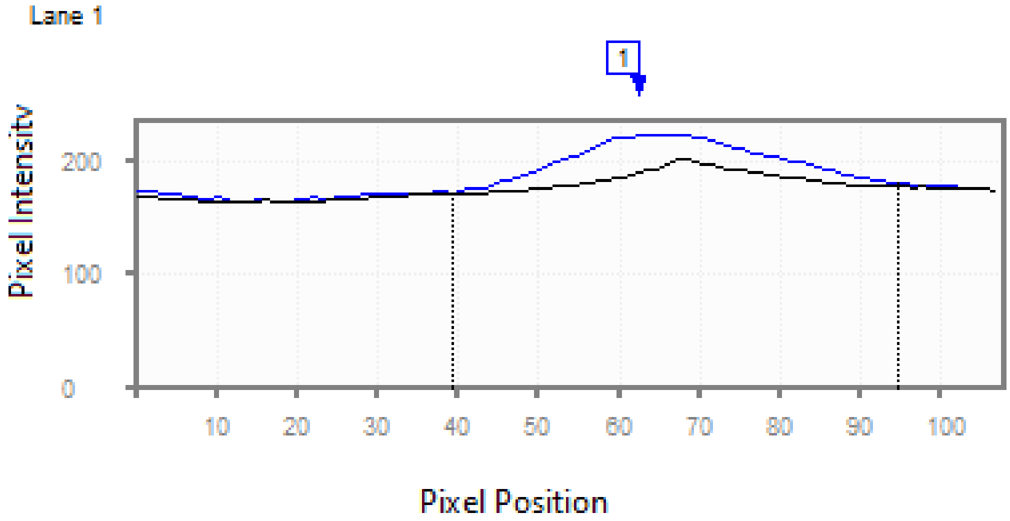

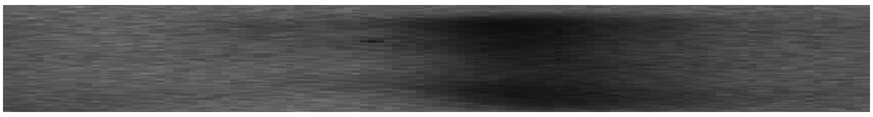

Method: Rolling Ball, Parameter: 20

Band Nr.	Band N.	Band Vol.	Backgr. Vol.	RF	MW
Band 1	1	123,669.000	1,316,670.000	0.579	--

Band Nr.	Cal. Band Vol.
Band 1	0.000

Lane 2: Lane 2

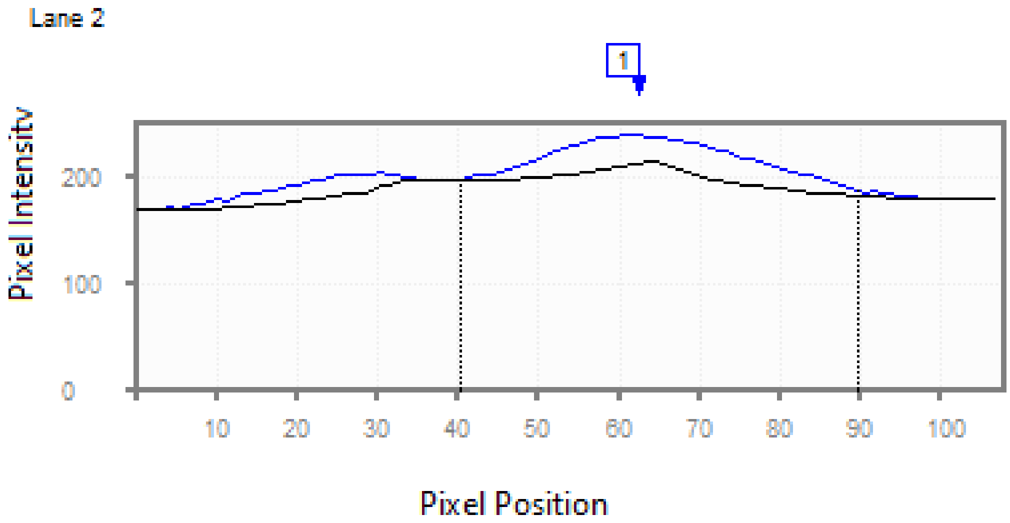

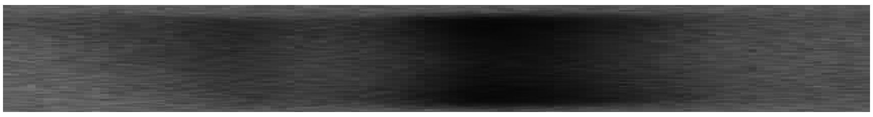

Method: Rolling Ball, Parameter: 20

Band Nr.	Band N.	Band Vol.	Backgr. Vol.	RF	MW
Band 1	1	124,336.000	1,258,761.000	0.579	--

Band Nr.	Cal. Band Vol.
Band 1	0.000

Lane 3: Lane 3

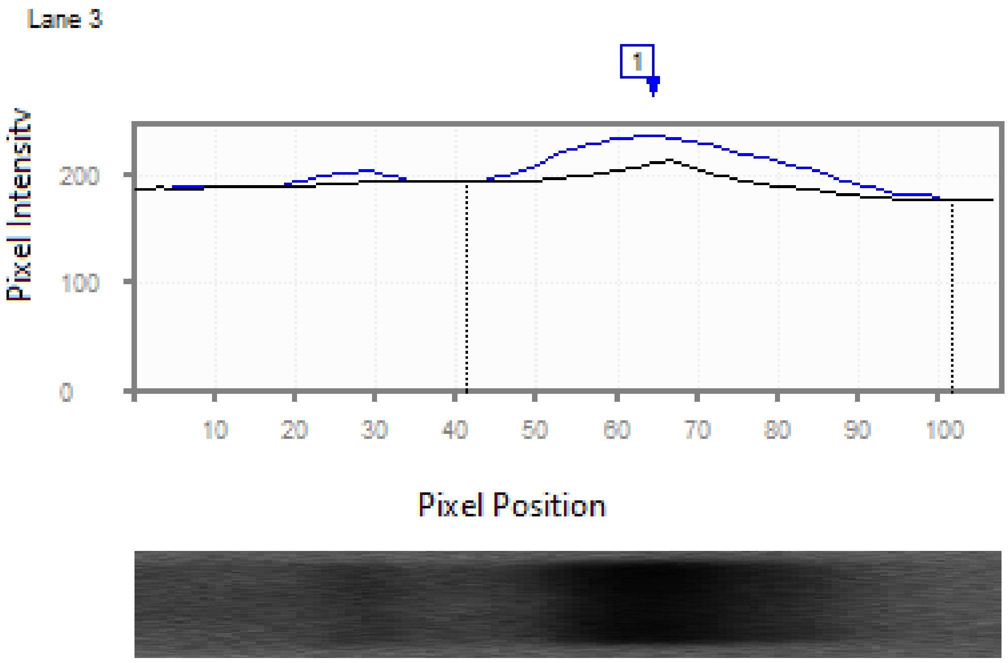

Method: Rolling Ball, Parameter: 20

Band Nr.	Band N.	Band Vol.	Backgr. Vol.	RF	MW
Band 1	1	130,595.000	1,498,476.000	0.598	--

Band Nr.	Cal. Band Vol.
Band 1	0.000

Lane 4: Lane 4

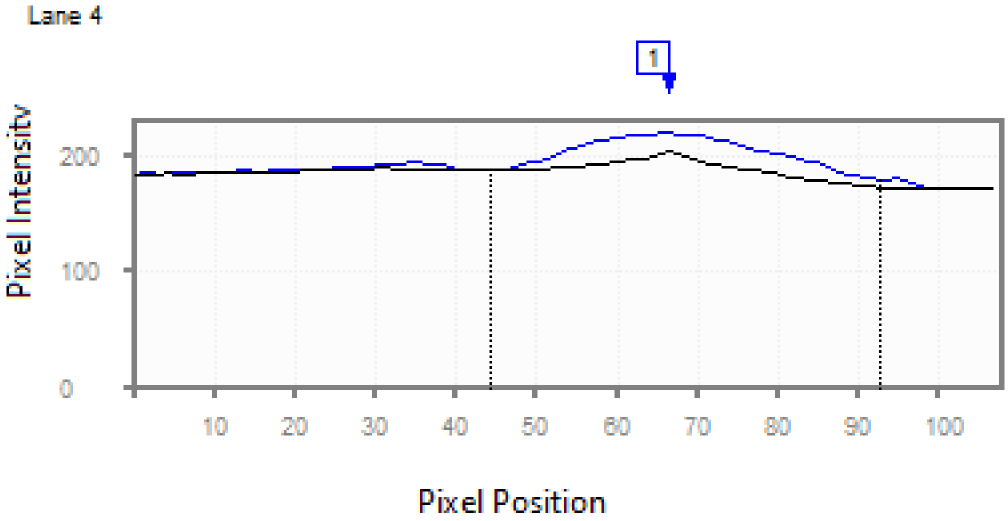

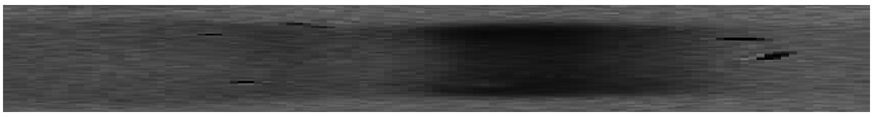

Method: Rolling Ball, Parameter: 20

Band Nr.	Band N.	Band Vol.	Backgr. Vol.	RF	MW
Band 1	1	93,150.000	1,175,694.000	0.617	--

Band Nr.	Cal. Band Vol.
Band 1	0.000

Lane 5: Lane 5

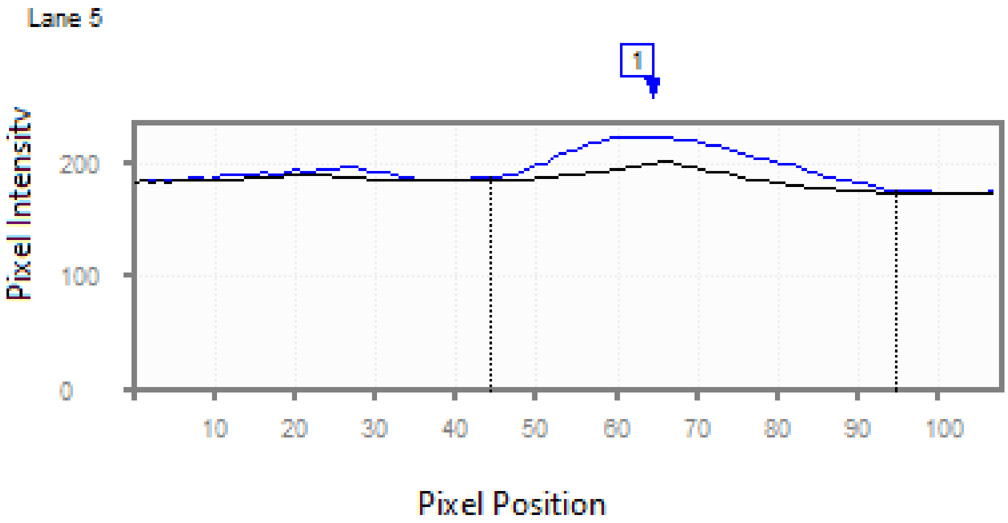

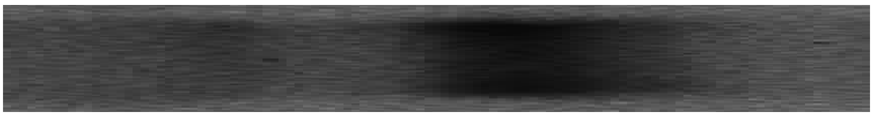

Method: Rolling Ball, Parameter: 20

Band Nr.	Band N.	Band Vol.	Backgr. Vol.	RF	MW
Band 1	1	95,339.000	1,049,512.000	0.598	--

Band Nr.	Cal. Band Vol.
Band 1	0.000

Lane 6: Lane 6

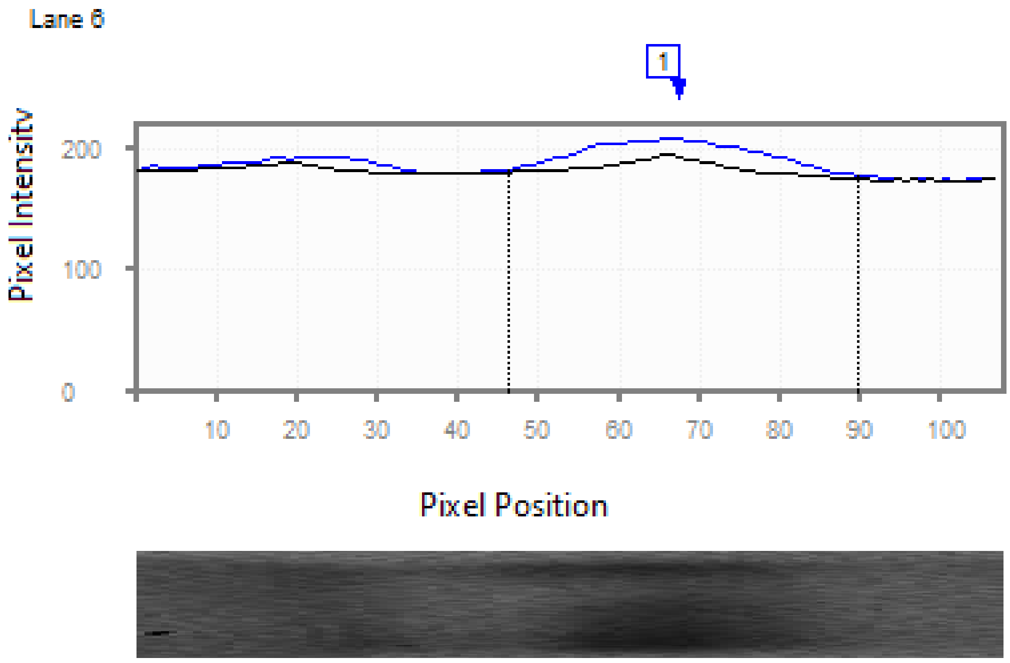

Method: Rolling Ball, Parameter: 20

Band Nr.	Band N.	Band Vol.	Backgr. Vol.	RF	MW
Band 1	1	59,025.000	883,119.000	0.626	--

Band Nr.	Cal. Band Vol.
Band 1	0.000
