# Supplementary material for: YB-1 Mediates TNF-Induced Pro-Survival Signaling by Regulating NF-κB Activation
Source: Cancers (Basel). 2020 Aug 5;12(8):2188. doi: 10.3390/cancers12082188 (PMC7464034; doi:10.3390/cancers12082188)
Supplement: Supplementary file 1 [file cancers-12-02188-s001.zip › Figure S4 Western blots/THP1/Quantification/total Ikba.pdf]

Single Lane Report with Profile Project total Ikba

Project Data:

Name:	total Ikba
Project Status:	private
User:	anshah
Date:	28.05.2020, 09:51
Created at:	28.05.2020, 09:51
Type of Project:	Protein Gel
Comment:	No Arguments

Gel Image:

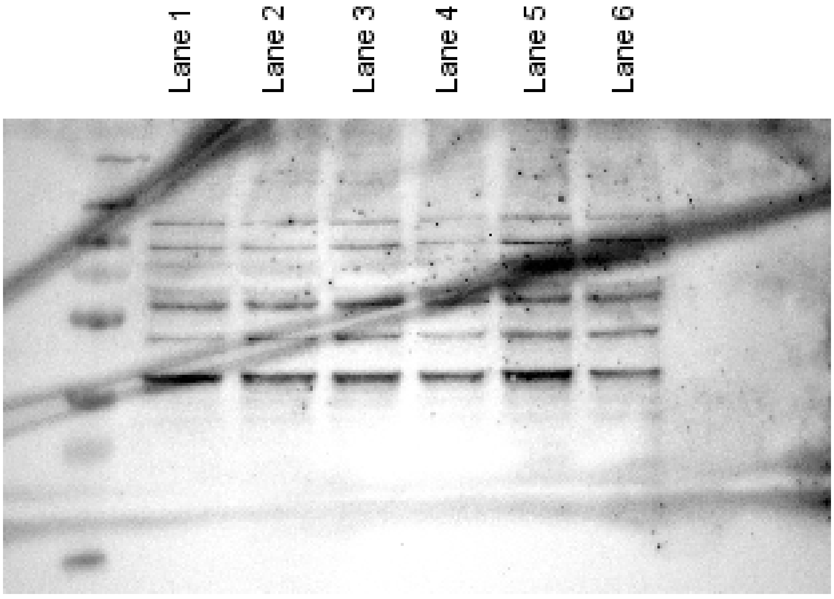

Lane 1: Lane 1

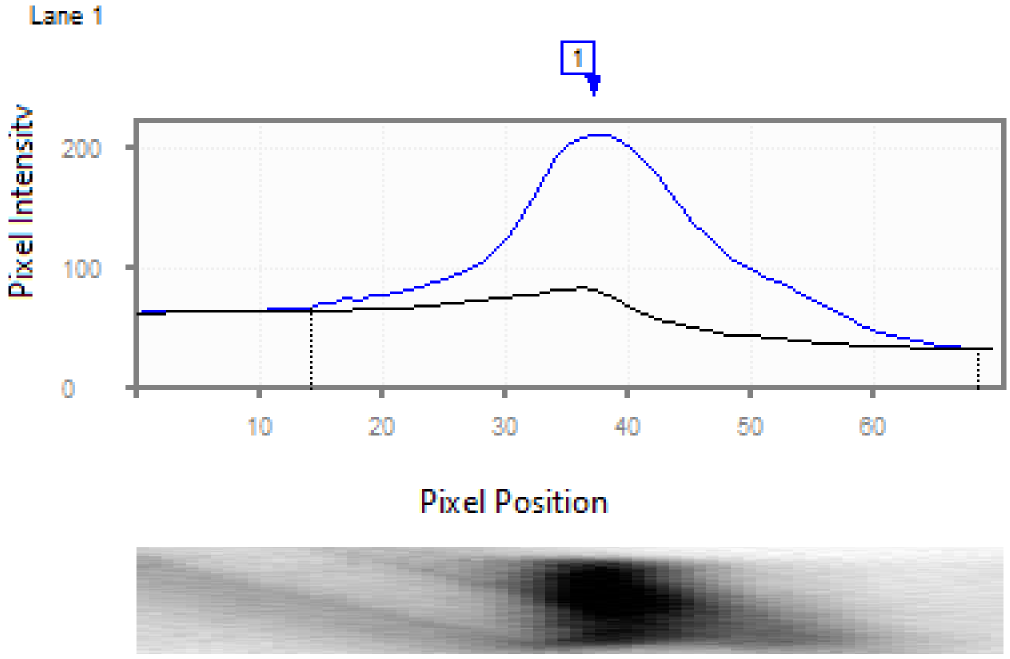

Method: Rolling Ball, Parameter: 20

Band Nr.	Band N.	Band Vol.	Backgr. Vol.	RF	MW
Band 1	1	344,917.000	396,972.000	0.529	--

Band Nr.	Cal. Band Vol.
Band 1	0.000

Lane 2: Lane 2

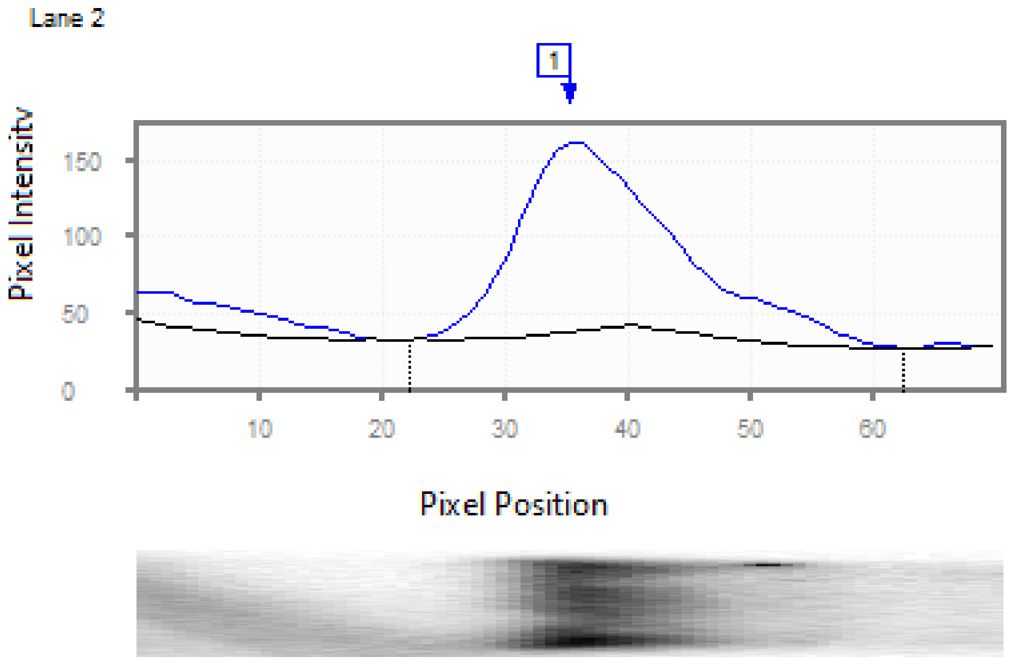

Method: Rolling Ball, Parameter: 20

Band Nr.	Band N.	Band Vol.	Backgr. Vol.	RF	MW
Band 1	1	217,087.000	162,950.000	0.500	--

Band Nr.	Cal. Band Vol.
Band 1	0.000

Lane 3: Lane 3

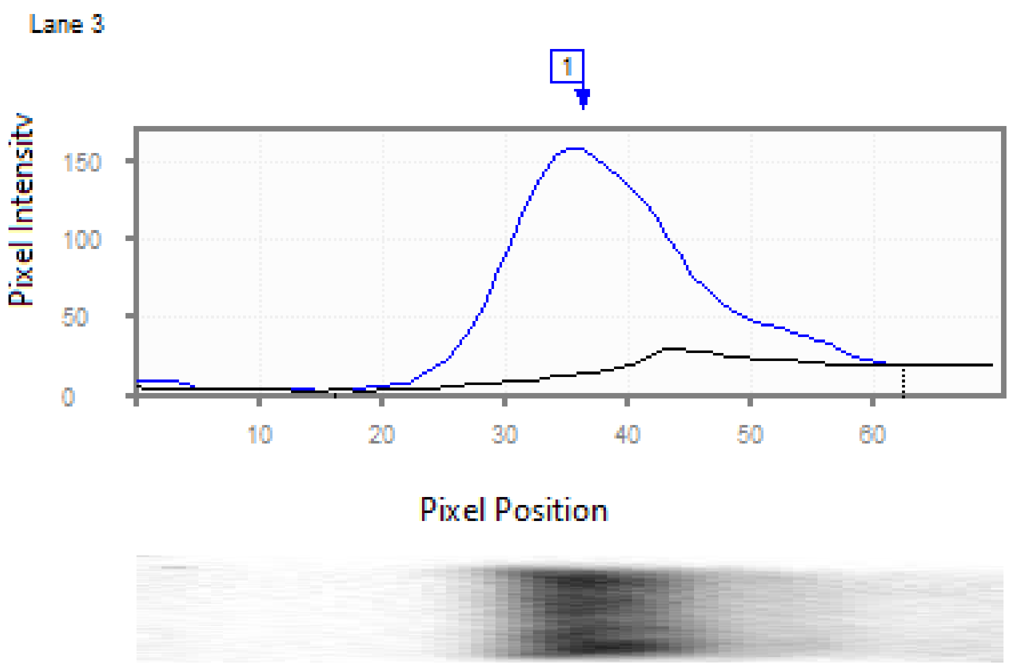

Method: Rolling Ball, Parameter: 20

Band Nr.	Band N.	Band Vol.	Backgr. Vol.	RF	MW
Band 1	1	247,469.000	79,123.000	0.514	--

Band Nr.	Cal. Band Vol.
Band 1	0.000

Lane 4: Lane 4

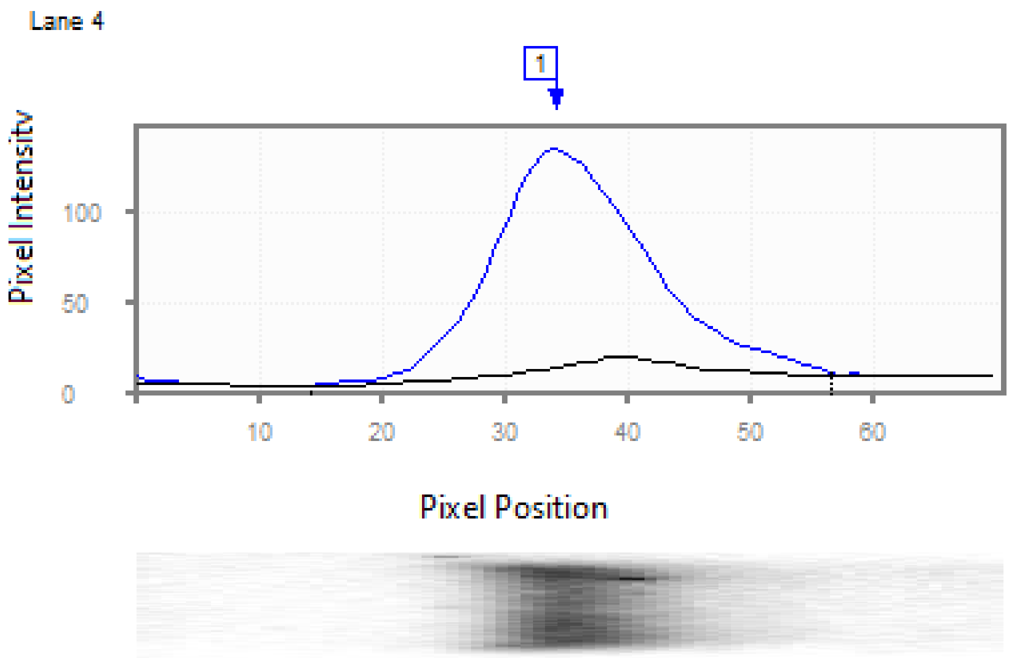

Method: Rolling Ball, Parameter: 20

Band Nr.	Band N.	Band Vol.	Backgr. Vol.	RF	MW
Band 1	1	189,251.000	51,421.000	0.486	--

Band Nr.	Cal. Band Vol.
Band 1	0.000

Lane 5: Lane 5

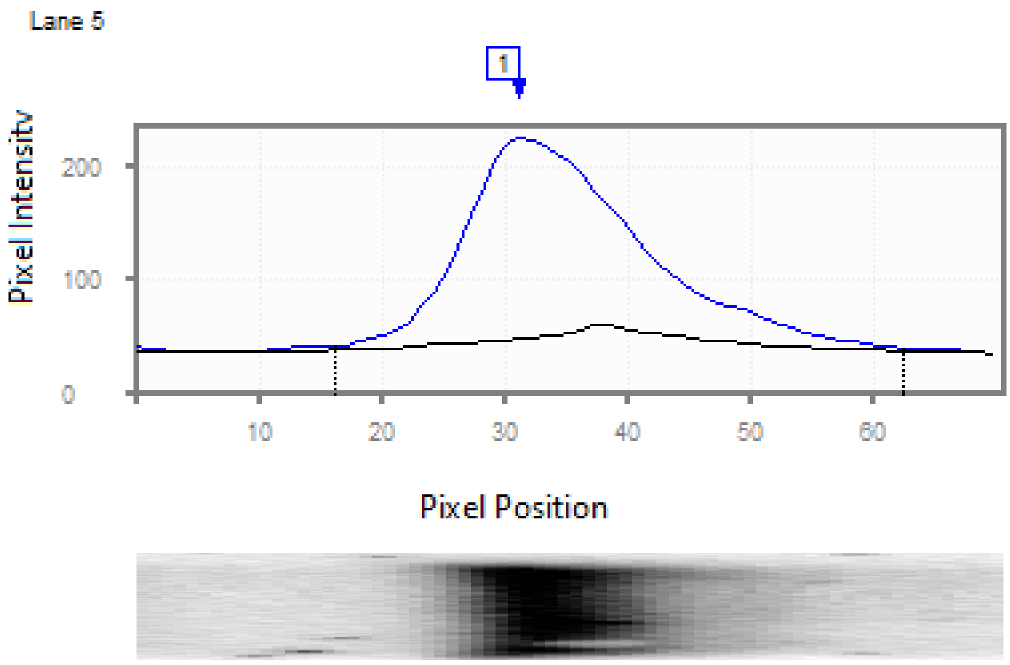

Method: Rolling Ball, Parameter: 20

Band Nr.	Band N.	Band Vol.	Backgr. Vol.	RF	MW
Band 1	1	308,639.000	228,366.000	0.443	--

Band Nr.	Cal. Band Vol.
Band 1	0.000

Lane 6: Lane 6

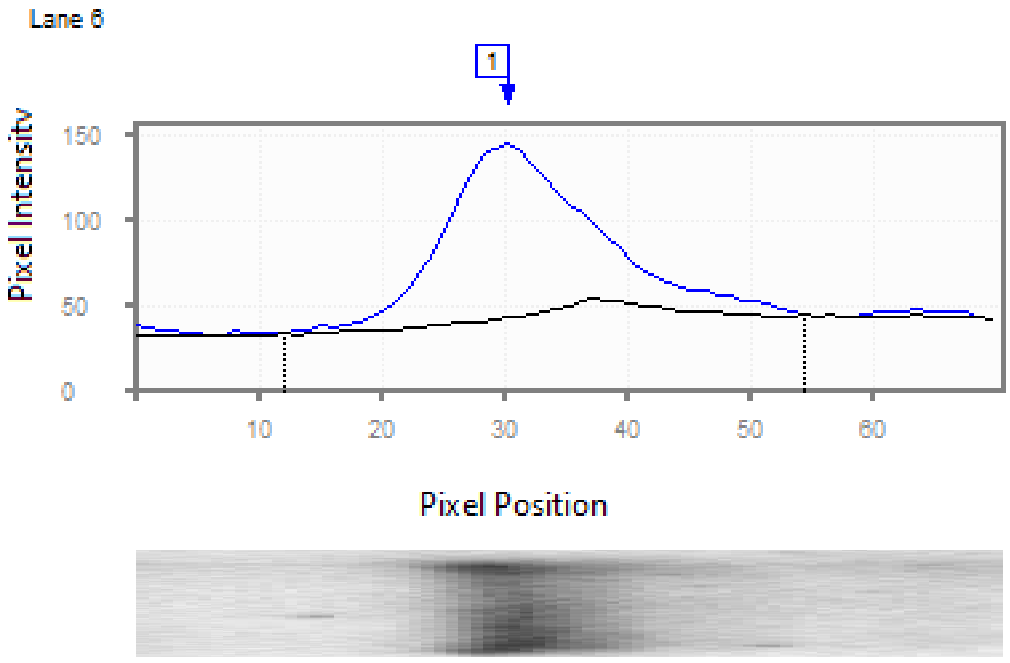

Method: Rolling Ball, Parameter: 20

Band Nr.	Band N.	Band Vol.	Backgr. Vol.	RF	MW
Band 1	1	154,348.000	196,093.000	0.429	--

Band Nr.	Cal. Band Vol.
Band 1	0.000
