# Supplementary material for: YB-1 Mediates TNF-Induced Pro-Survival Signaling by Regulating NF-κB Activation
Source: Cancers (Basel). 2020 Aug 5;12(8):2188. doi: 10.3390/cancers12082188 (PMC7464034; doi:10.3390/cancers12082188)
Supplement: Supplementary file 1 [file cancers-12-02188-s001.zip › Figure S4 Western blots/U937/Quantification/p-IkBa.pdf]

Single Lane Report with Profile Project p-IkBa

Project Data:

Name:	p-IkBa
Project Status:	private
User:	anshah
Date:	26.05.2020, 13:25
Created at:	26.05.2020, 13:25
Type of Project:	Protein Gel
Comment:	No Arguments

Gel Image:

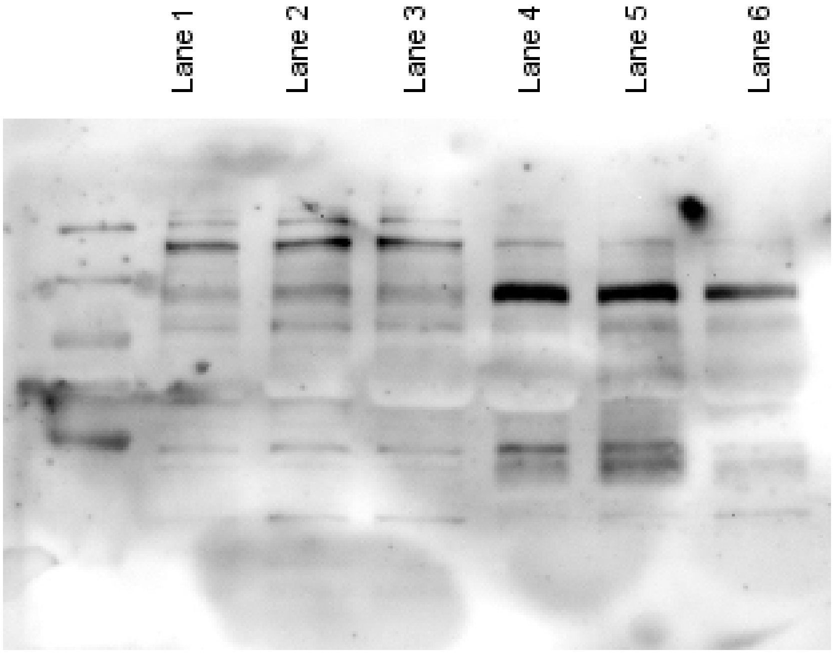

Lane 1: Lane 1

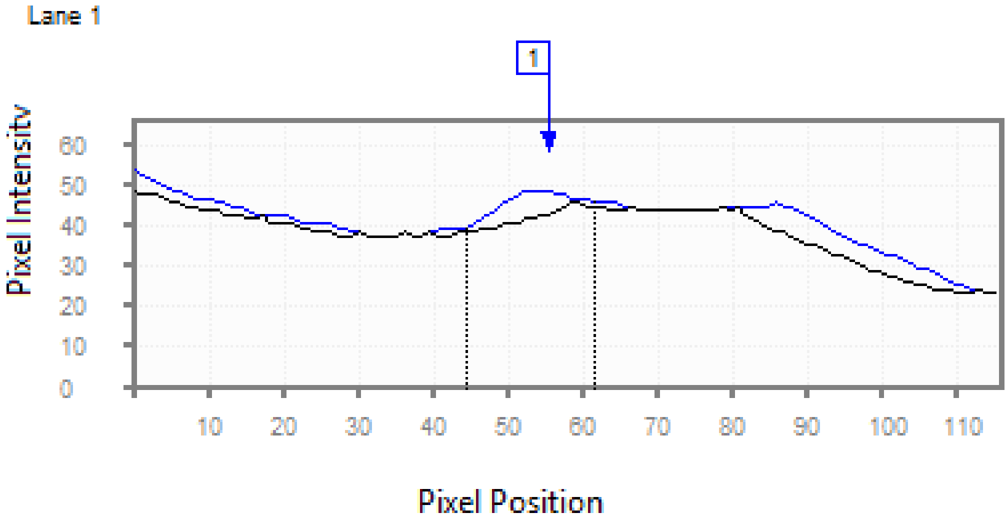

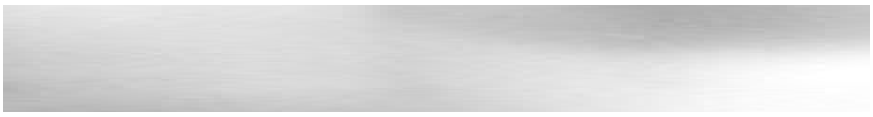

Method: Rolling Ball, Parameter: 20

Band Nr.	Band N.	Band Vol.	Backgr. Vol.	RF	MW
Band 1	1	6,337.000	68,993.000	0.478	--

Band Nr.	Cal. Band Vol.
Band 1	0.000

Lane 2: Lane 2

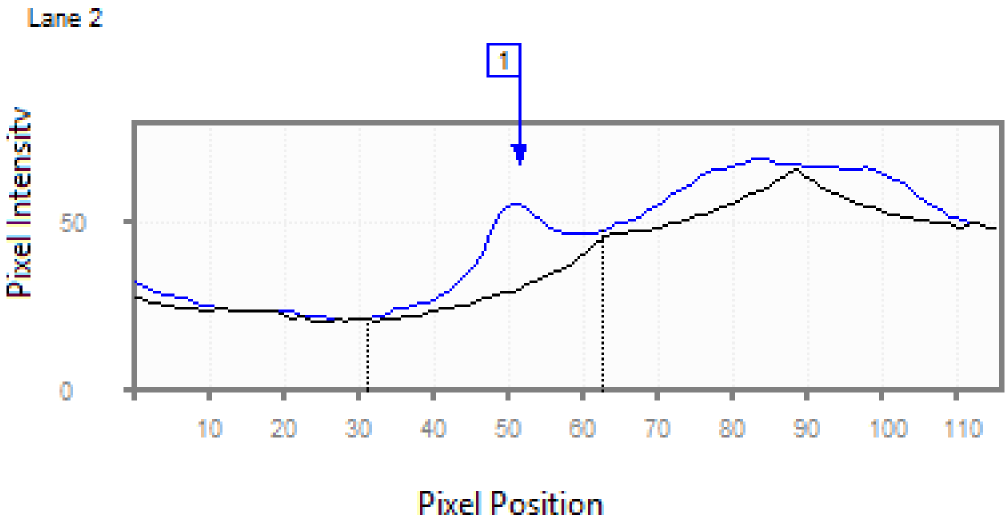

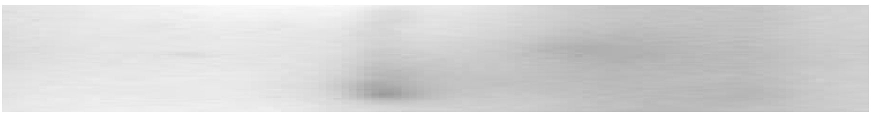

Method: Rolling Ball, Parameter: 20

Band Nr.	Band N.	Band Vol.	Backgr. Vol.	RF	MW
Band 1	1	30,341.000	85,014.000	0.443	--

Band Nr.	Cal. Band Vol.
Band 1	0.000

Lane 3: Lane 3

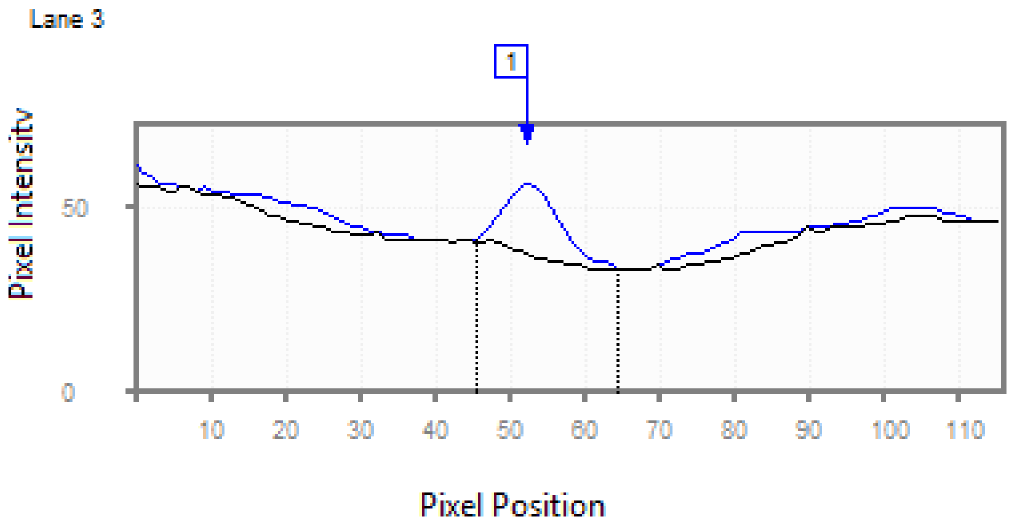

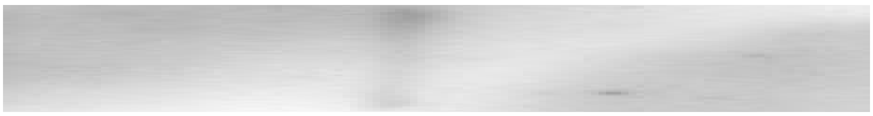

Method: Rolling Ball, Parameter: 20

Band Nr.	Band N.	Band Vol.	Backgr. Vol.	RF	MW
Band 1	1	16,205.000	67,421.000	0.452	--

Band Nr.	Cal. Band Vol.
Band 1	0.000

Lane 4: Lane 4

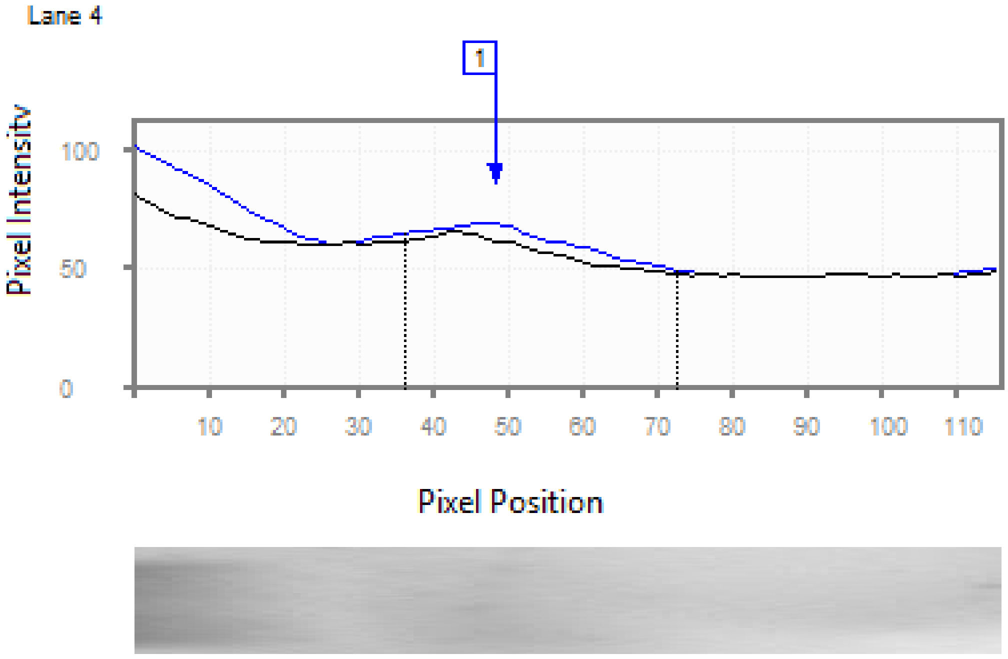

Method: Rolling Ball, Parameter: 20

Band Nr.	Band N.	Band Vol.	Backgr. Vol.	RF	MW
Band 1	1	15,542.000	199,281.000	0.417	--

Band Nr.	Cal. Band Vol.
Band 1	0.000

Lane 5: Lane 5

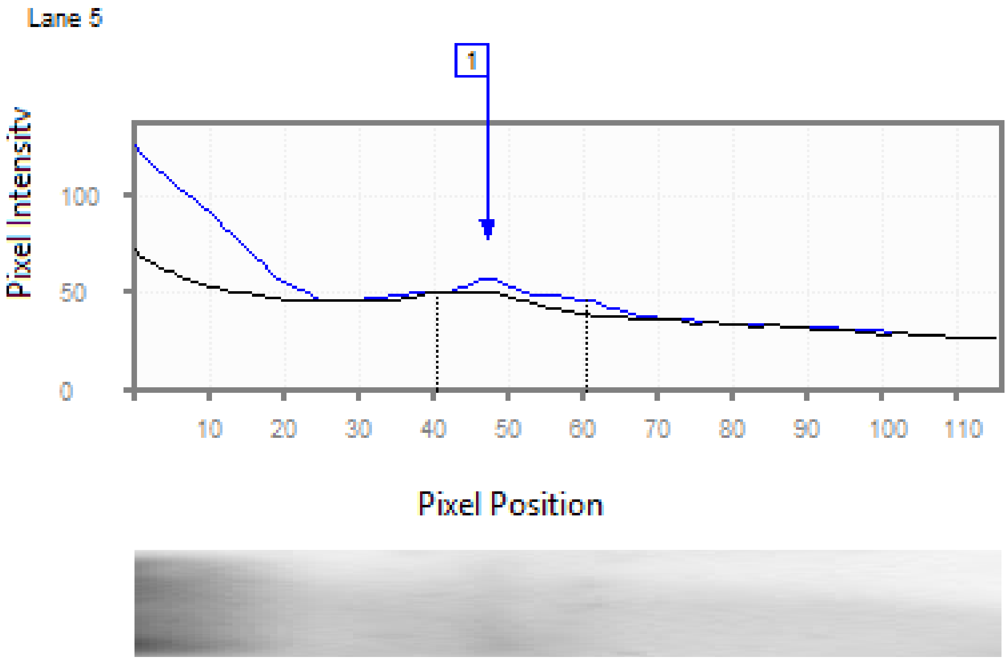

Method: Rolling Ball, Parameter: 20

Band Nr.	Band N.	Band Vol.	Backgr. Vol.	RF	MW
Band 1	1	9,665.000	89,819.000	0.409	--

Band Nr.	Cal. Band Vol.
Band 1	0.000

Lane 6: Lane 6

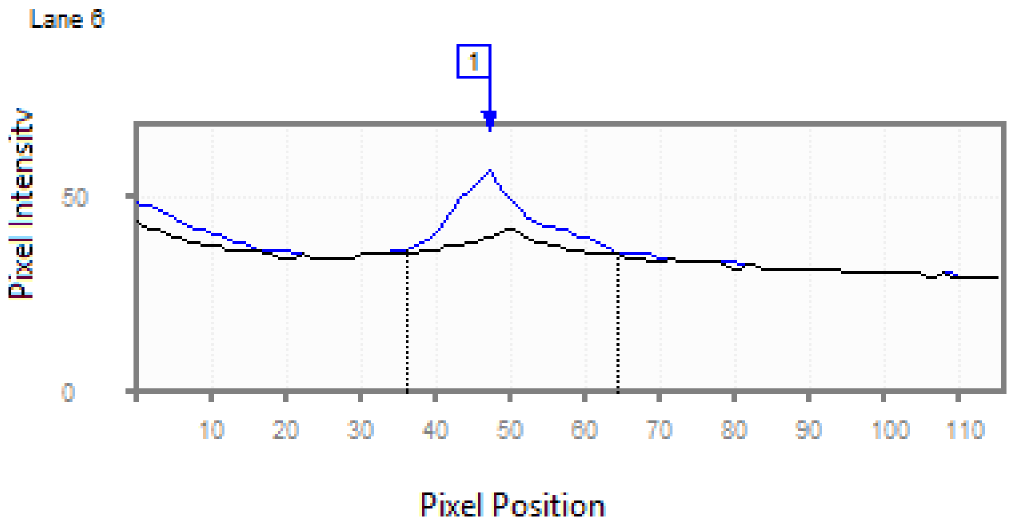

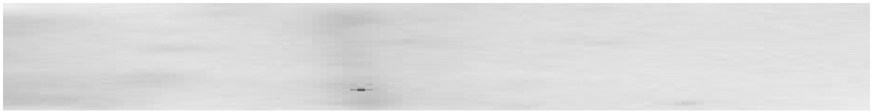

Method: Rolling Ball, Parameter: 20

Band Nr.	Band N.	Band Vol.	Backgr. Vol.	RF	MW
Band 1	1	20,260.000	116,725.000	0.409	--

Band Nr.	Cal. Band Vol.
Band 1	0.000
