# Supplementary material for: YB-1 Mediates TNF-Induced Pro-Survival Signaling by Regulating NF-κB Activation
Source: Cancers (Basel). 2020 Aug 5;12(8):2188. doi: 10.3390/cancers12082188 (PMC7464034; doi:10.3390/cancers12082188)
Supplement: Supplementary file 1 [file cancers-12-02188-s001.zip › Figure S4 Western blots/U937/Quantification/pp65.pdf]

Single Lane Report with Profile Project pp65 1

Project Data:

Name:	pp65 1
Project Status:	private
User:	anshah
Date:	26.05.2020, 13:07
Created at:	26.05.2020, 13:07
Type of Project:	Protein Gel
Comment:	No Arguments

Gel Image:

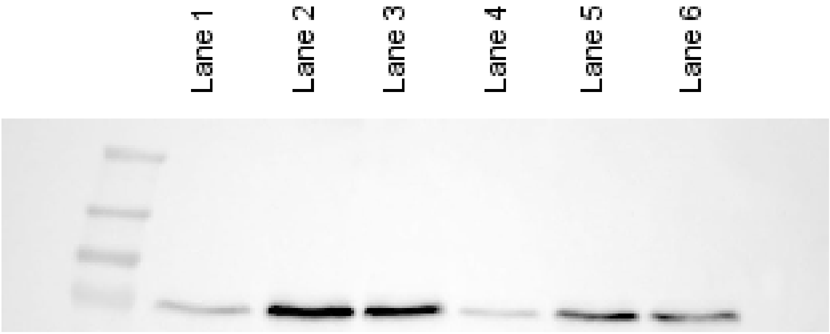

Lane 1: Lane 1

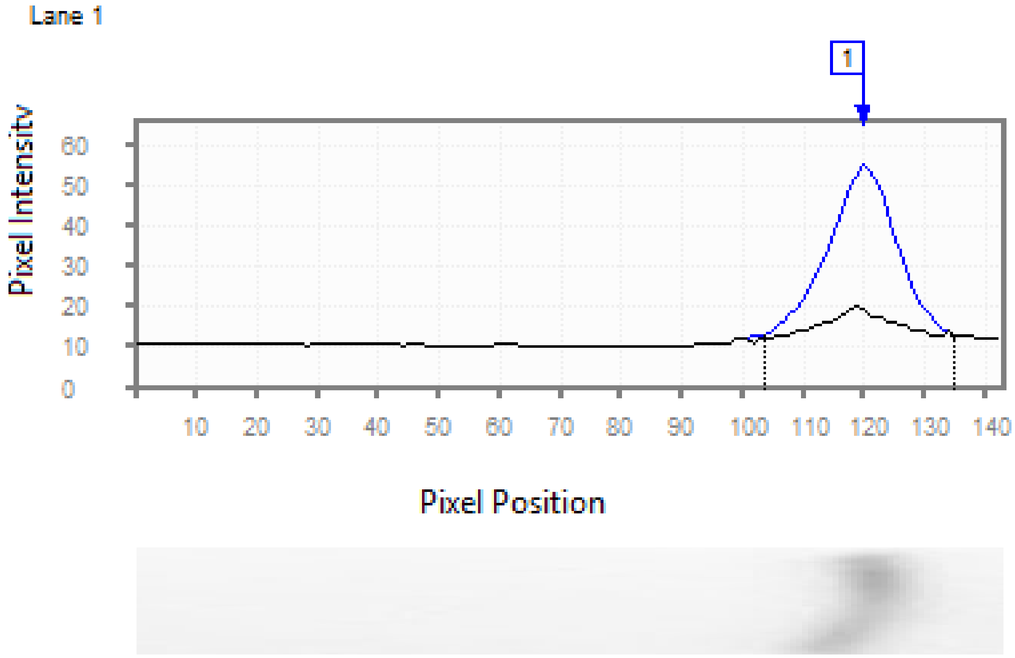

Method: Rolling Ball, Parameter: 20

Band Nr.	Band N.	Band Vol.	Backgr. Vol.	RF	MW
Band 1	1	57,284.000	58,033.000	0.838	--

Band Nr.	Cal. Band Vol.
Band 1	0.000

Lane 2: Lane 2

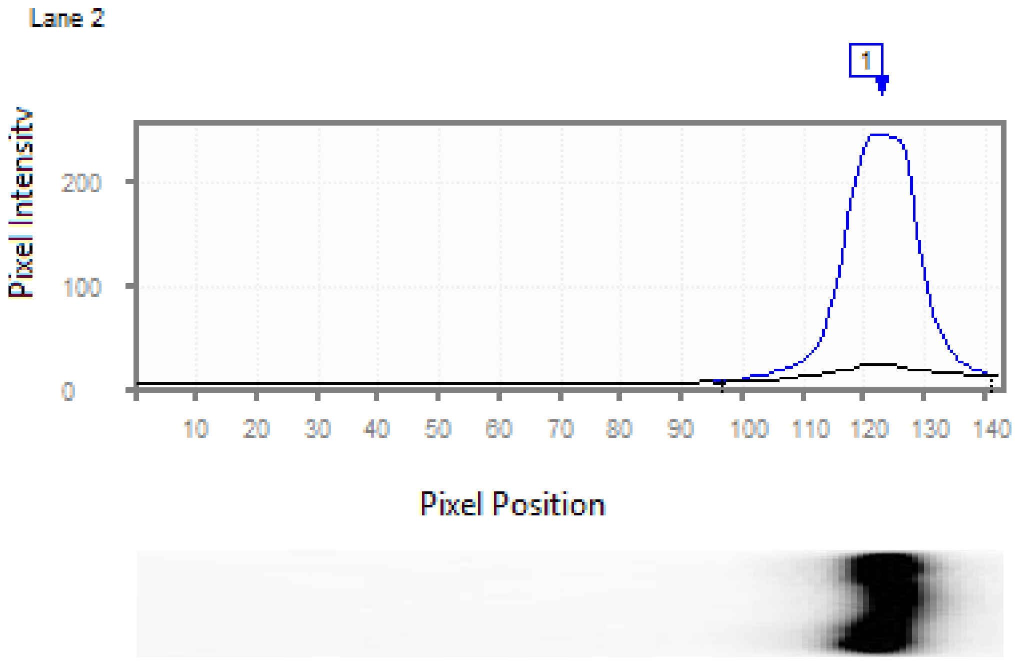

Method: Rolling Ball, Parameter: 20

Band Nr.	Band N.	Band Vol.	Backgr. Vol.	RF	MW
Band 1	1	365,154.000	79,456.000	0.859	--

Band Nr.	Cal. Band Vol.
Band 1	0.000

Lane 3: Lane 3

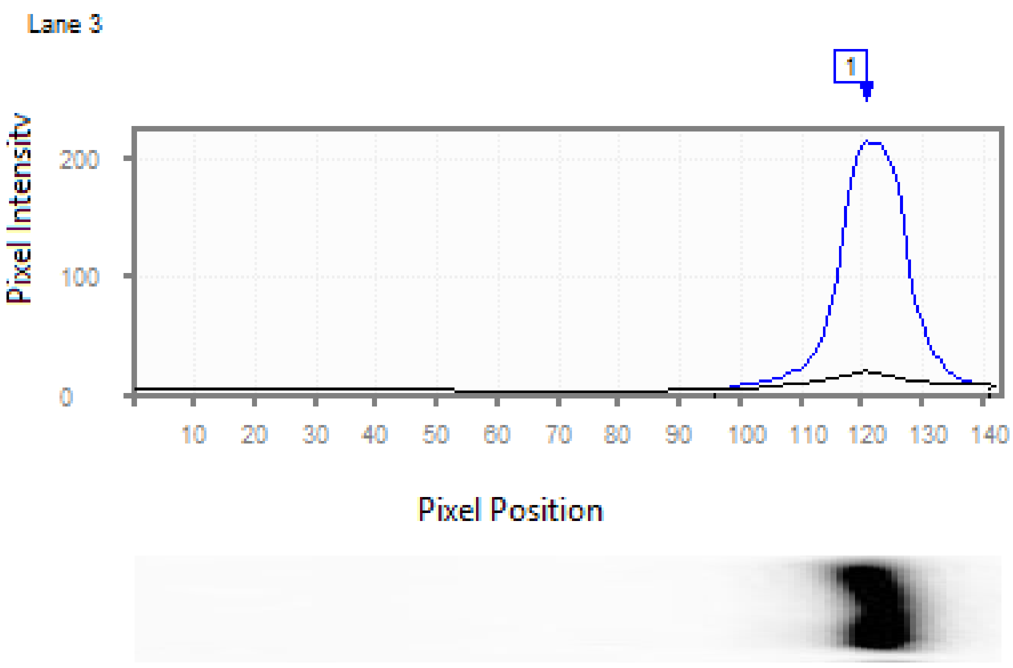

Method: Rolling Ball, Parameter: 20

Band Nr.	Band N.	Band Vol.	Backgr. Vol.	RF	MW
Band 1	1	288,415.000	59,450.000	0.845	--

Band Nr.	Cal. Band Vol.
Band 1	0.000

Lane 4: Lane 4

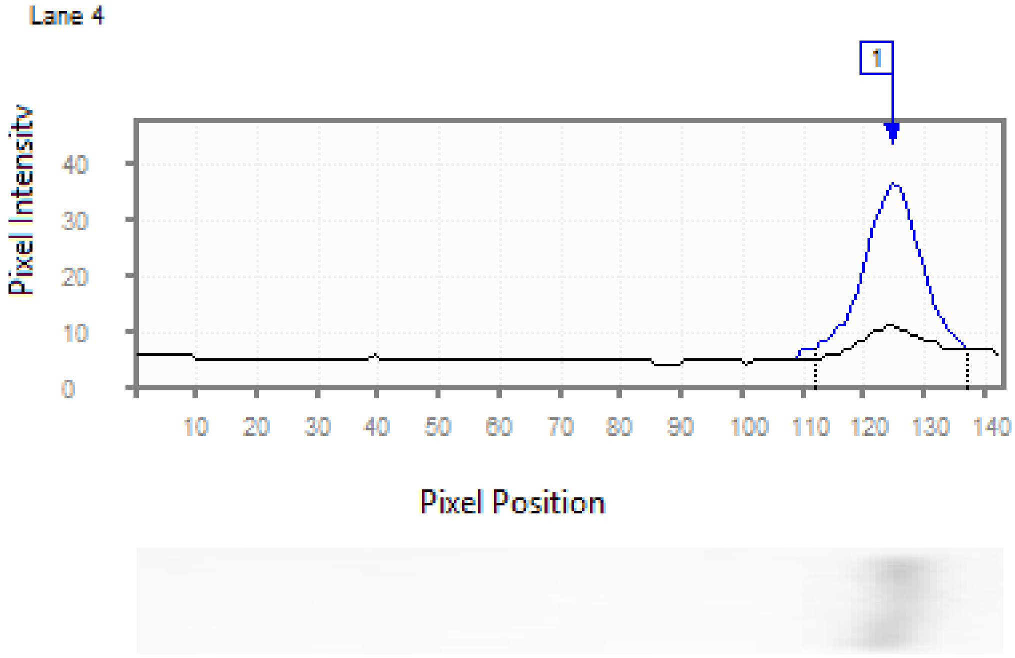

Method: Rolling Ball, Parameter: 20

Band Nr.	Band N.	Band Vol.	Backgr. Vol.	RF	MW
Band 1	1	31,317.000	23,605.000	0.873	--

Band Nr.	Cal. Band Vol.
Band 1	0.000

Lane 5: Lane 5

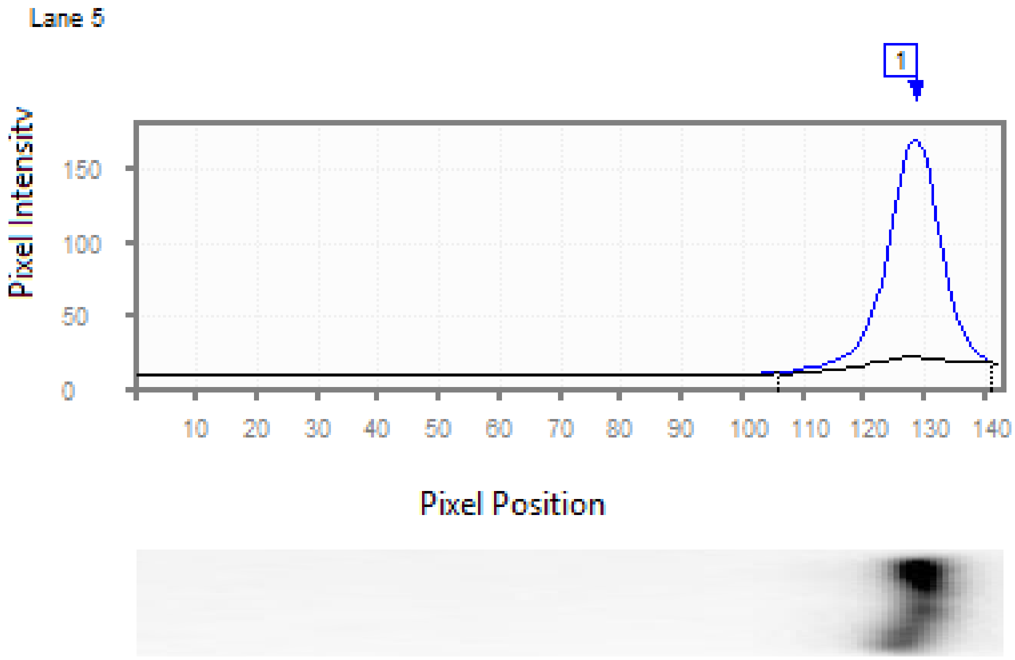

Method: Rolling Ball, Parameter: 20

Band Nr.	Band N.	Band Vol.	Backgr. Vol.	RF	MW
Band 1	1	173,337.000	67,616.000	0.901	--

Band Nr.	Cal. Band Vol.
Band 1	0.000

Lane 6: Lane 6

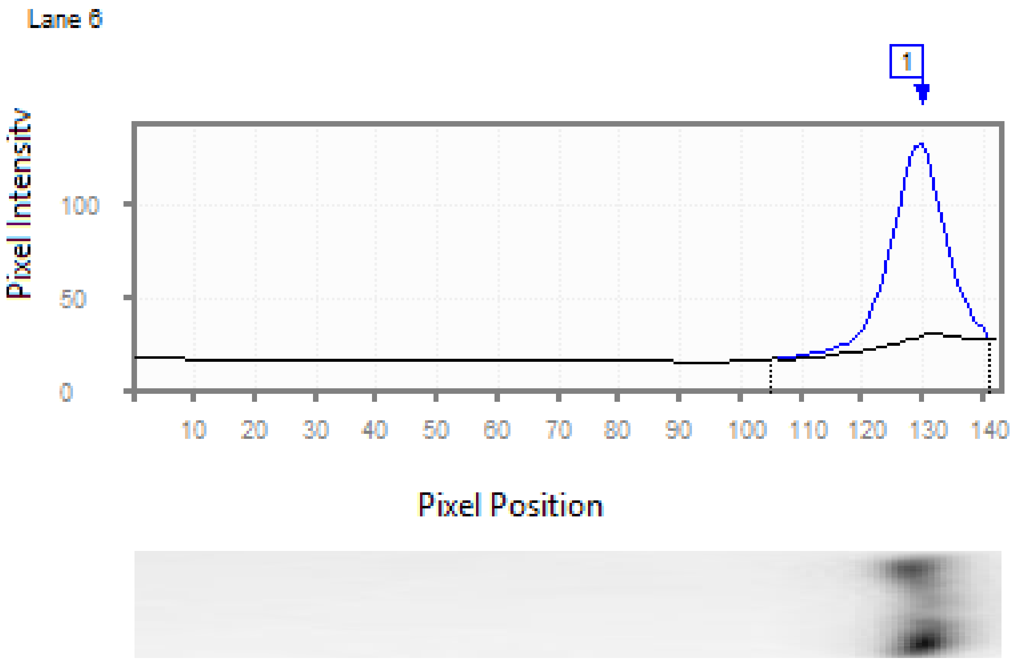

Method: Rolling Ball, Parameter: 20

Band Nr.	Band N.	Band Vol.	Backgr. Vol.	RF	MW
Band 1	1	123,023.000	95,493.000	0.908	--

Band Nr.	Cal. Band Vol.
Band 1	0.000
