# Supplementary material for: YB-1 Mediates TNF-Induced Pro-Survival Signaling by Regulating NF-κB Activation
Source: Cancers (Basel). 2020 Aug 5;12(8):2188. doi: 10.3390/cancers12082188 (PMC7464034; doi:10.3390/cancers12082188)
Supplement: Supplementary file 1 [file cancers-12-02188-s001.zip › Figure S4 Western blots/U937/Quantification/RIP1.pdf]

Single Lane Report with Profile Project RIP1

Project Data:

Name:	RIP1
Project Status:	private
User:	anshah
Date:	26.05.2020, 13:18
Created at:	26.05.2020, 13:18
Type of Project:	Protein Gel
Comment:	No Arguments

Gel Image:

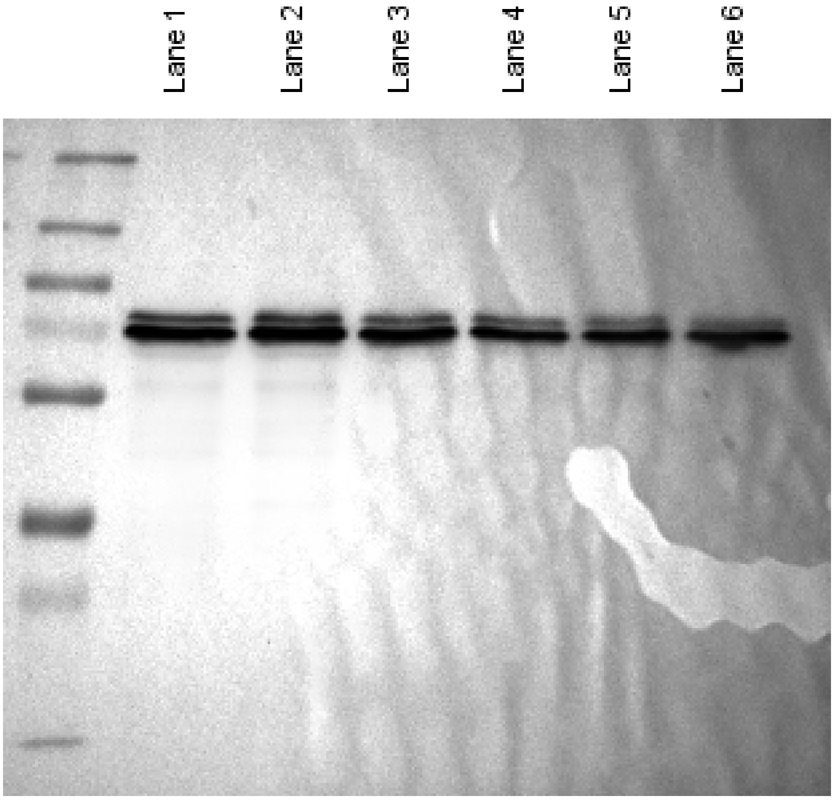

Lane 1: Lane 1

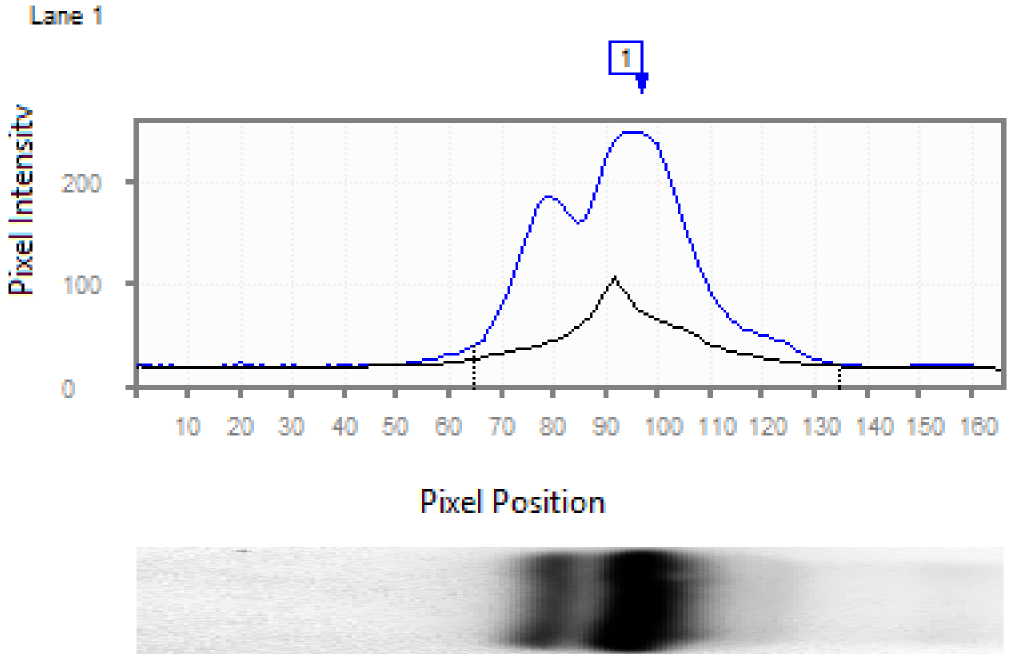

Method: Rolling Ball, Parameter: 20

Band Nr.	Band N.	Band Vol.	Backgr. Vol.	RF	MW
Band 1	1	712,221.000	425,769.000	0.582	--

Band Nr.	Cal. Band Vol.
Band 1	0.000

Lane 2: Lane 2

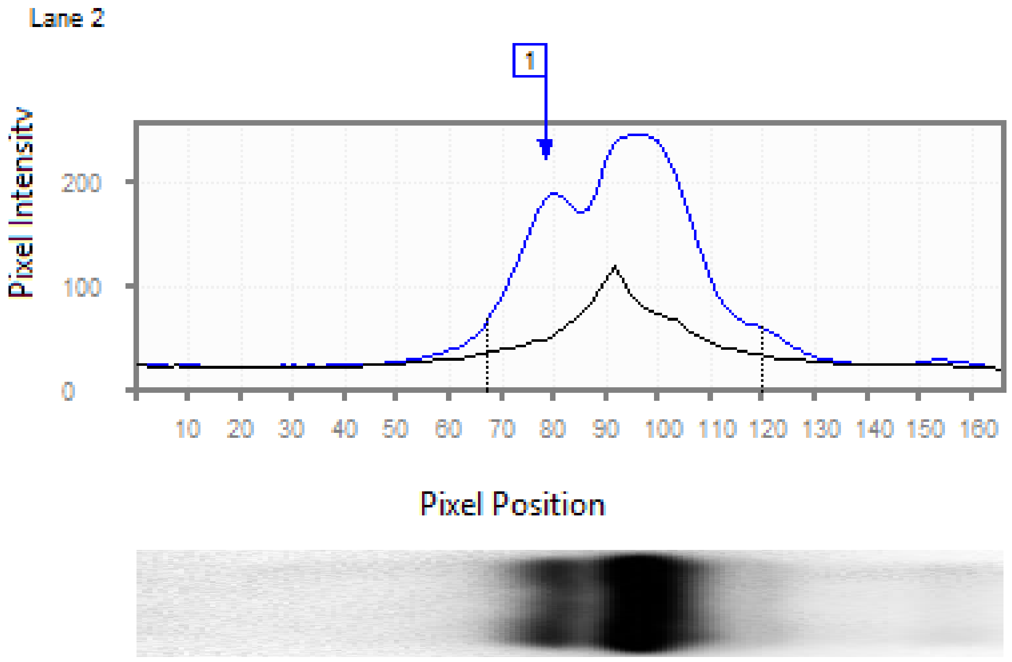

Method: Rolling Ball, Parameter: 20

Band Nr.	Band N.	Band Vol.	Backgr. Vol.	RF	MW
Band 1	1	615,085.000	376,649.000	0.473	--

Band Nr.	Cal. Band Vol.
Band 1	0.000

Lane 3: Lane 3

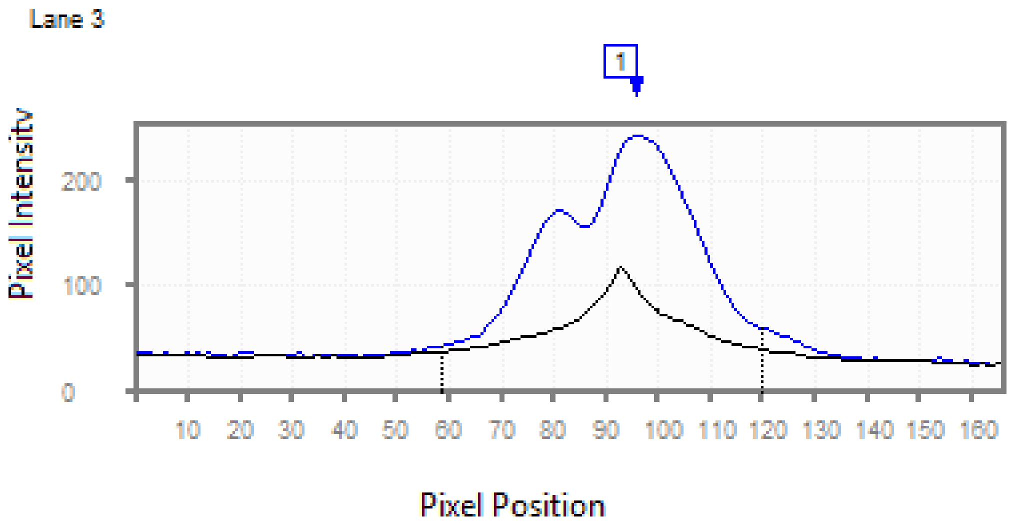

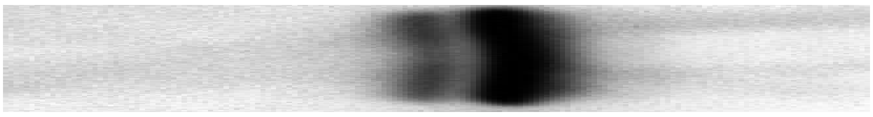

Method: Rolling Ball, Parameter: 20

Band Nr.	Band N.	Band Vol.	Backgr. Vol.	RF	MW
Band 1	1	548,464.000	441,822.000	0.576	--

Band Nr.	Cal. Band Vol.
Band 1	0.000

Lane 4: Lane 4

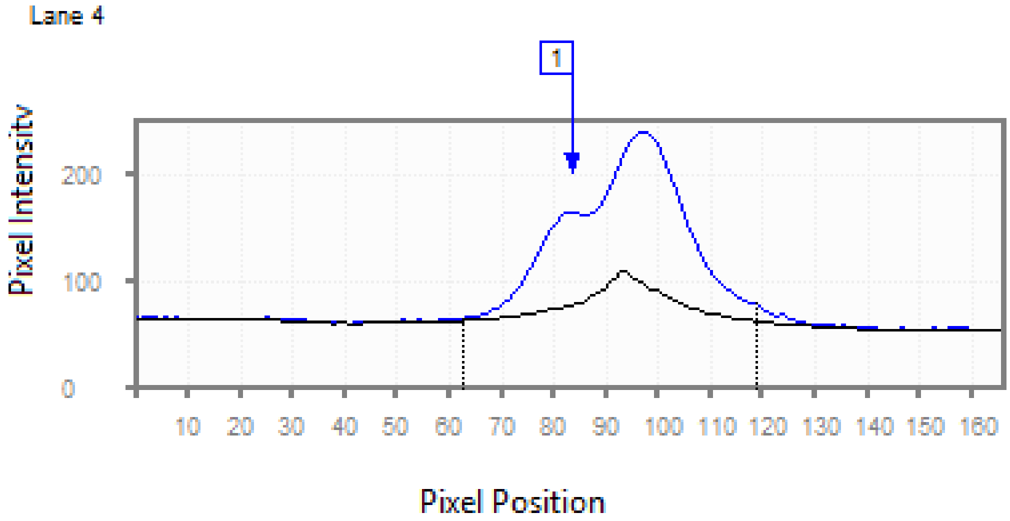

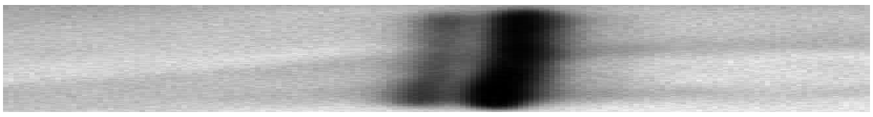

Method: Rolling Ball, Parameter: 20

Band Nr.	Band N.	Band Vol.	Backgr. Vol.	RF	MW
Band 1	1	419,827.000	510,780.000	0.503	--

Band Nr.	Cal. Band Vol.
Band 1	0.000

Lane 5: Lane 5

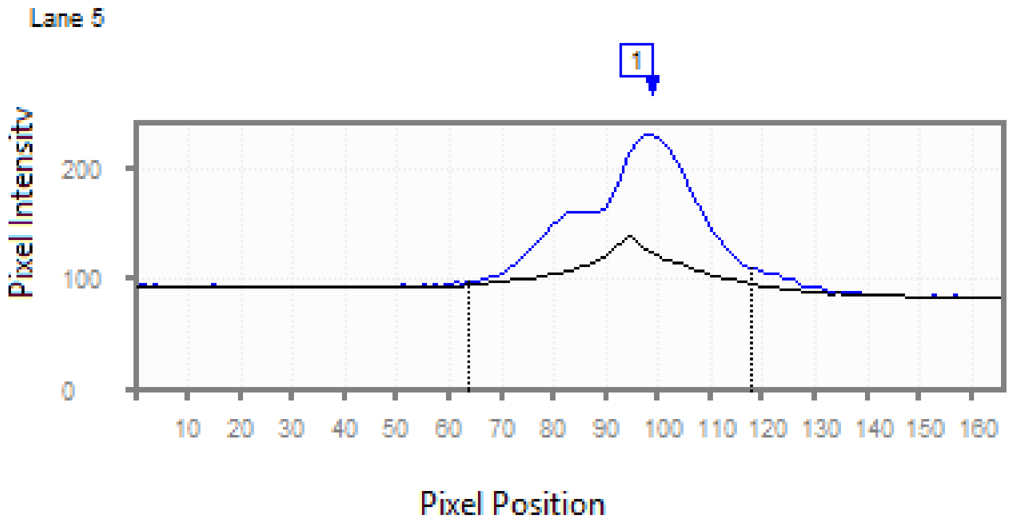

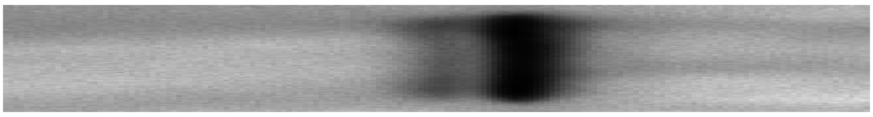

Method: Rolling Ball, Parameter: 20

Band Nr.	Band N.	Band Vol.	Backgr. Vol.	RF	MW
Band 1	1	299,959.000	701,453.000	0.594	--

Band Nr.	Cal. Band Vol.
Band 1	0.000

Lane 6: Lane 6

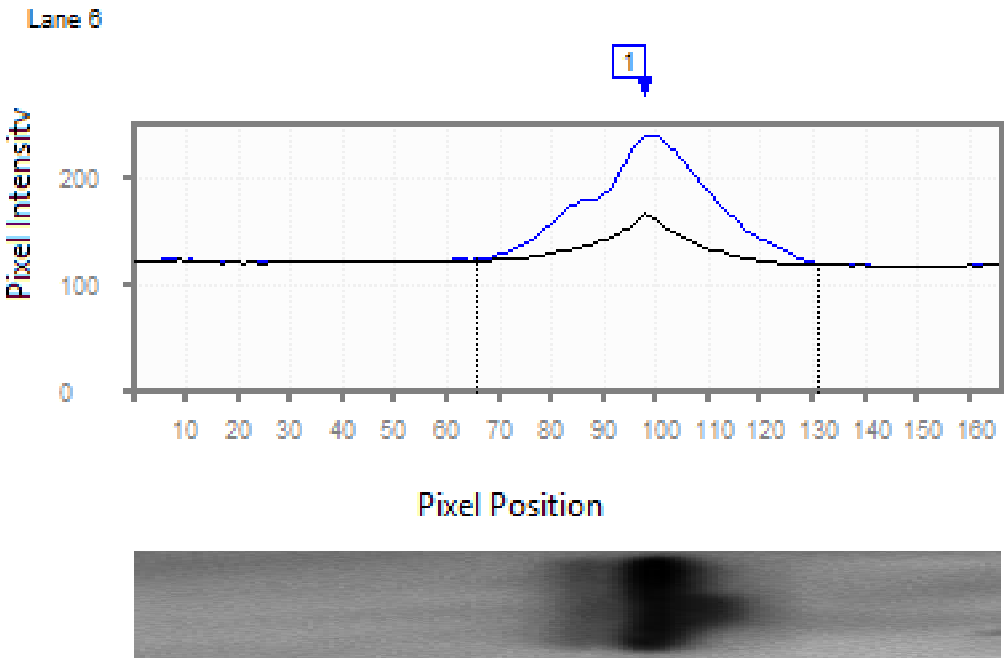

Method: Rolling Ball, Parameter: 20

Band Nr.	Band N.	Band Vol.	Backgr. Vol.	RF	MW
Band 1	1	295,702.000	1,109,450.000	0.588	--

Band Nr.	Cal. Band Vol.
Band 1	0.000
