# Supplementary material for: YB-1 Mediates TNF-Induced Pro-Survival Signaling by Regulating NF-κB Activation
Source: Cancers (Basel). 2020 Aug 5;12(8):2188. doi: 10.3390/cancers12082188 (PMC7464034; doi:10.3390/cancers12082188)
Supplement: Supplementary file 1 [file cancers-12-02188-s001.zip › Figure S4 Western blots/U937/Quantification/Vinculin.pdf]

Single Lane Report with Profile Project Vinculin

Project Data:

Name:	Vinculin
Project Status:	private
User:	anshah
Date:	26.05.2020, 13:01
Created at:	26.05.2020, 13:01
Type of Project:	Protein Gel
Comment:	No Arguments

Gel Image:

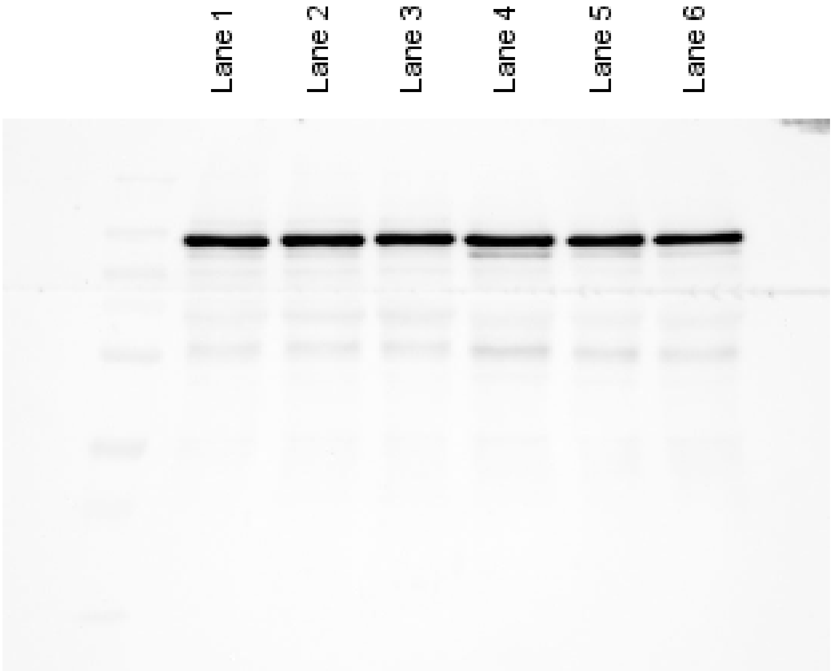

Lane 1: Lane 1

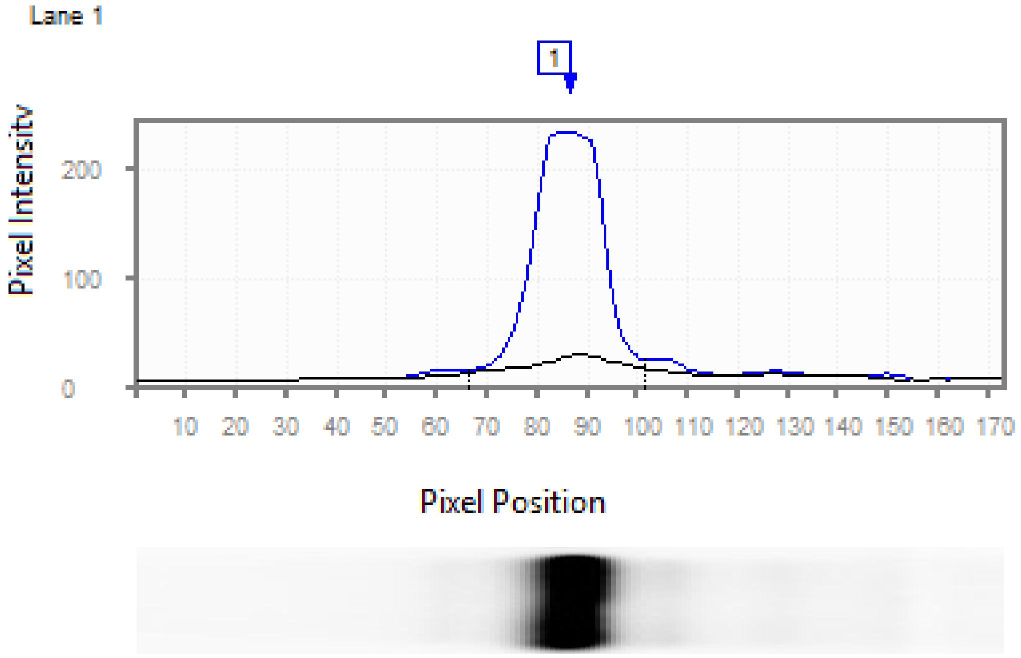

Method: Rolling Ball, Parameter: 20

Band Nr.	Band N.	Band Vol.	Backgr. Vol.	RF	MW
Band 1	1	411,956.000	94,383.000	0.500	--

Band Nr.	Cal. Band Vol.
Band 1	0.000

Lane 2: Lane 2

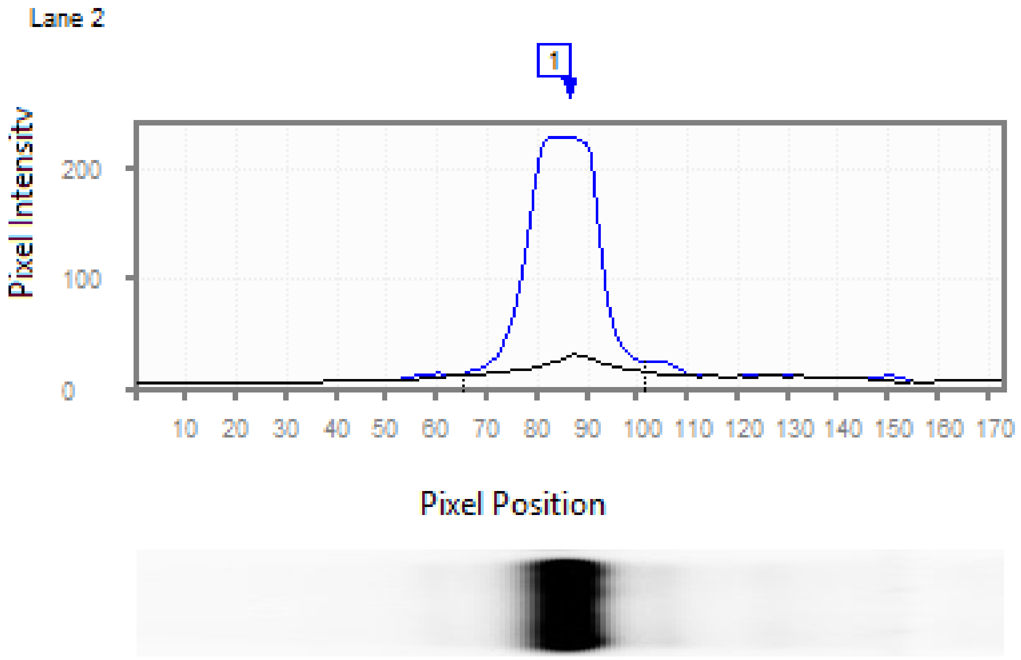

Method: Rolling Ball, Parameter: 20

Band Nr.	Band N.	Band Vol.	Backgr. Vol.	RF	MW
Band 1	1	412,378.000	93,198.000	0.500	--

Band Nr.	Cal. Band Vol.
Band 1	0.000

Lane 3: Lane 3

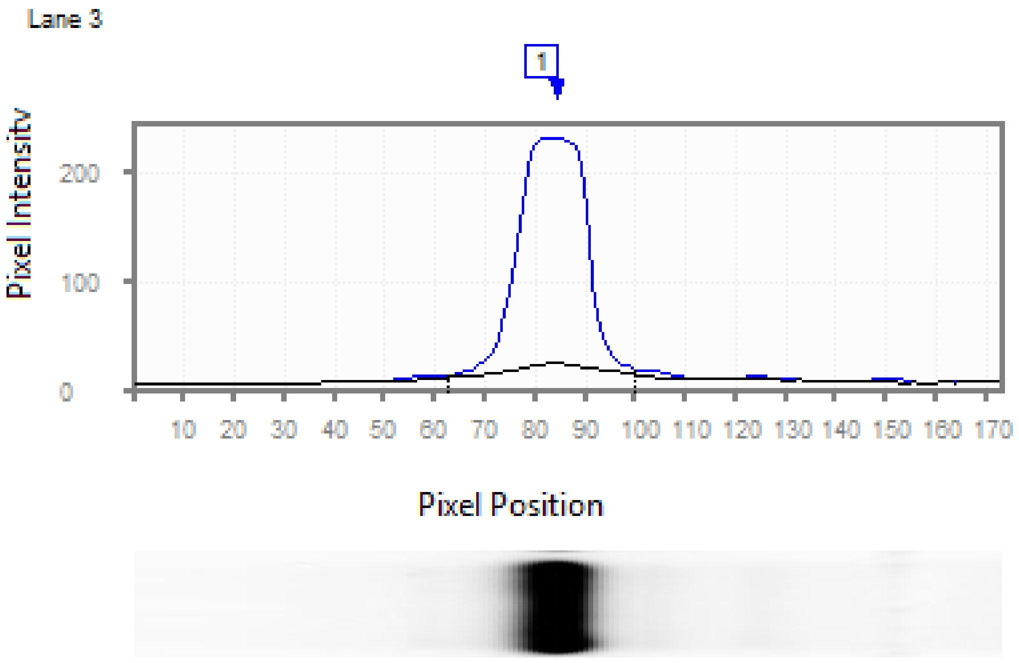

Method: Rolling Ball, Parameter: 20

Band Nr.	Band N.	Band Vol.	Backgr. Vol.	RF	MW
Band 1	1	385,222.000	83,262.000	0.488	--

Band Nr.	Cal. Band Vol.
Band 1	0.000

Lane 4: Lane 4

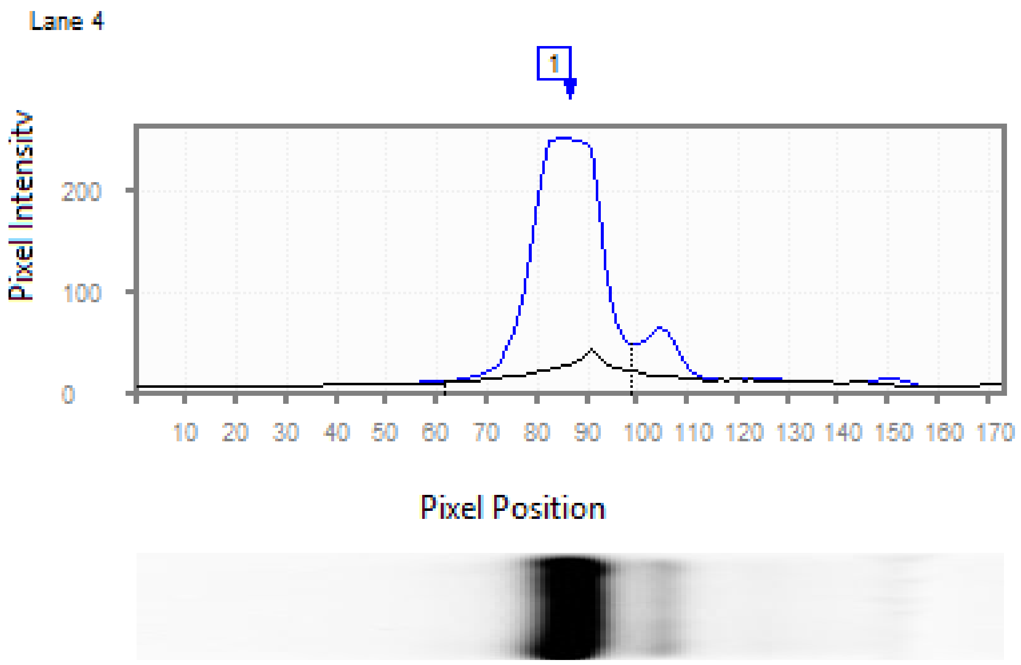

Method: Rolling Ball, Parameter: 20

Band Nr.	Band N.	Band Vol.	Backgr. Vol.	RF	MW
Band 1	1	420,962.000	95,169.000	0.500	--

Band Nr.	Cal. Band Vol.
Band 1	0.000

Lane 5: Lane 5

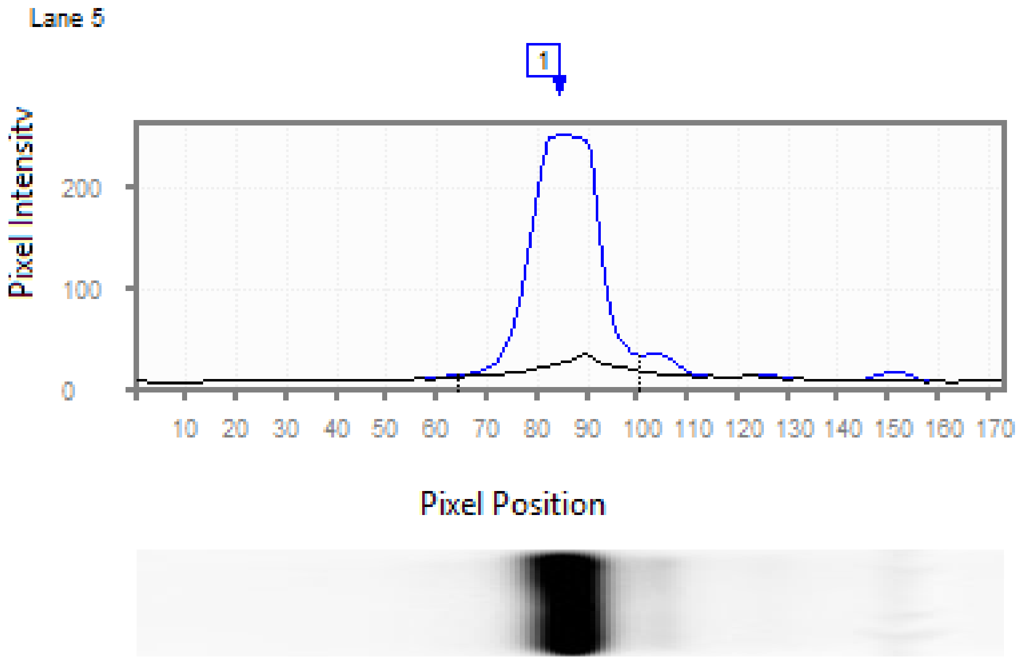

Method: Rolling Ball, Parameter: 20

Band Nr.	Band N.	Band Vol.	Backgr. Vol.	RF	MW
Band 1	1	368,087.000	79,674.000	0.488	--

Band Nr.	Cal. Band Vol.
Band 1	0.000

Lane 6: Lane 6

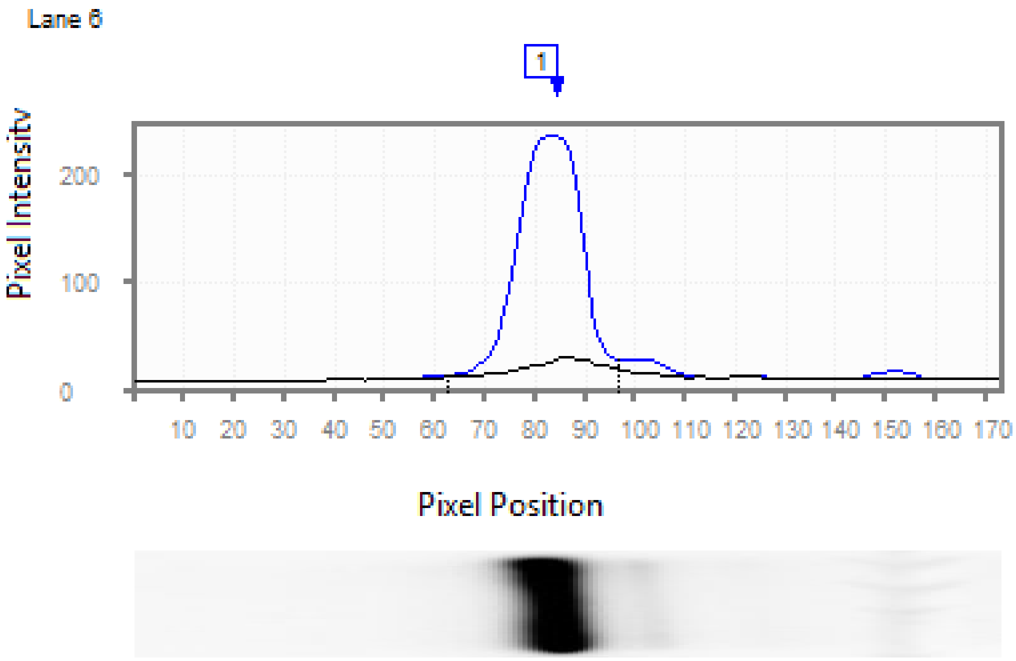

Method: Rolling Ball, Parameter: 20

Band Nr.	Band N.	Band Vol.	Backgr. Vol.	RF	MW
Band 1	1	384,615.000	92,062.000	0.488	--

Band Nr.	Cal. Band Vol.
Band 1	0.000
