# Supplementary figures and images for: YB-1 Mediates TNF-Induced Pro-Survival Signaling by Regulating NF-κB Activation
Source: Cancers (Basel). 2020 Aug 5;12(8):2188. doi: 10.3390/cancers12082188 (PMC7464034; doi:10.3390/cancers12082188)

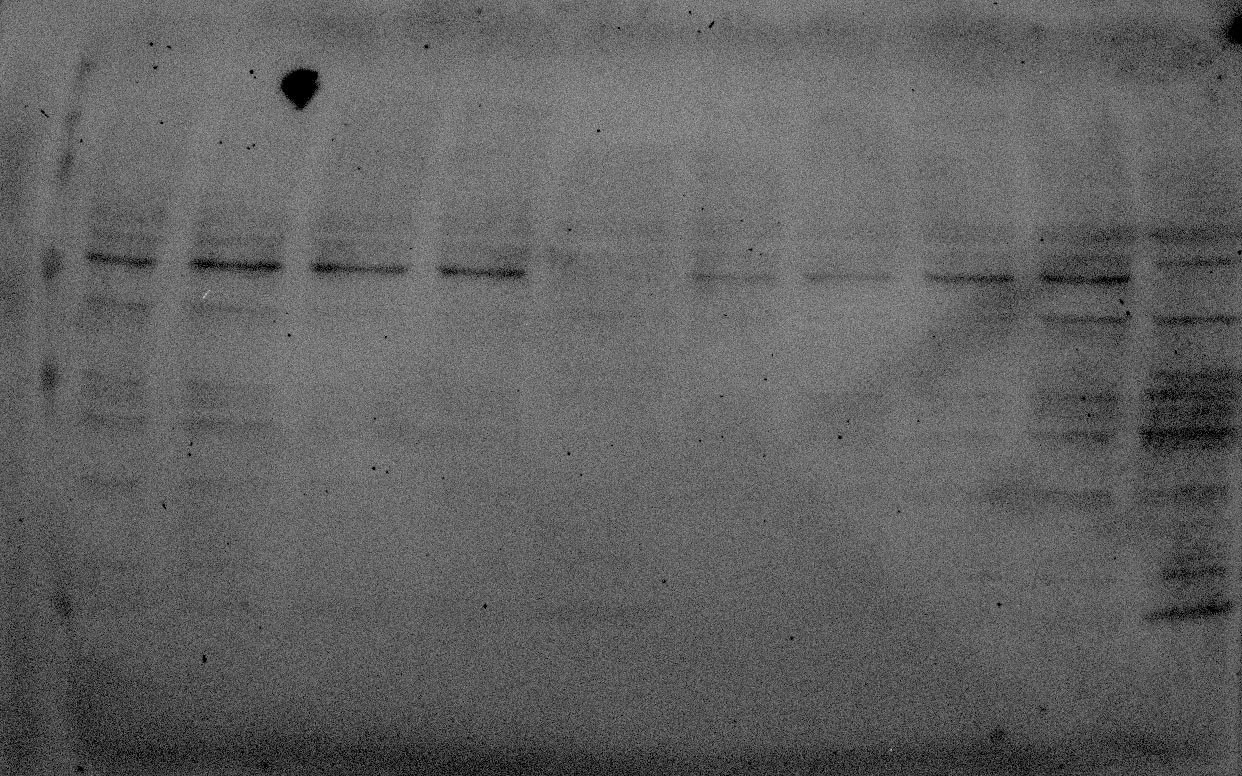

Supplement: Supplementary file 1 [file cancers-12-02188-s001.zip › Figure S4 Western blots/Apoptosis/THP1/JEPG/cleaved caspase 8.jpg]

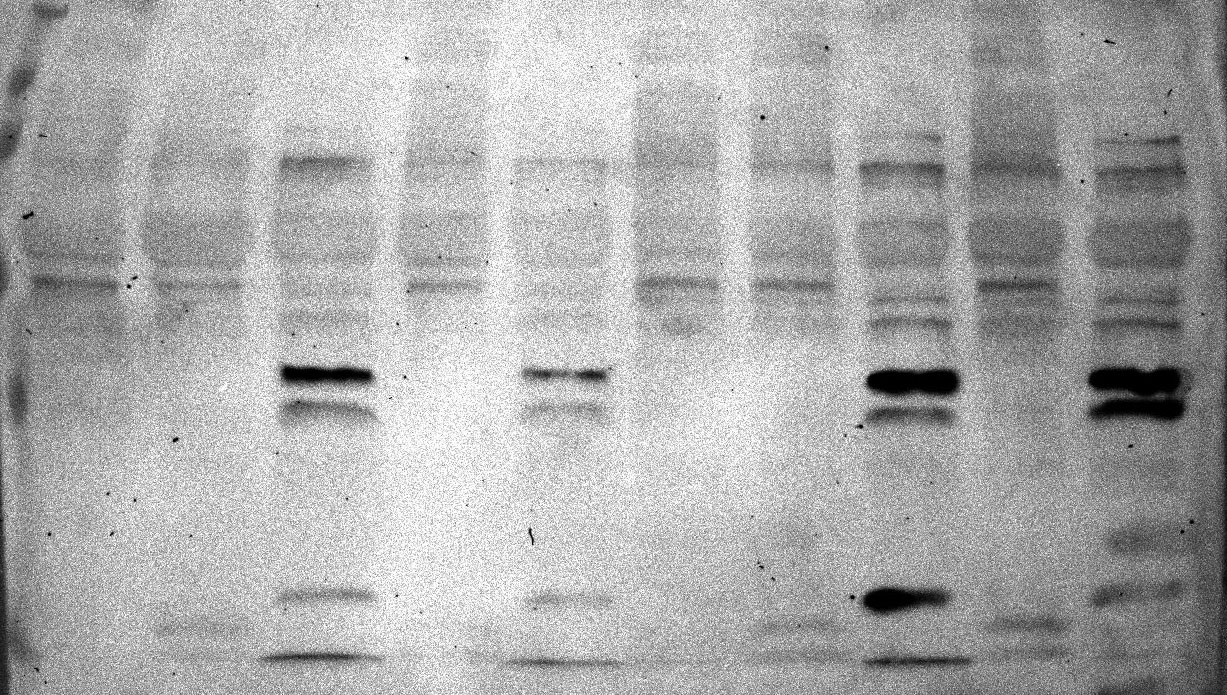

Supplement: Supplementary file 1 [file cancers-12-02188-s001.zip › Figure S4 Western blots/Apoptosis/THP1/JEPG/cleaved caspsae 3.jpg]

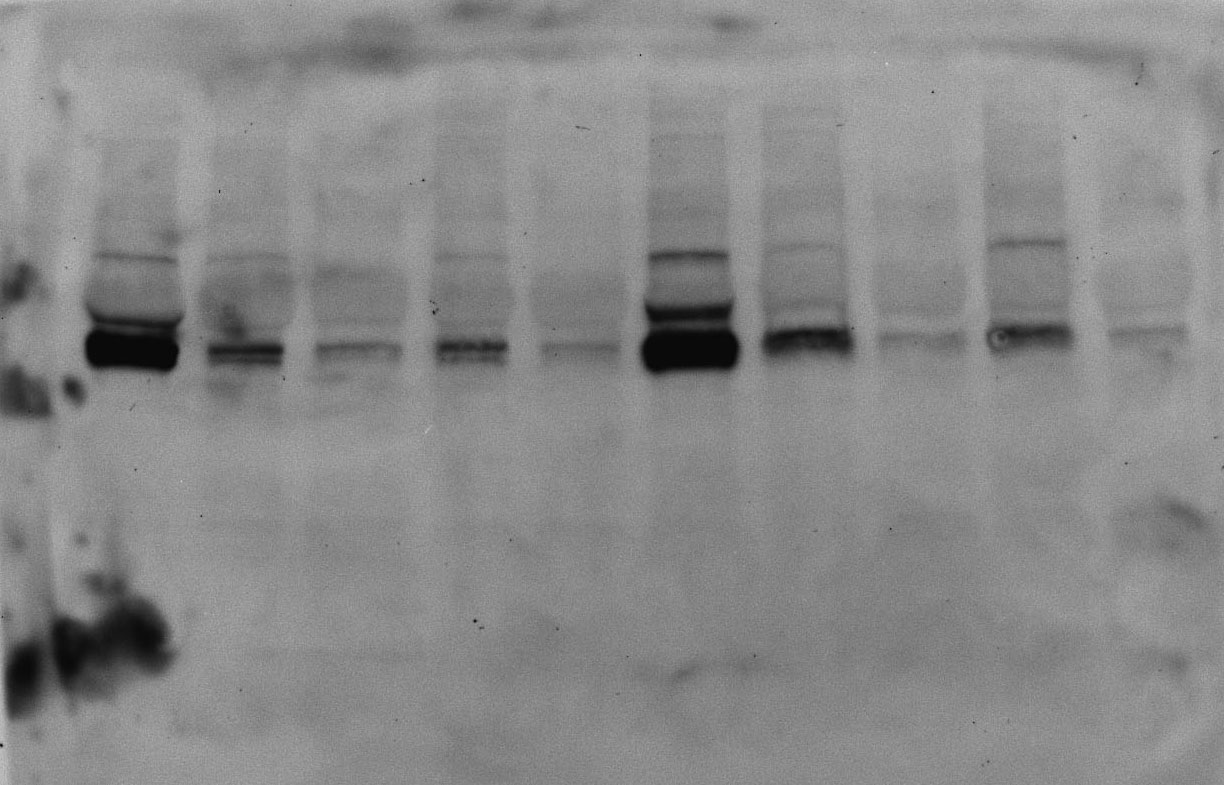

Supplement: Supplementary file 1 [file cancers-12-02188-s001.zip › Figure S4 Western blots/Apoptosis/THP1/JEPG/total caspase 8.jpg]

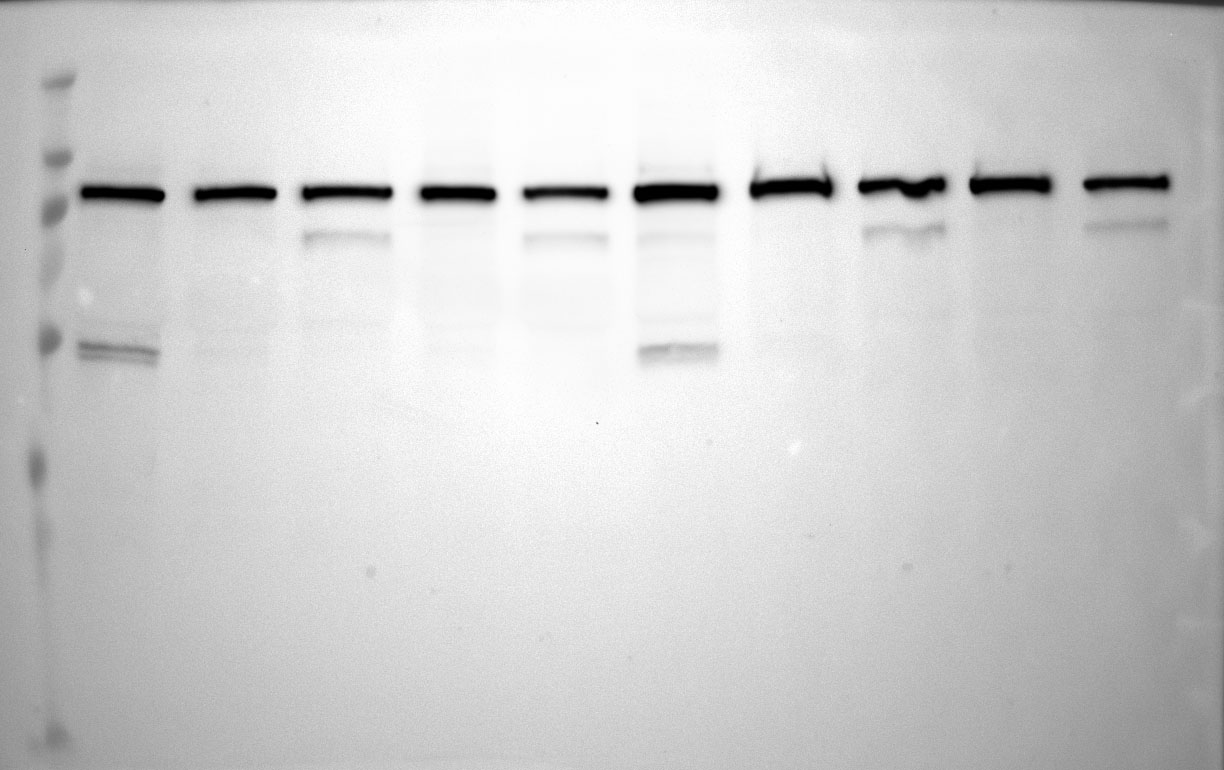

Supplement: Supplementary file 1 [file cancers-12-02188-s001.zip › Figure S4 Western blots/Apoptosis/THP1/JEPG/Vinculin.jpg]

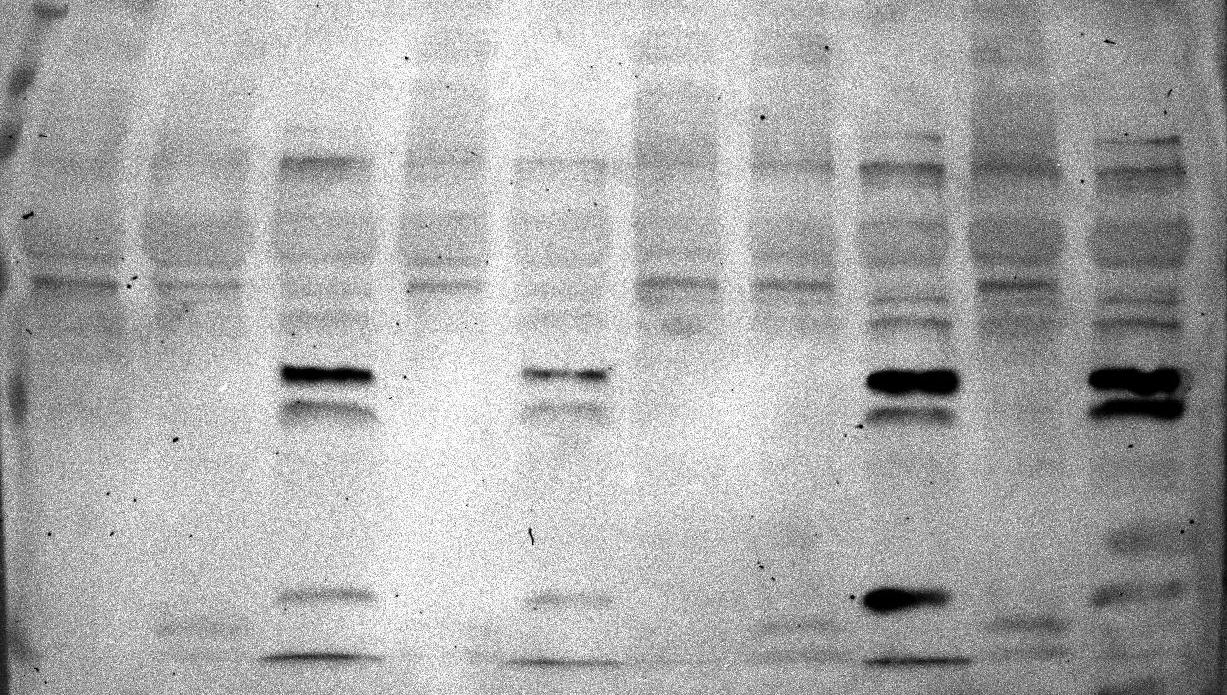

Supplement: Supplementary file 1 [file cancers-12-02188-s001.zip › Figure S4 Western blots/Apoptosis/THP1/TIFF/cleaved caspase 3.tif]

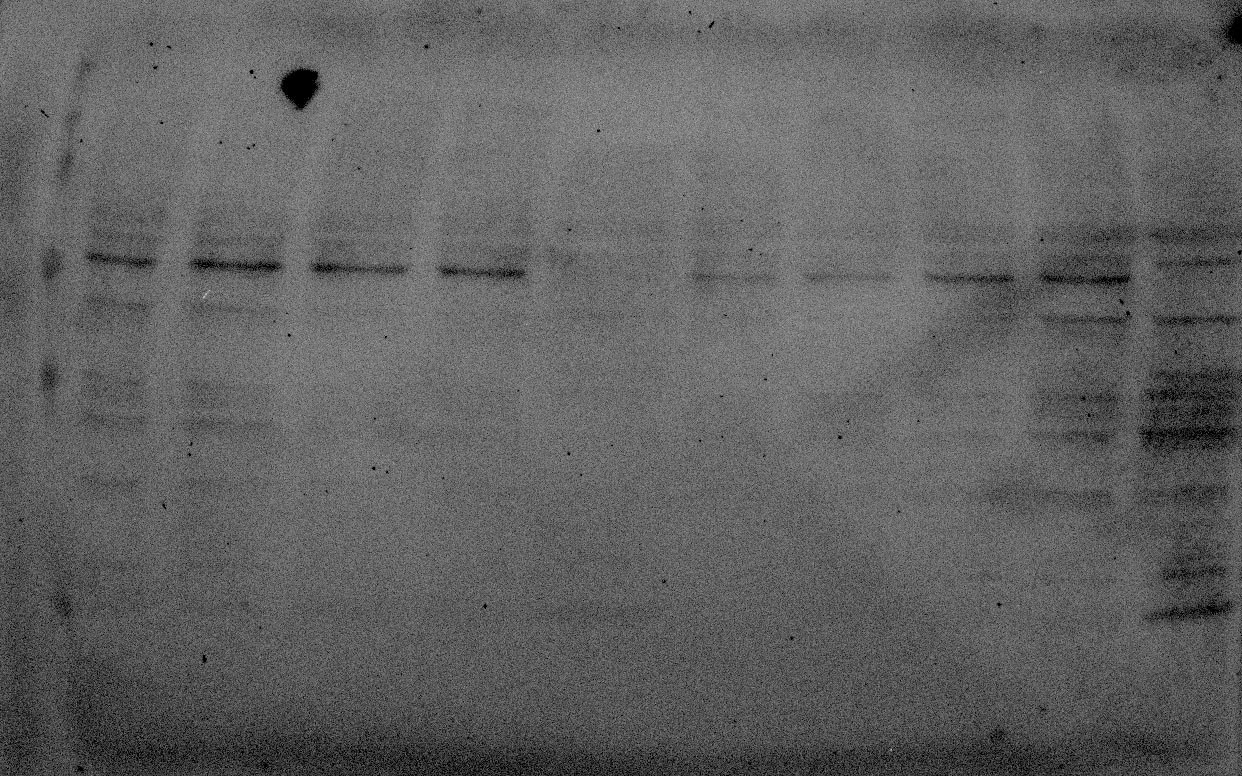

Supplement: Supplementary file 1 [file cancers-12-02188-s001.zip › Figure S4 Western blots/Apoptosis/THP1/TIFF/cleaved caspase 8.tif]

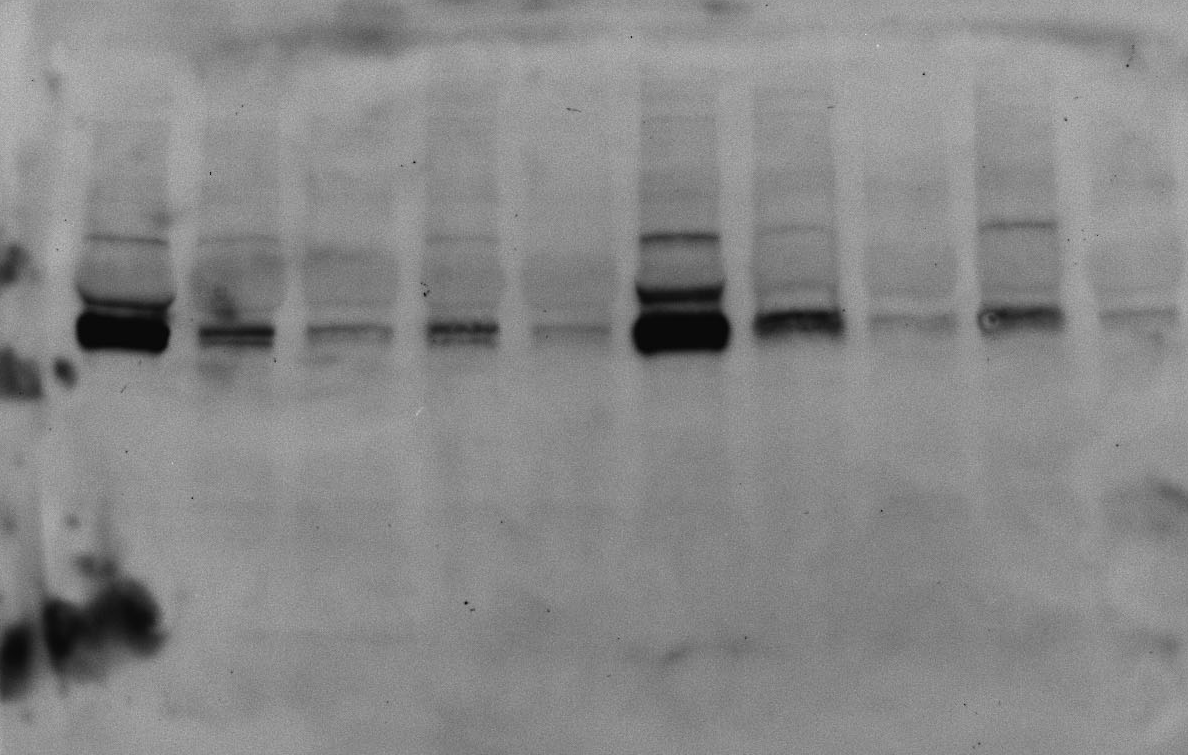

Supplement: Supplementary file 1 [file cancers-12-02188-s001.zip › Figure S4 Western blots/Apoptosis/THP1/TIFF/total caspase 8.tif]

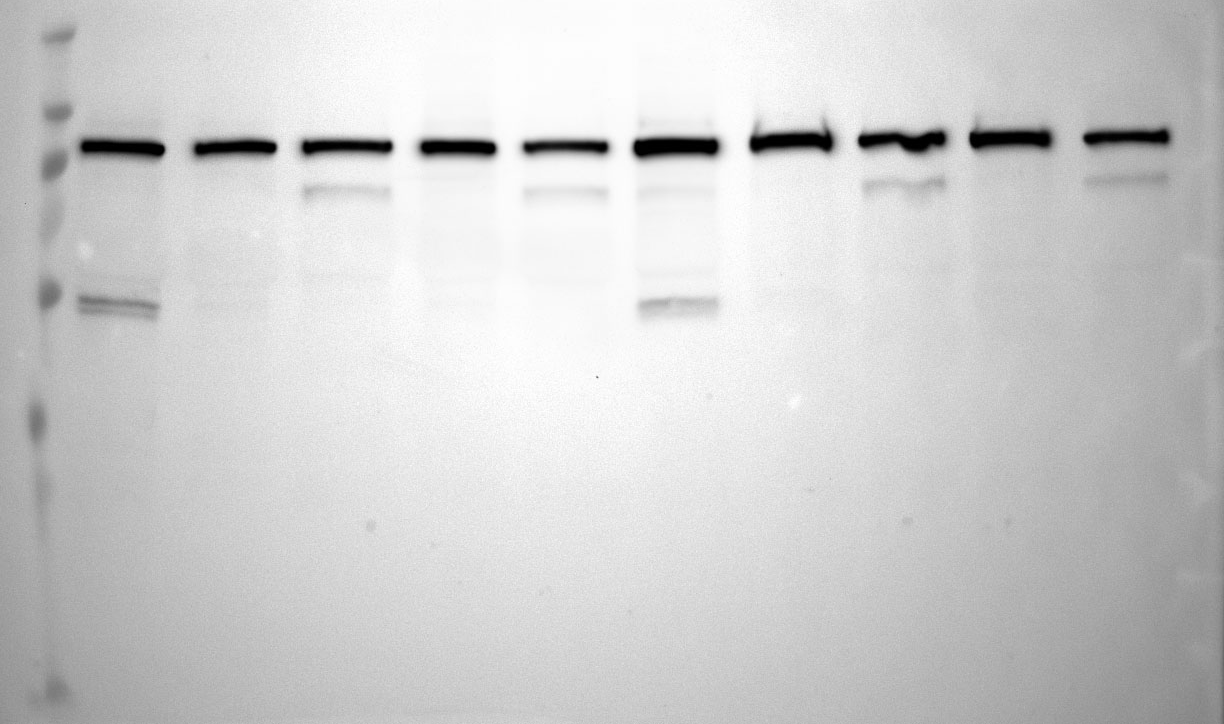

Supplement: Supplementary file 1 [file cancers-12-02188-s001.zip › Figure S4 Western blots/Apoptosis/THP1/TIFF/Vinculin.tif]

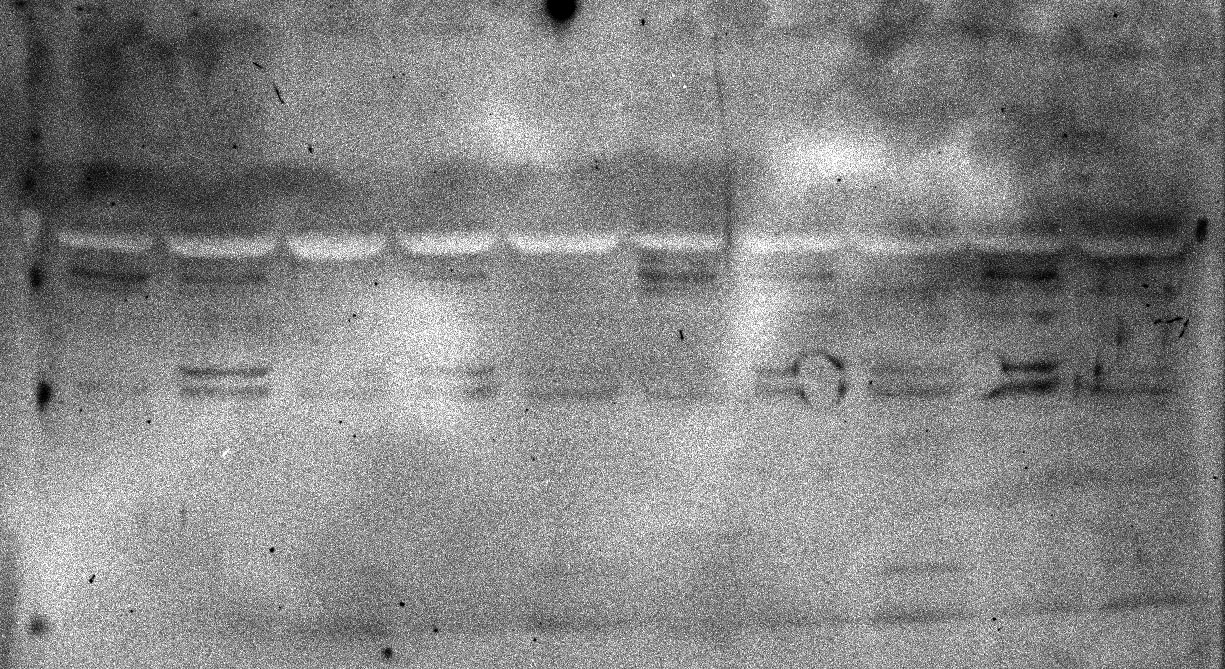

Supplement: Supplementary file 1 [file cancers-12-02188-s001.zip › Figure S4 Western blots/Apoptosis/U937/JEPG/cleaved caspase 8.jpg]

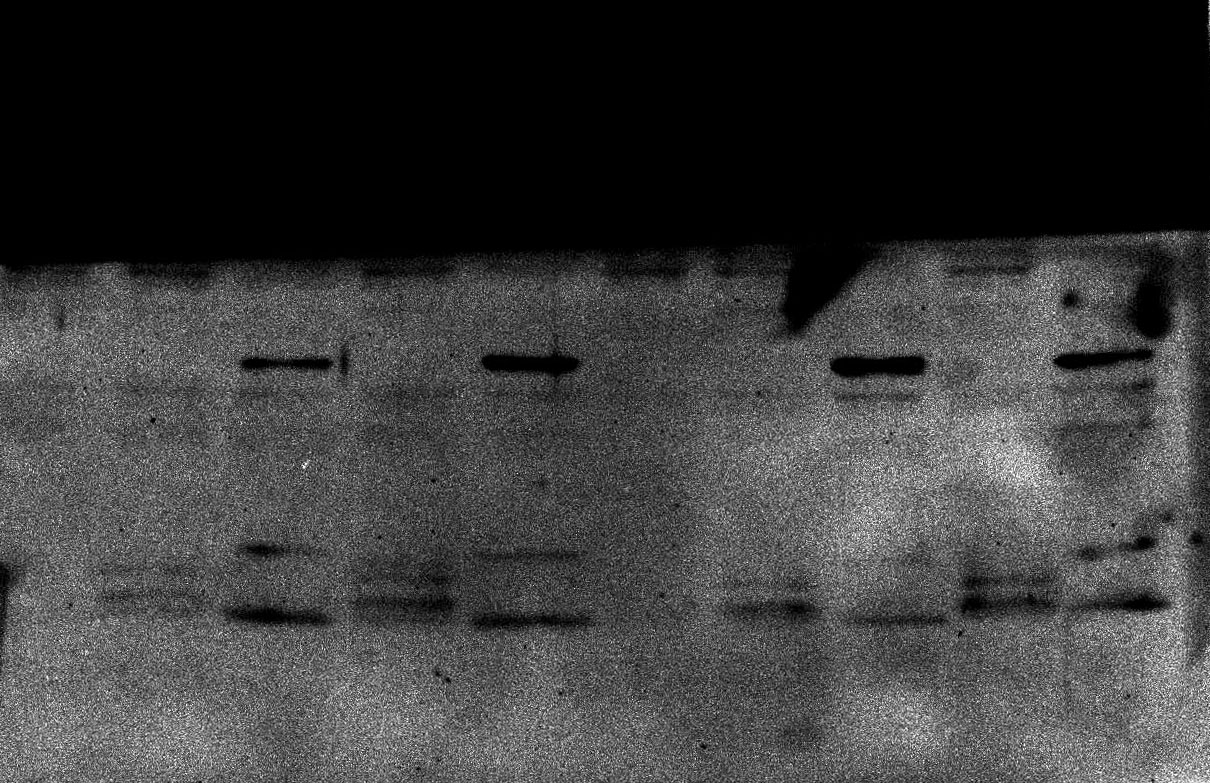

Supplement: Supplementary file 1 [file cancers-12-02188-s001.zip › Figure S4 Western blots/Apoptosis/U937/JEPG/cleaved caspsae 3.jpg]

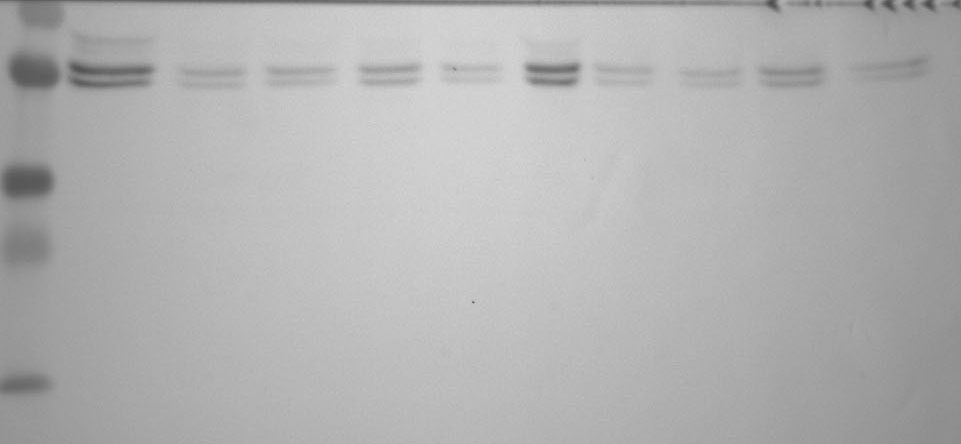

Supplement: Supplementary file 1 [file cancers-12-02188-s001.zip › Figure S4 Western blots/Apoptosis/U937/JEPG/total caspase 8.jpg]

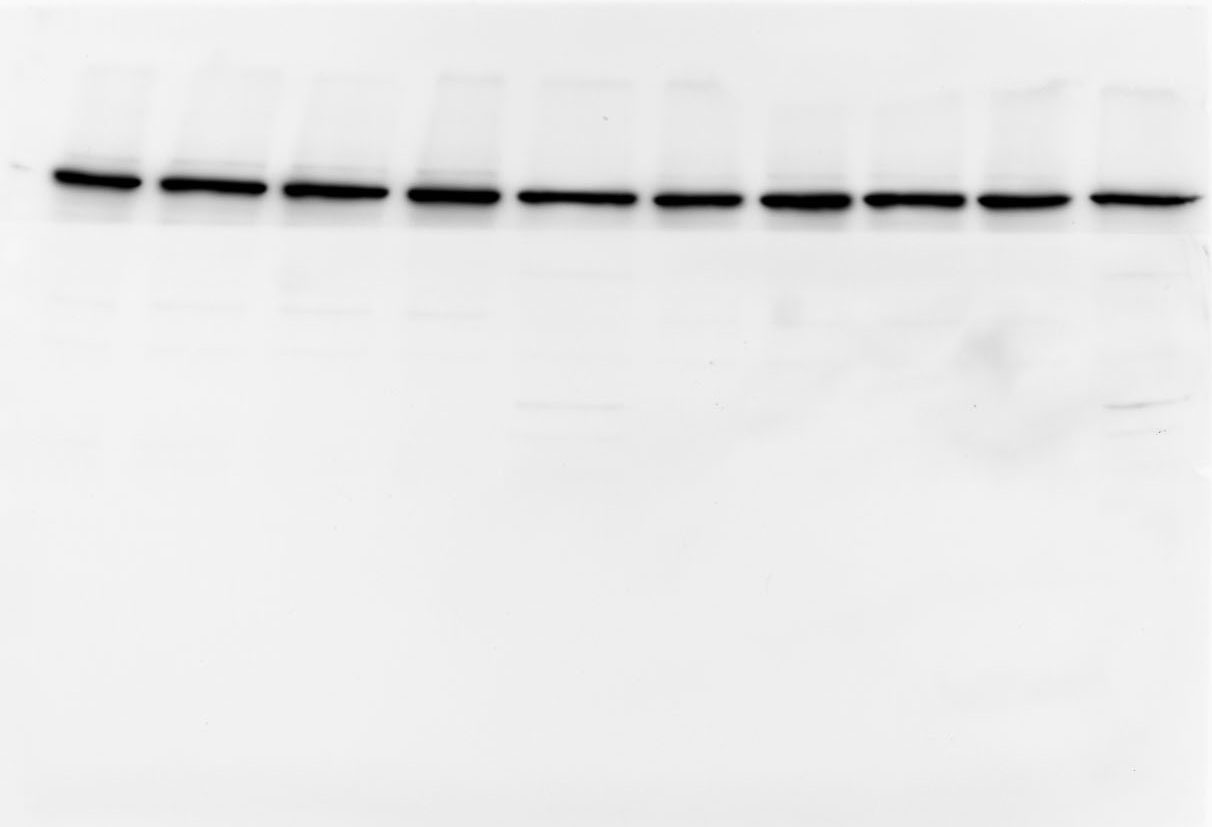

Supplement: Supplementary file 1 [file cancers-12-02188-s001.zip › Figure S4 Western blots/Apoptosis/U937/JEPG/Vinculin.jpg]

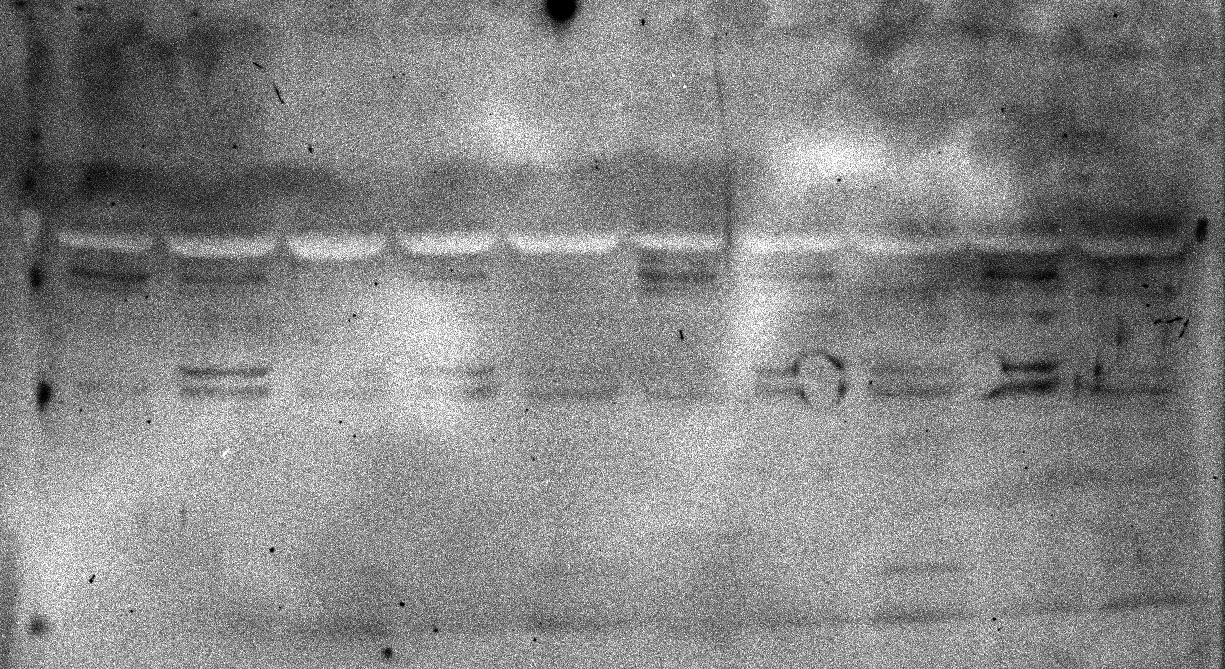

Supplement: Supplementary file 1 [file cancers-12-02188-s001.zip › Figure S4 Western blots/Apoptosis/U937/TIFF/cleaved caspase 8.tif]

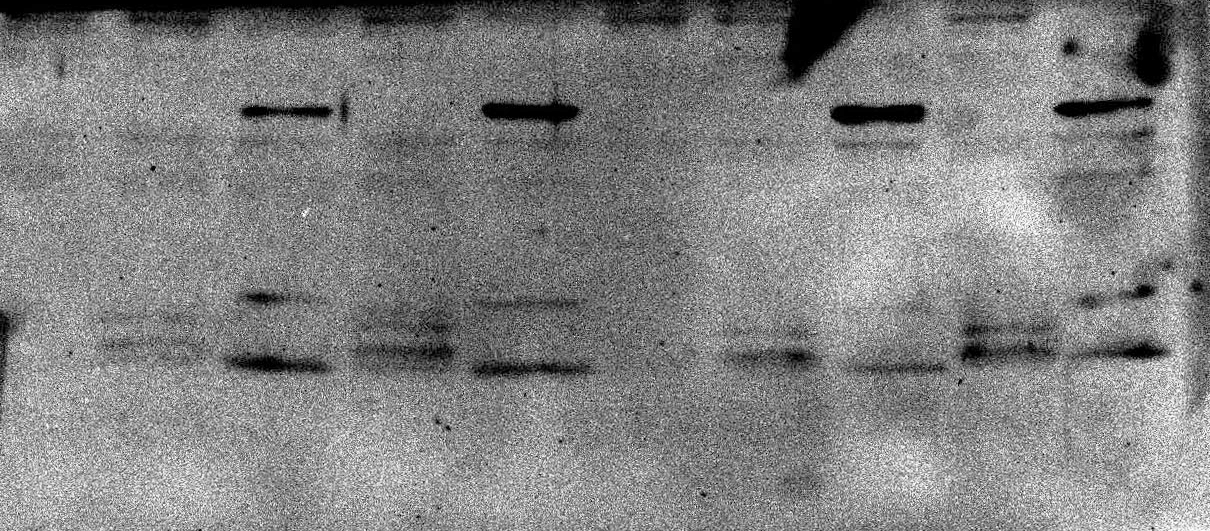

Supplement: Supplementary file 1 [file cancers-12-02188-s001.zip › Figure S4 Western blots/Apoptosis/U937/TIFF/cleaved caspsae 3.tif]

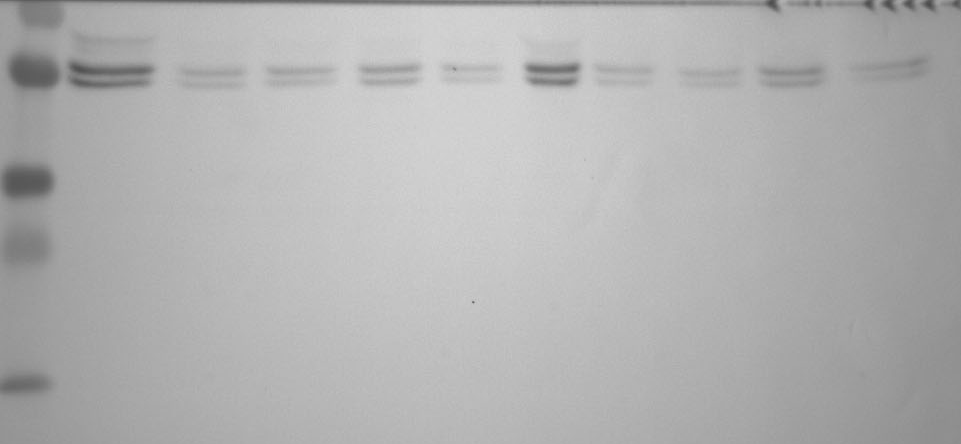

Supplement: Supplementary file 1 [file cancers-12-02188-s001.zip › Figure S4 Western blots/Apoptosis/U937/TIFF/total caspase 8.tif]

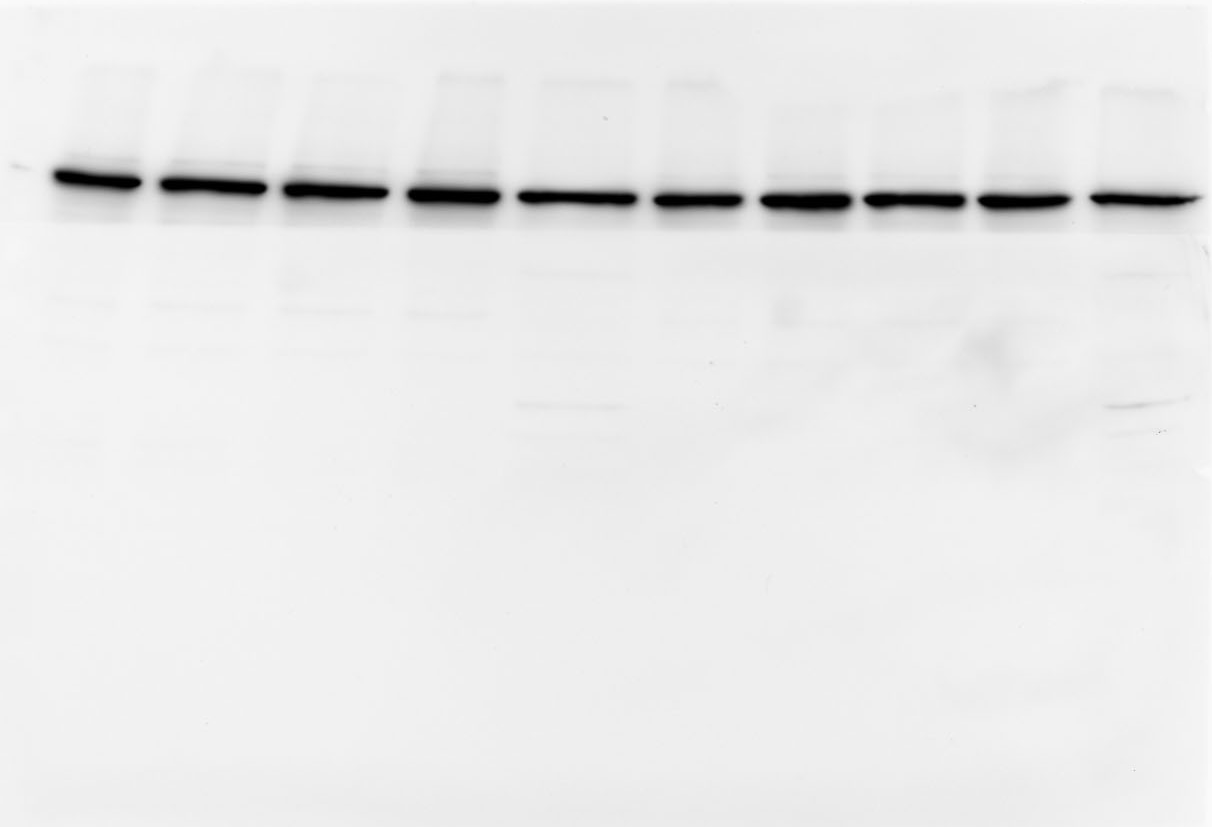

Supplement: Supplementary file 1 [file cancers-12-02188-s001.zip › Figure S4 Western blots/Apoptosis/U937/TIFF/Vinculin.tif]

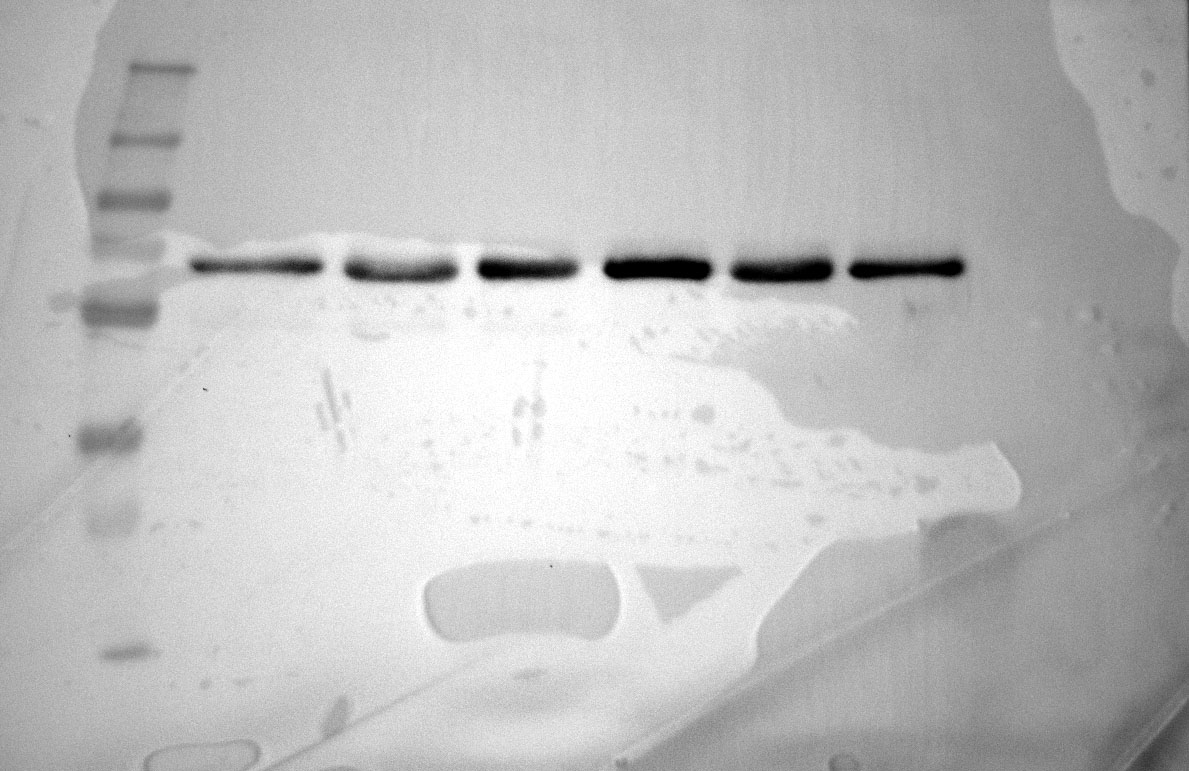

Supplement: Supplementary file 1 [file cancers-12-02188-s001.zip › Figure S4 Western blots/BMDMs/JEPG/p65.jpg]

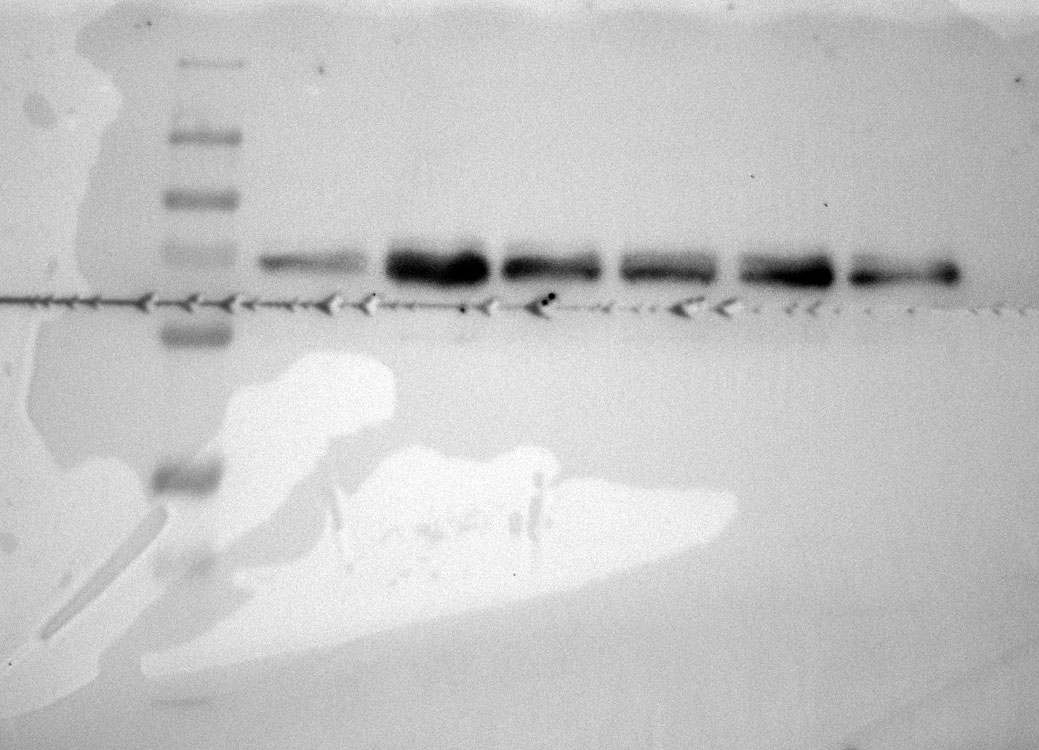

Supplement: Supplementary file 1 [file cancers-12-02188-s001.zip › Figure S4 Western blots/BMDMs/JEPG/pp65.jpg]

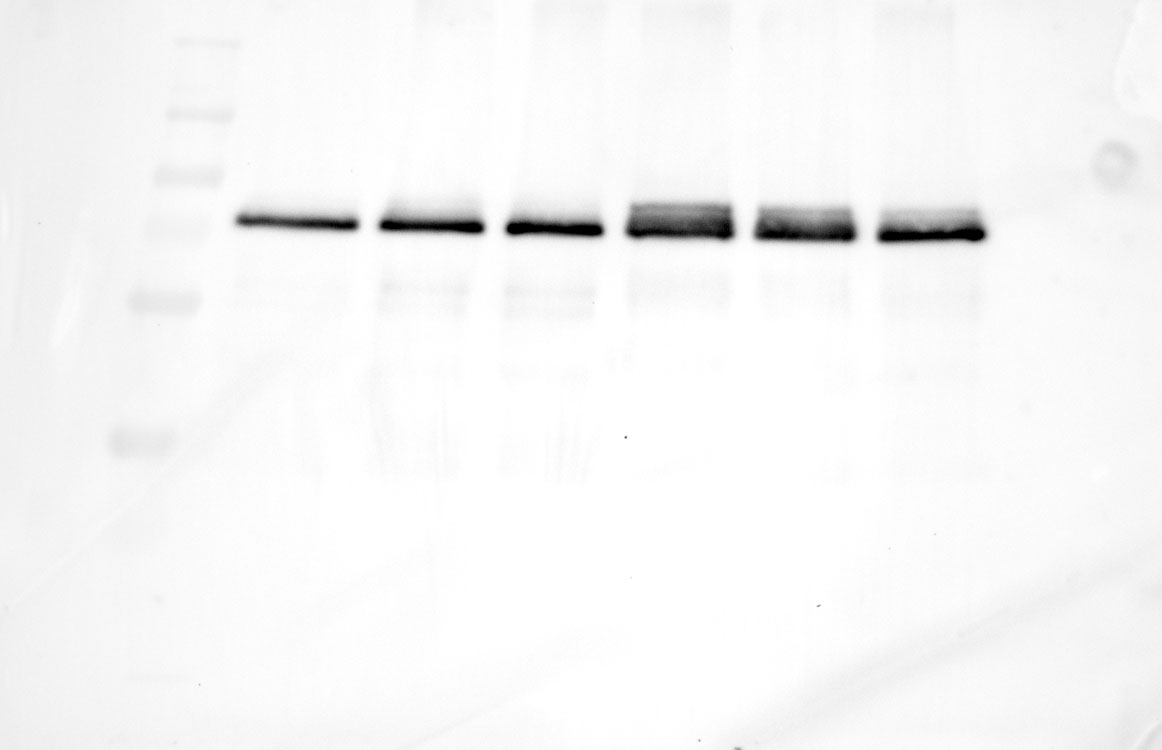

Supplement: Supplementary file 1 [file cancers-12-02188-s001.zip › Figure S4 Western blots/BMDMs/JEPG/RIP1.jpg]

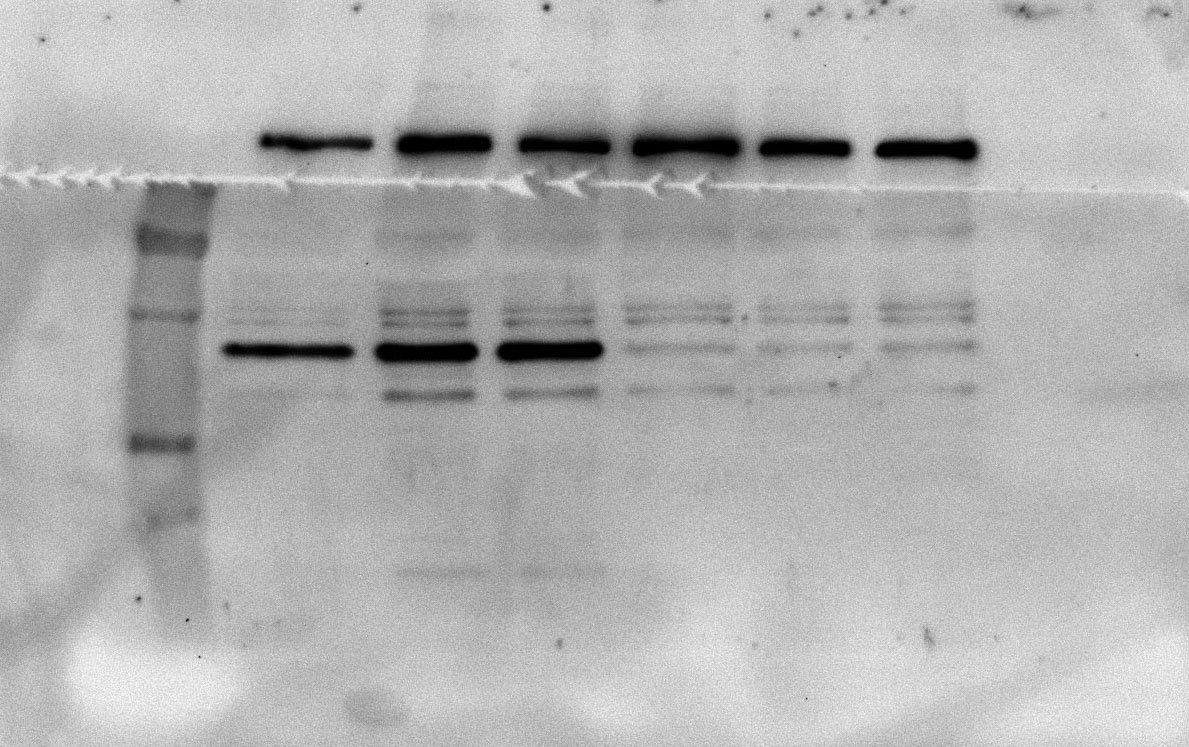

Supplement: Supplementary file 1 [file cancers-12-02188-s001.zip › Figure S4 Western blots/BMDMs/JEPG/Vinculin-YB1.jpg]

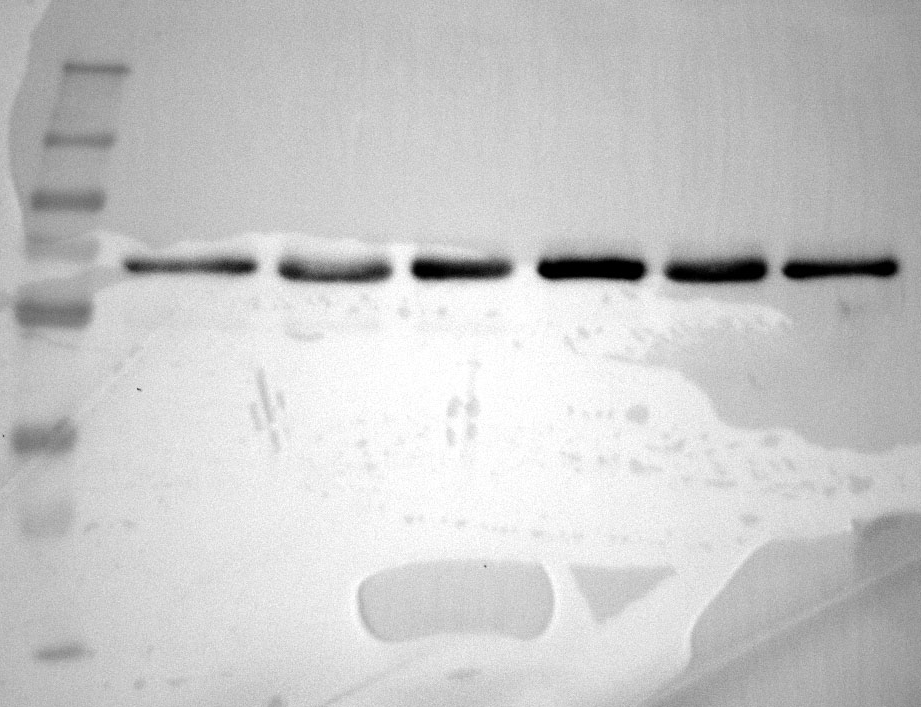

Supplement: Supplementary file 1 [file cancers-12-02188-s001.zip › Figure S4 Western blots/BMDMs/TIFF/p65.tif]

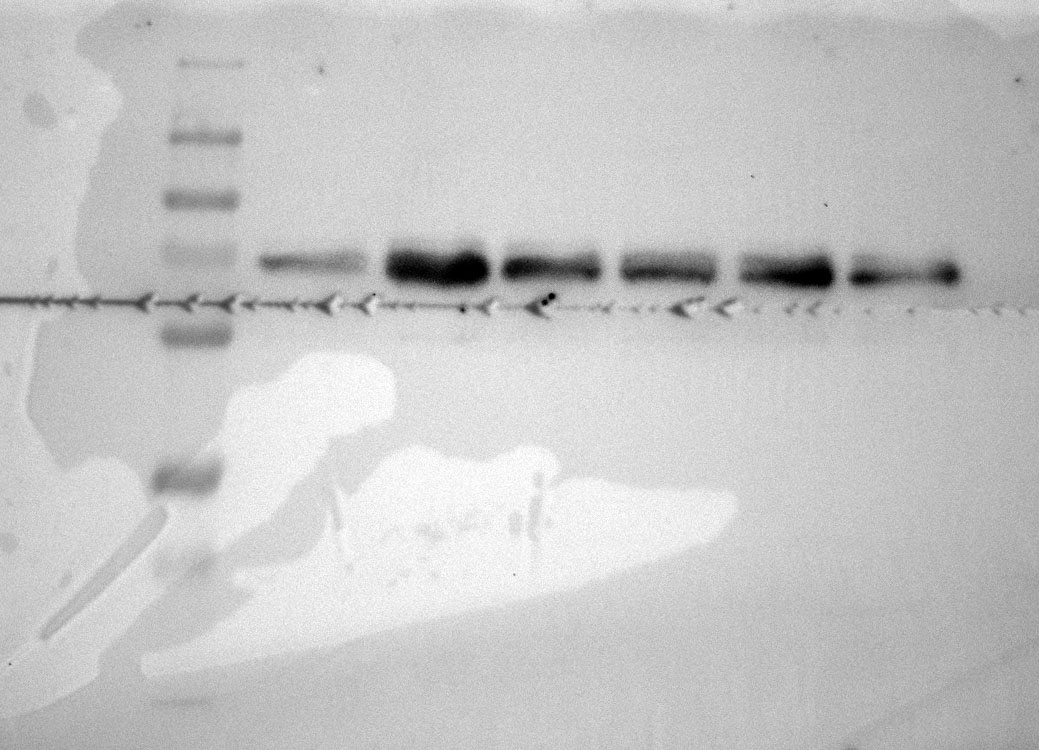

Supplement: Supplementary file 1 [file cancers-12-02188-s001.zip › Figure S4 Western blots/BMDMs/TIFF/pp65.tif]

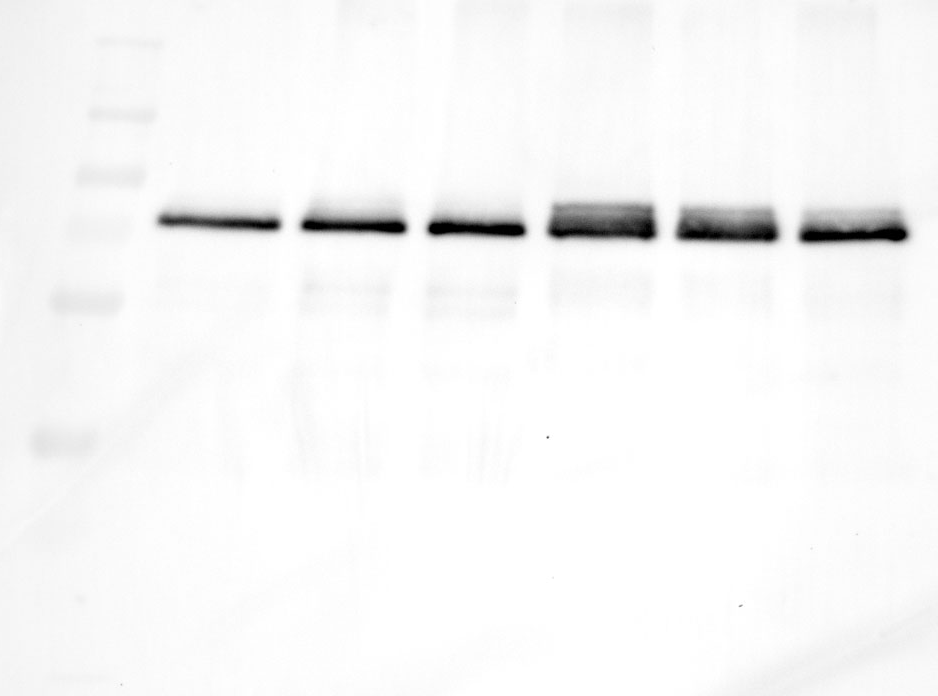

Supplement: Supplementary file 1 [file cancers-12-02188-s001.zip › Figure S4 Western blots/BMDMs/TIFF/RIP1.tif]

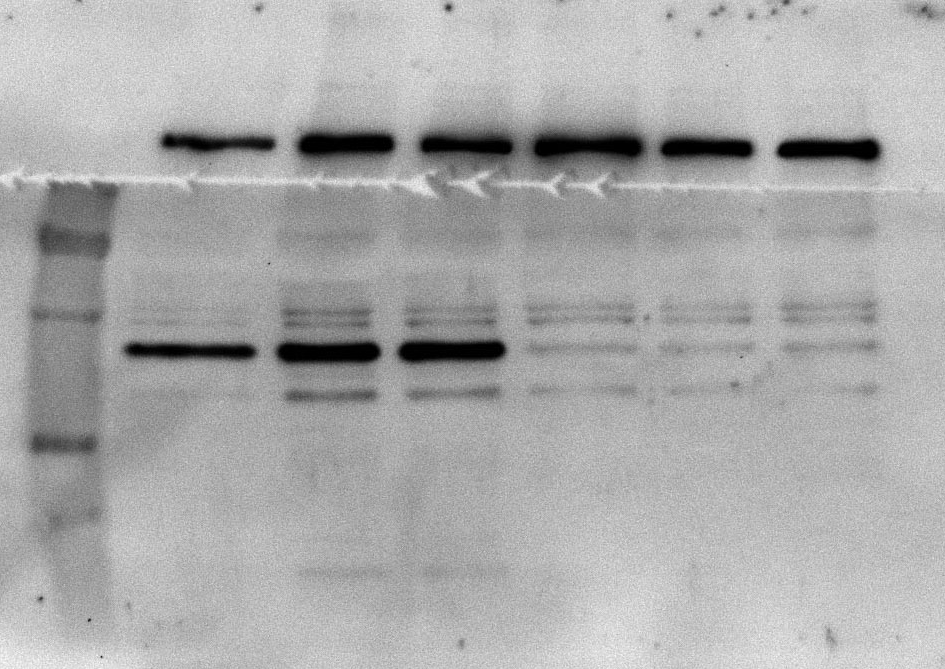

Supplement: Supplementary file 1 [file cancers-12-02188-s001.zip › Figure S4 Western blots/BMDMs/TIFF/Vinculin-YB1.tif]

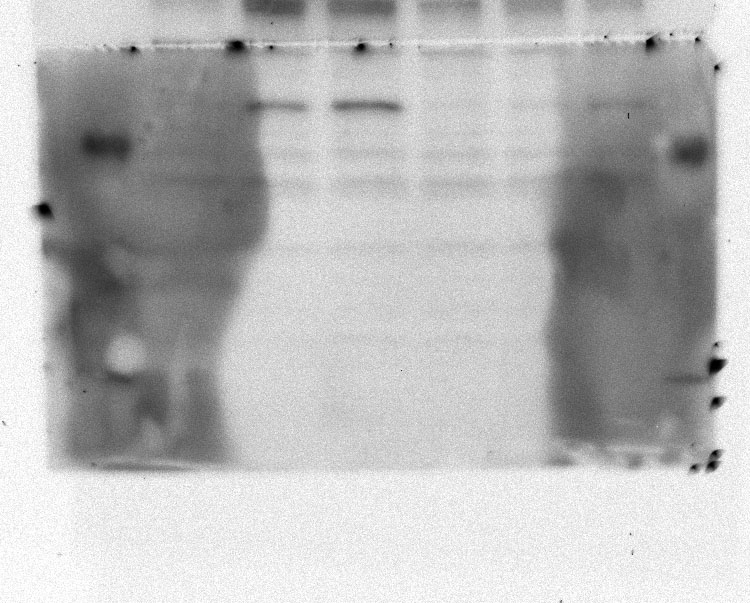

Supplement: Supplementary file 1 [file cancers-12-02188-s001.zip › Figure S4 Western blots/THP1/JEPG/p-Ikba.jpg]

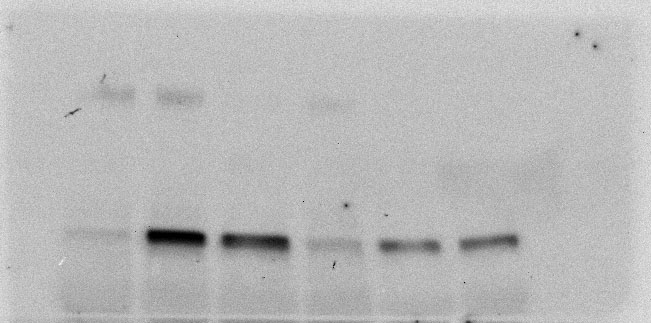

Supplement: Supplementary file 1 [file cancers-12-02188-s001.zip › Figure S4 Western blots/THP1/JEPG/p-IKKa-b.jpg]

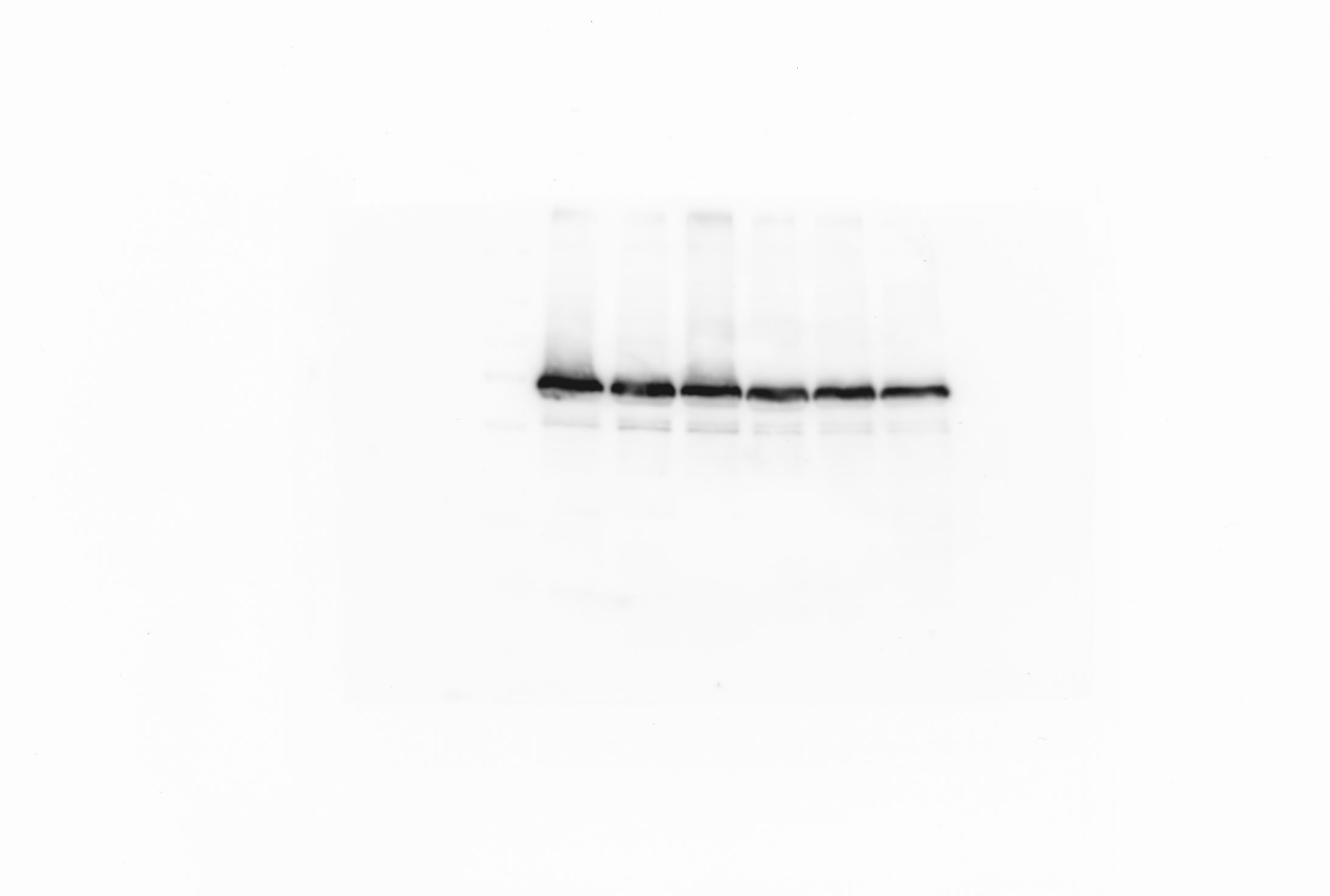

Supplement: Supplementary file 1 [file cancers-12-02188-s001.zip › Figure S4 Western blots/THP1/JEPG/p65.jpg]

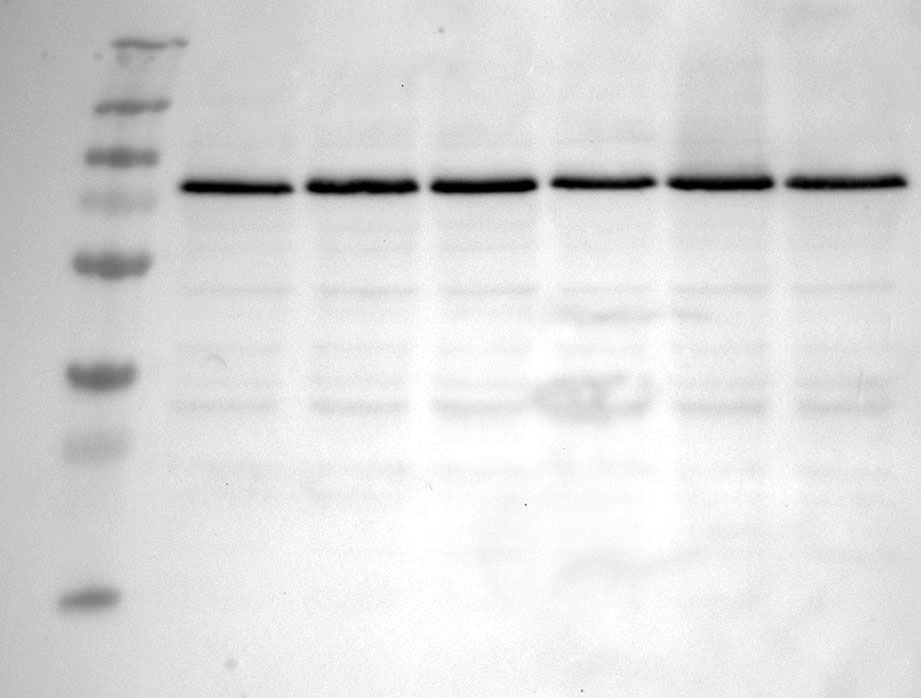

Supplement: Supplementary file 1 [file cancers-12-02188-s001.zip › Figure S4 Western blots/THP1/JEPG/RIPK1.jpg]

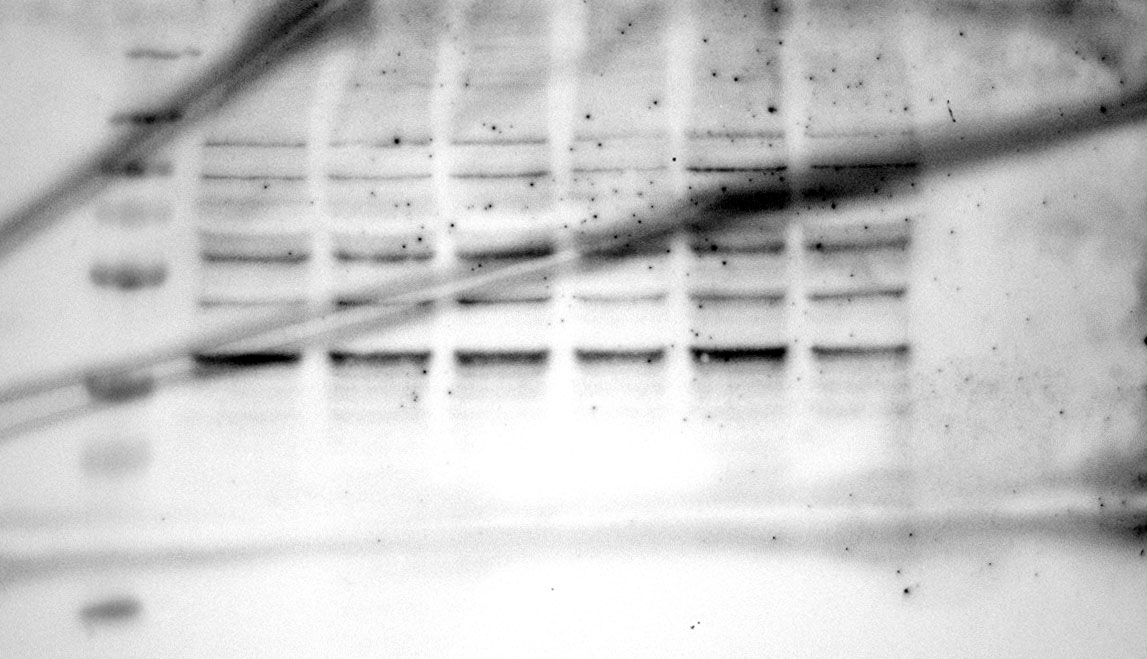

Supplement: Supplementary file 1 [file cancers-12-02188-s001.zip › Figure S4 Western blots/THP1/JEPG/total Ikba.jpg]

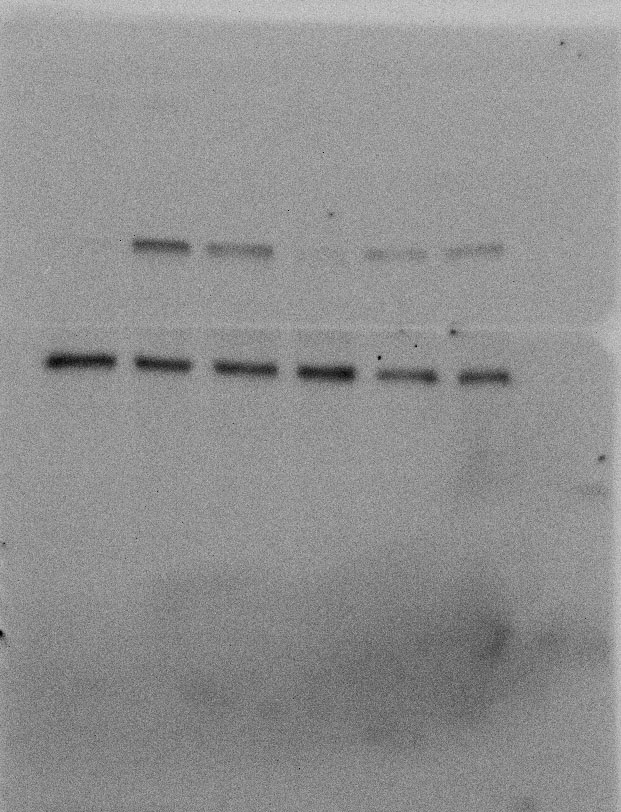

Supplement: Supplementary file 1 [file cancers-12-02188-s001.zip › Figure S4 Western blots/THP1/JEPG/Traf2.jpg]

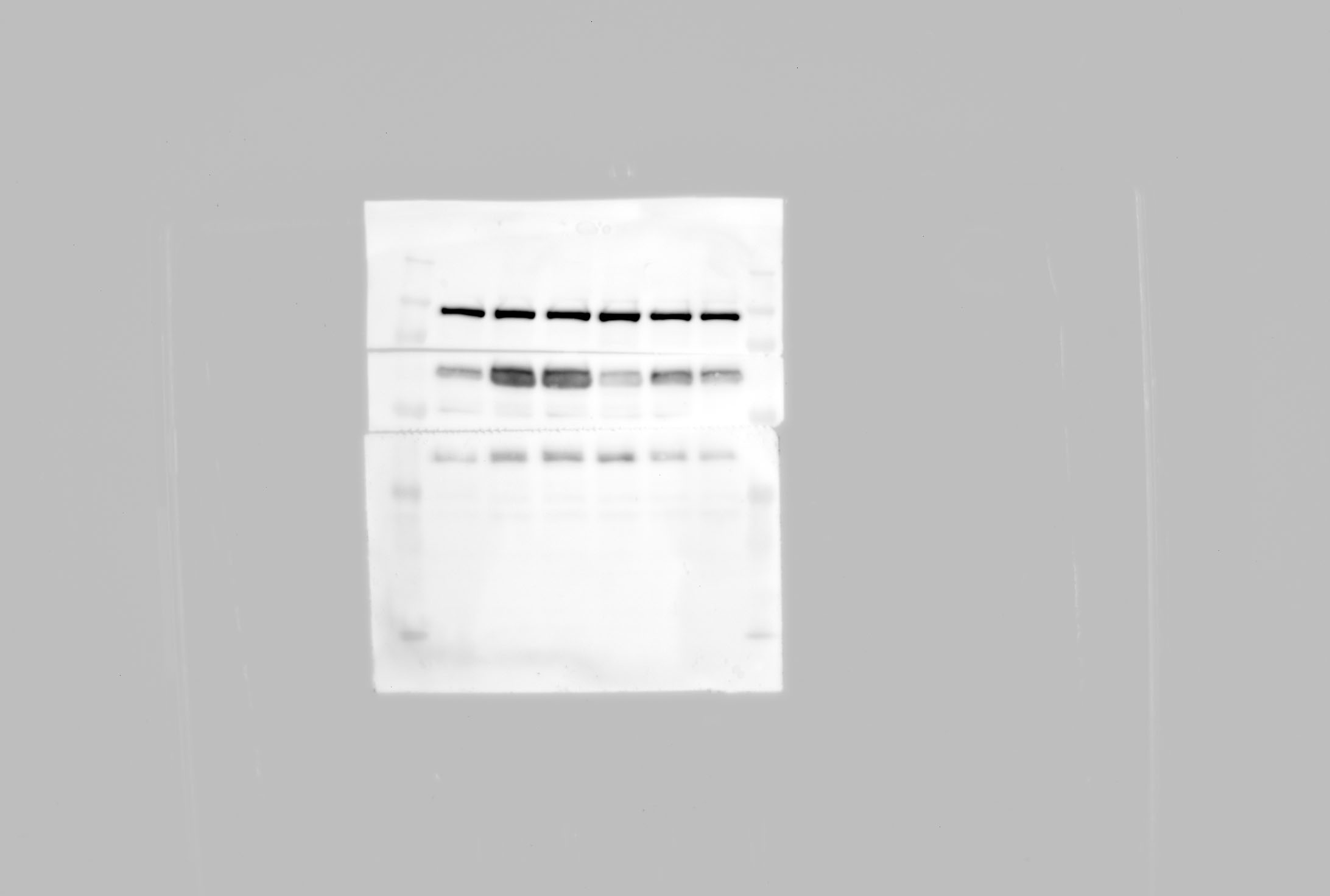

Supplement: Supplementary file 1 [file cancers-12-02188-s001.zip › Figure S4 Western blots/THP1/JEPG/Vinculin-pp65.jpg]

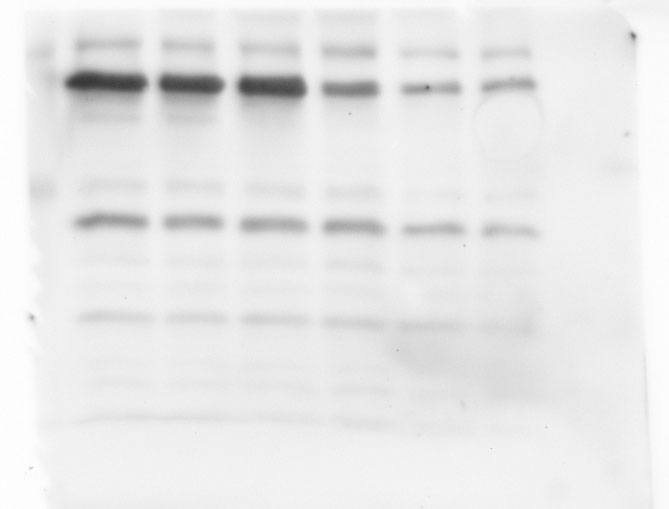

Supplement: Supplementary file 1 [file cancers-12-02188-s001.zip › Figure S4 Western blots/THP1/JEPG/YB1.jpg]

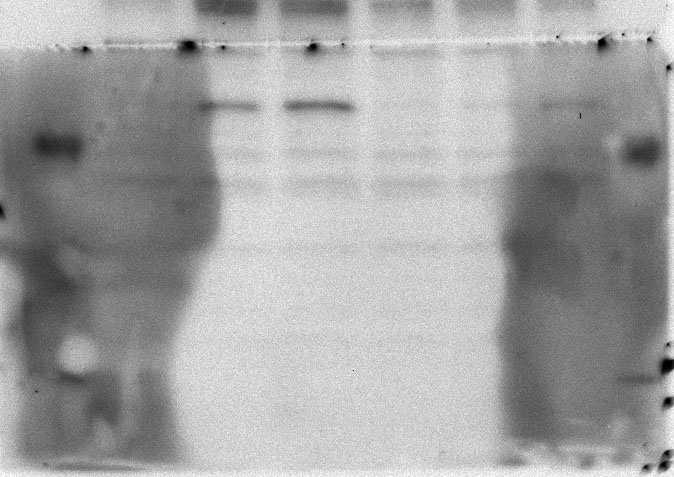

Supplement: Supplementary file 1 [file cancers-12-02188-s001.zip › Figure S4 Western blots/THP1/tiff/p-Ikba.tif]

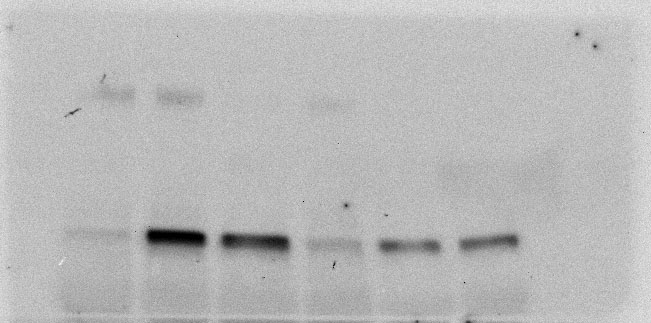

Supplement: Supplementary file 1 [file cancers-12-02188-s001.zip › Figure S4 Western blots/THP1/tiff/p-IKKa-b.tif]

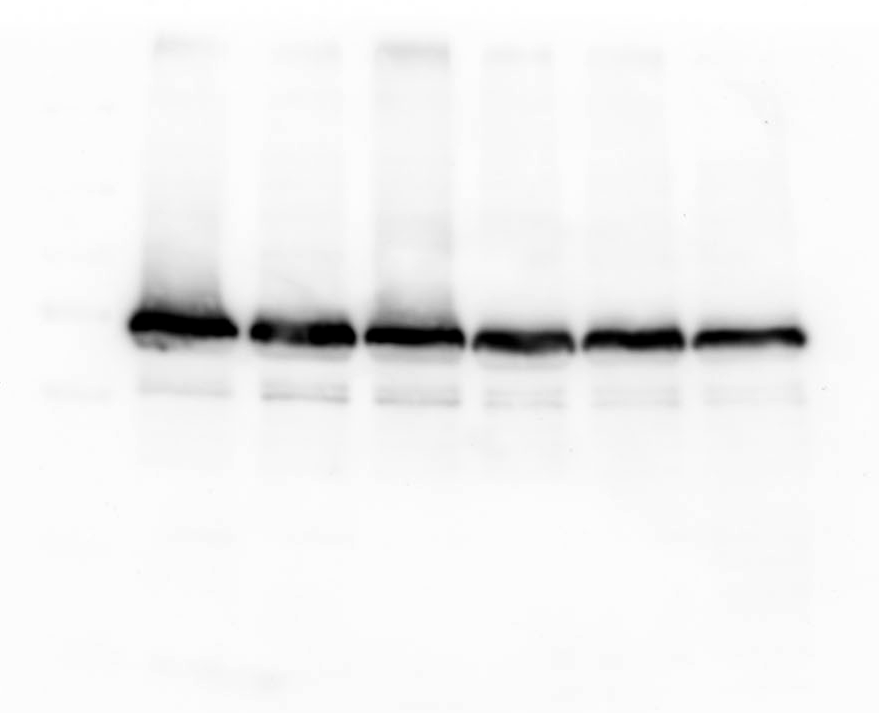

Supplement: Supplementary file 1 [file cancers-12-02188-s001.zip › Figure S4 Western blots/THP1/tiff/p65.tif]

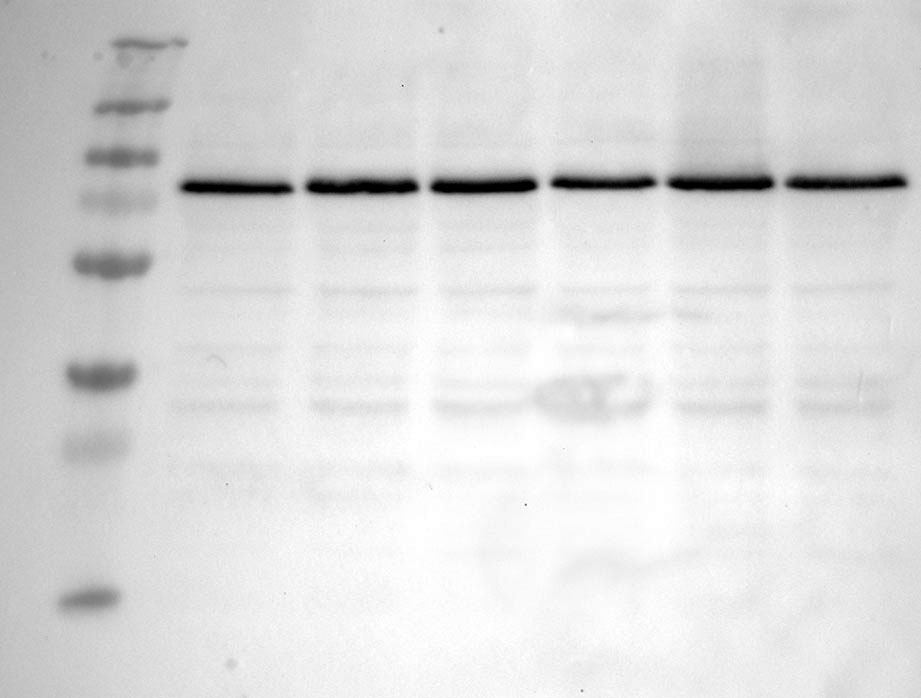

Supplement: Supplementary file 1 [file cancers-12-02188-s001.zip › Figure S4 Western blots/THP1/tiff/RIPK1.tif]

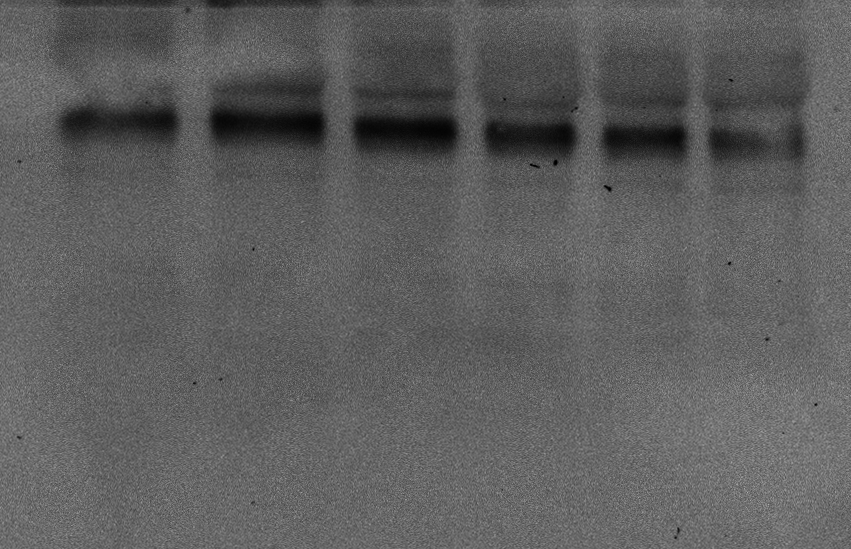

Supplement: Supplementary file 1 [file cancers-12-02188-s001.zip › Figure S4 Western blots/THP1/tiff/TNFR1.tif]

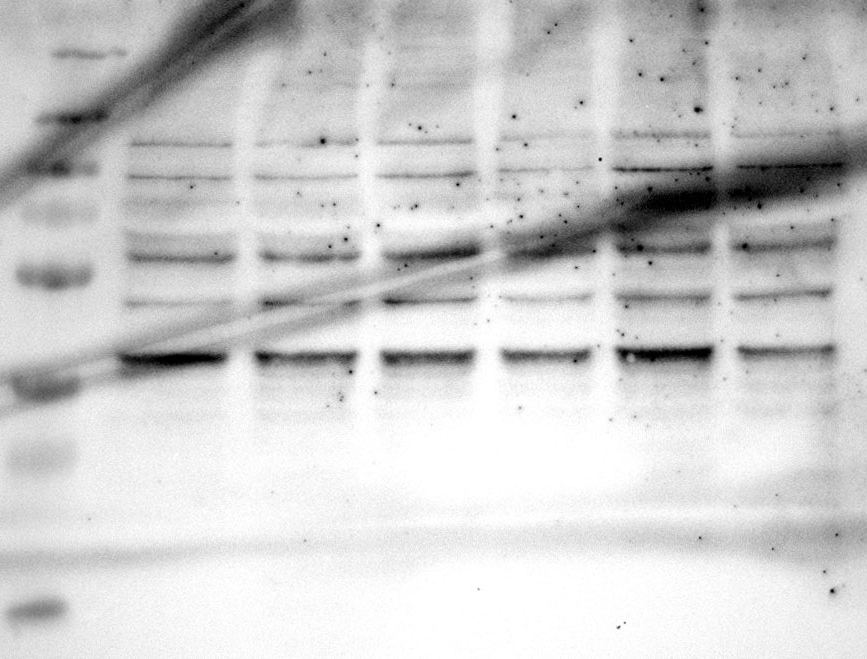

Supplement: Supplementary file 1 [file cancers-12-02188-s001.zip › Figure S4 Western blots/THP1/tiff/total Ikba.tif]

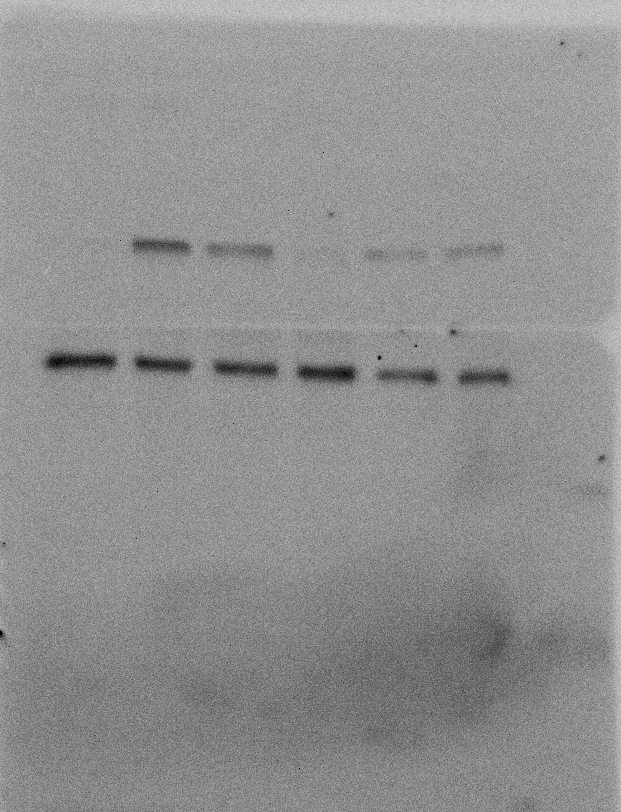

Supplement: Supplementary file 1 [file cancers-12-02188-s001.zip › Figure S4 Western blots/THP1/tiff/Traf2.tif]

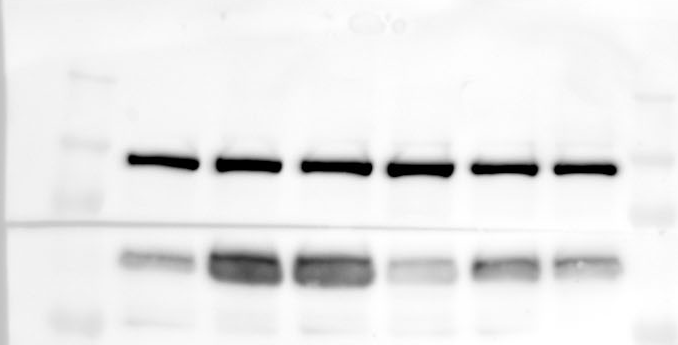

Supplement: Supplementary file 1 [file cancers-12-02188-s001.zip › Figure S4 Western blots/THP1/tiff/Vinculin-pp65.tif]

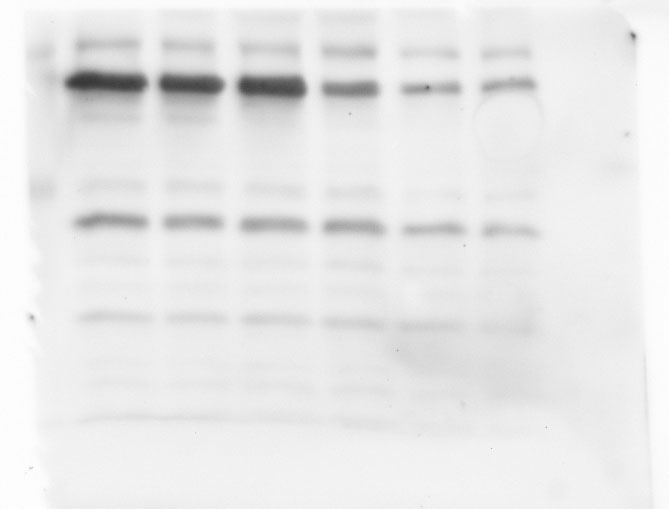

Supplement: Supplementary file 1 [file cancers-12-02188-s001.zip › Figure S4 Western blots/THP1/tiff/YB1.tif]

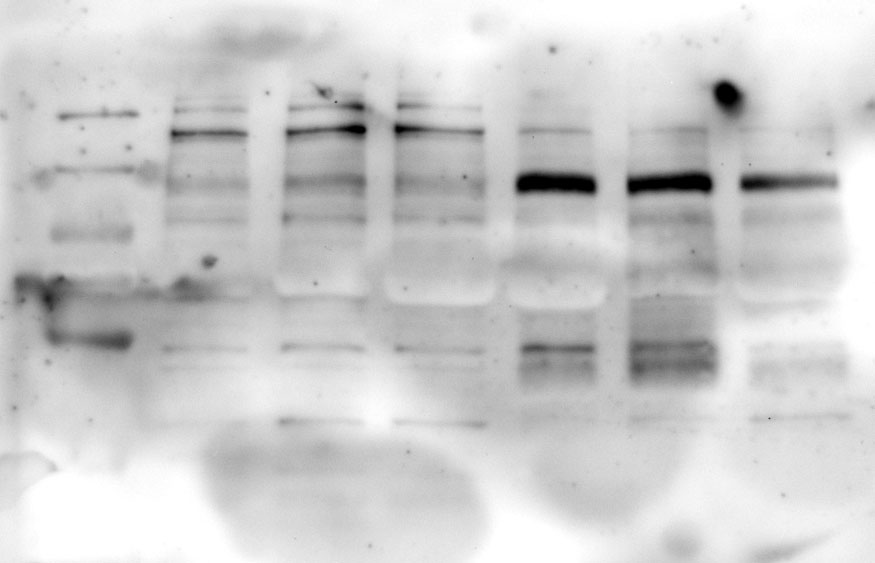

Supplement: Supplementary file 1 [file cancers-12-02188-s001.zip › Figure S4 Western blots/U937/JEPG/p-IkBa.jpg]

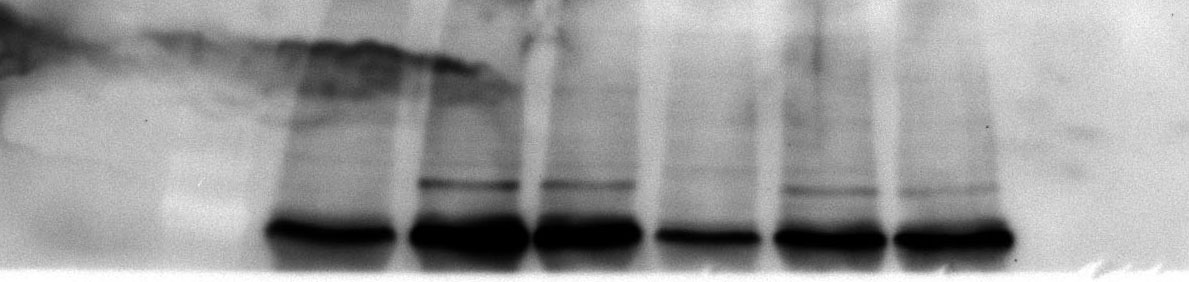

Supplement: Supplementary file 1 [file cancers-12-02188-s001.zip › Figure S4 Western blots/U937/JEPG/p-Ikkb-a.jpg]

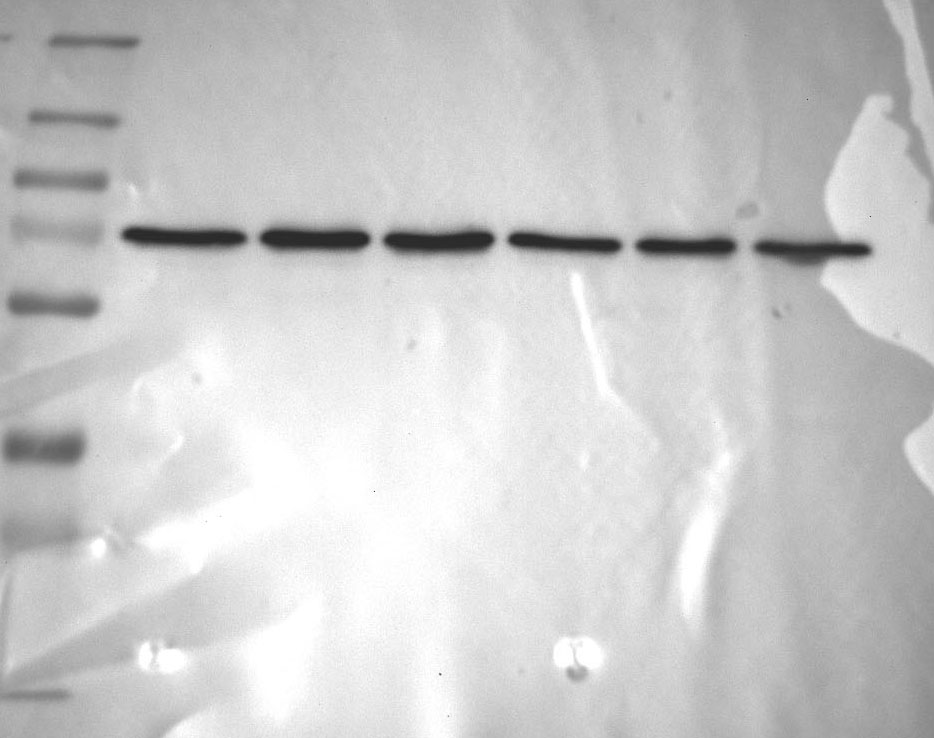

Supplement: Supplementary file 1 [file cancers-12-02188-s001.zip › Figure S4 Western blots/U937/JEPG/p65.jpg]

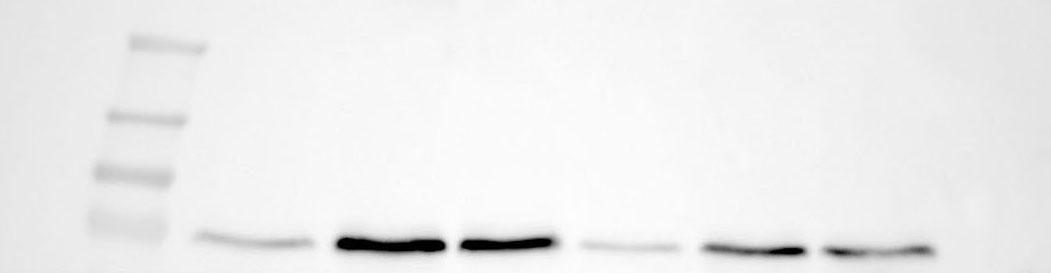

Supplement: Supplementary file 1 [file cancers-12-02188-s001.zip › Figure S4 Western blots/U937/JEPG/pp65.jpg]

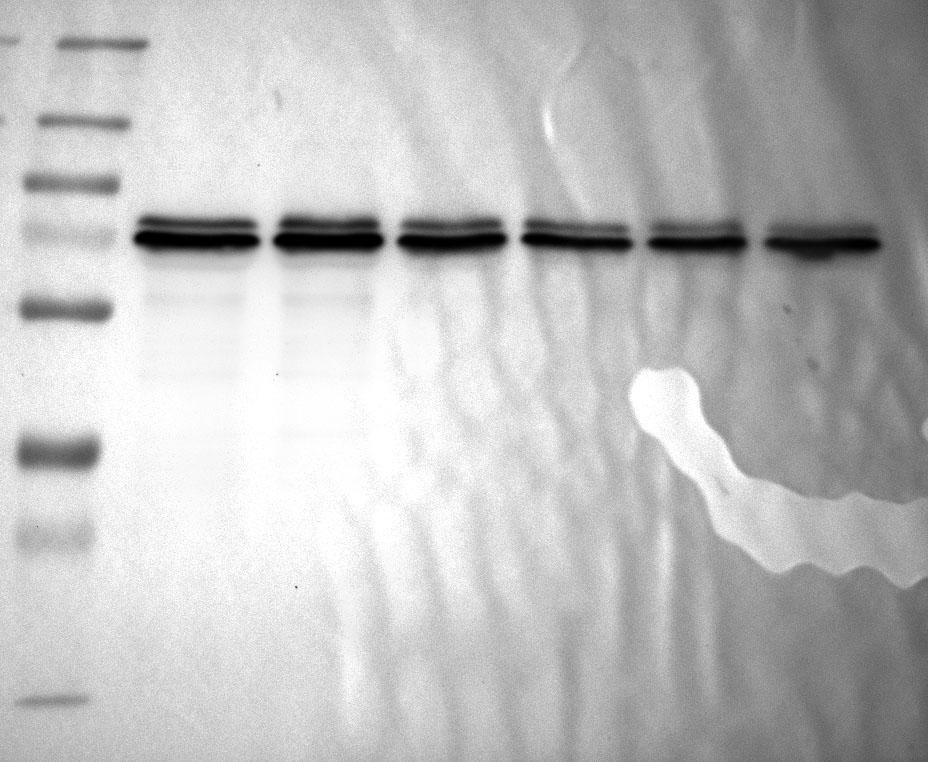

Supplement: Supplementary file 1 [file cancers-12-02188-s001.zip › Figure S4 Western blots/U937/JEPG/RIP1.jpg]

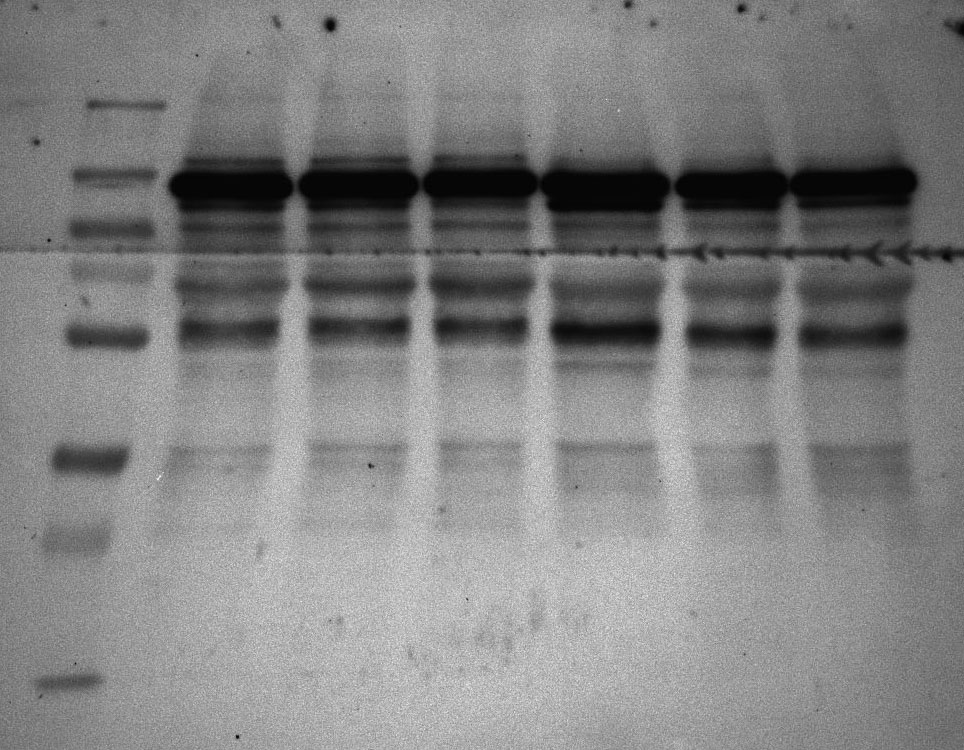

Supplement: Supplementary file 1 [file cancers-12-02188-s001.zip › Figure S4 Western blots/U937/JEPG/TNFR1.jpg]

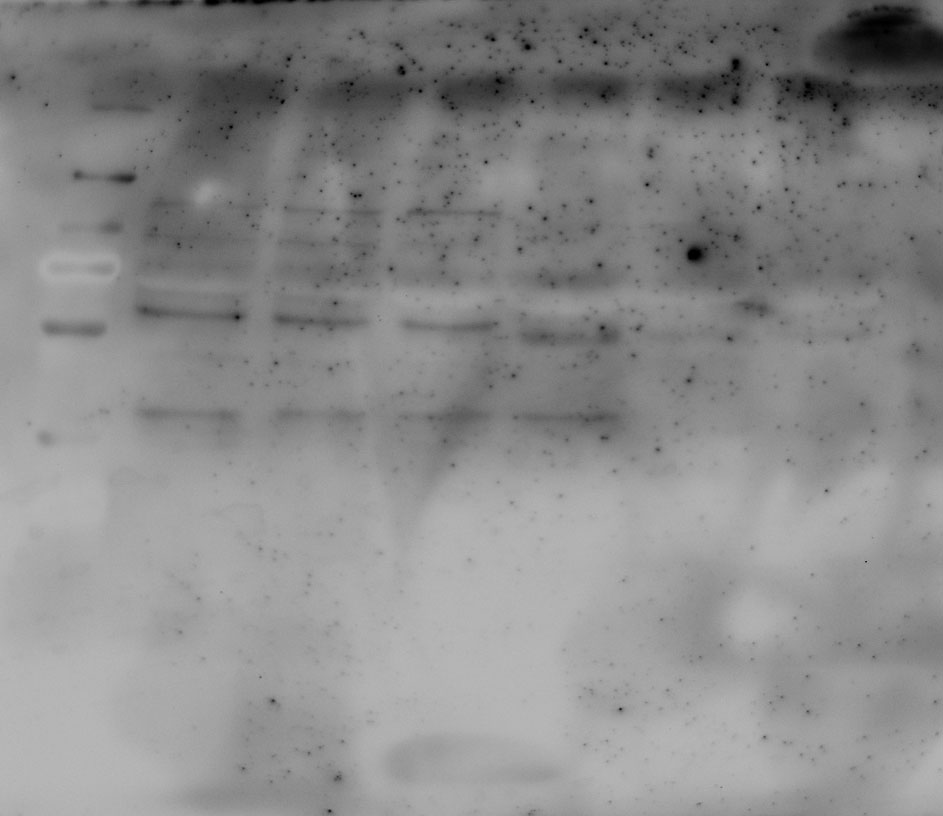

Supplement: Supplementary file 1 [file cancers-12-02188-s001.zip › Figure S4 Western blots/U937/JEPG/Total IkBa.jpg]

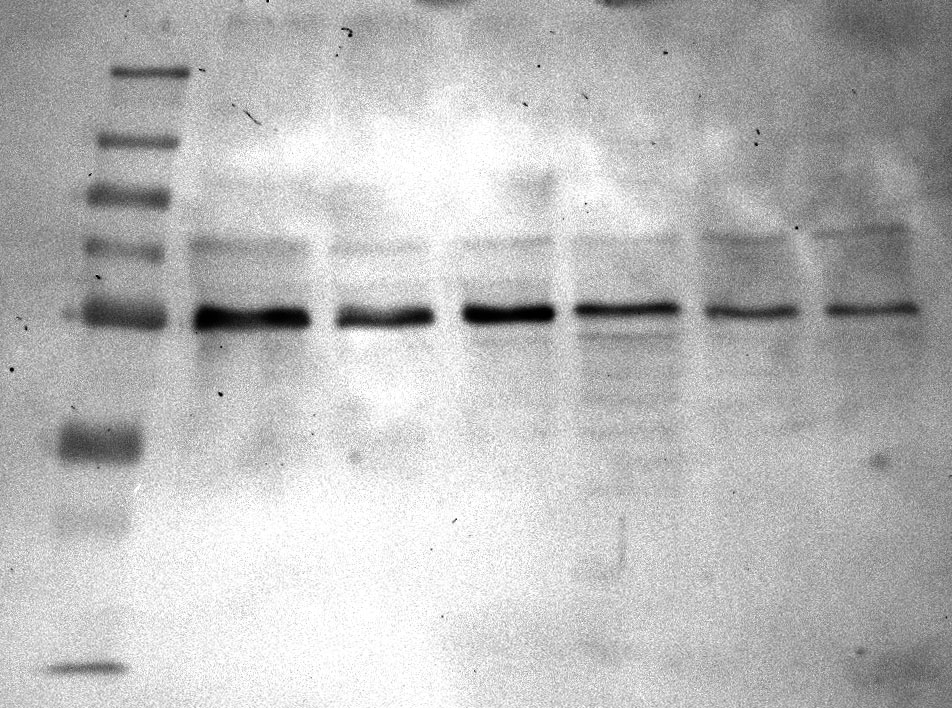

Supplement: Supplementary file 1 [file cancers-12-02188-s001.zip › Figure S4 Western blots/U937/JEPG/TRAF2.jpg]

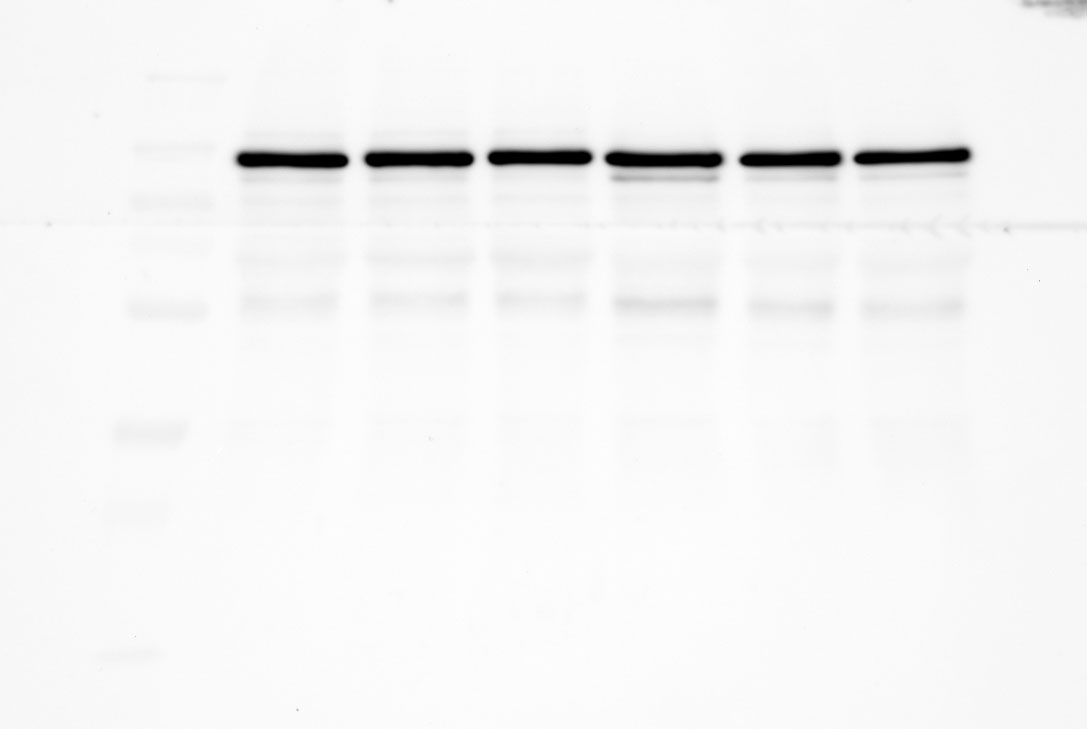

Supplement: Supplementary file 1 [file cancers-12-02188-s001.zip › Figure S4 Western blots/U937/JEPG/Vinculin.jpg]

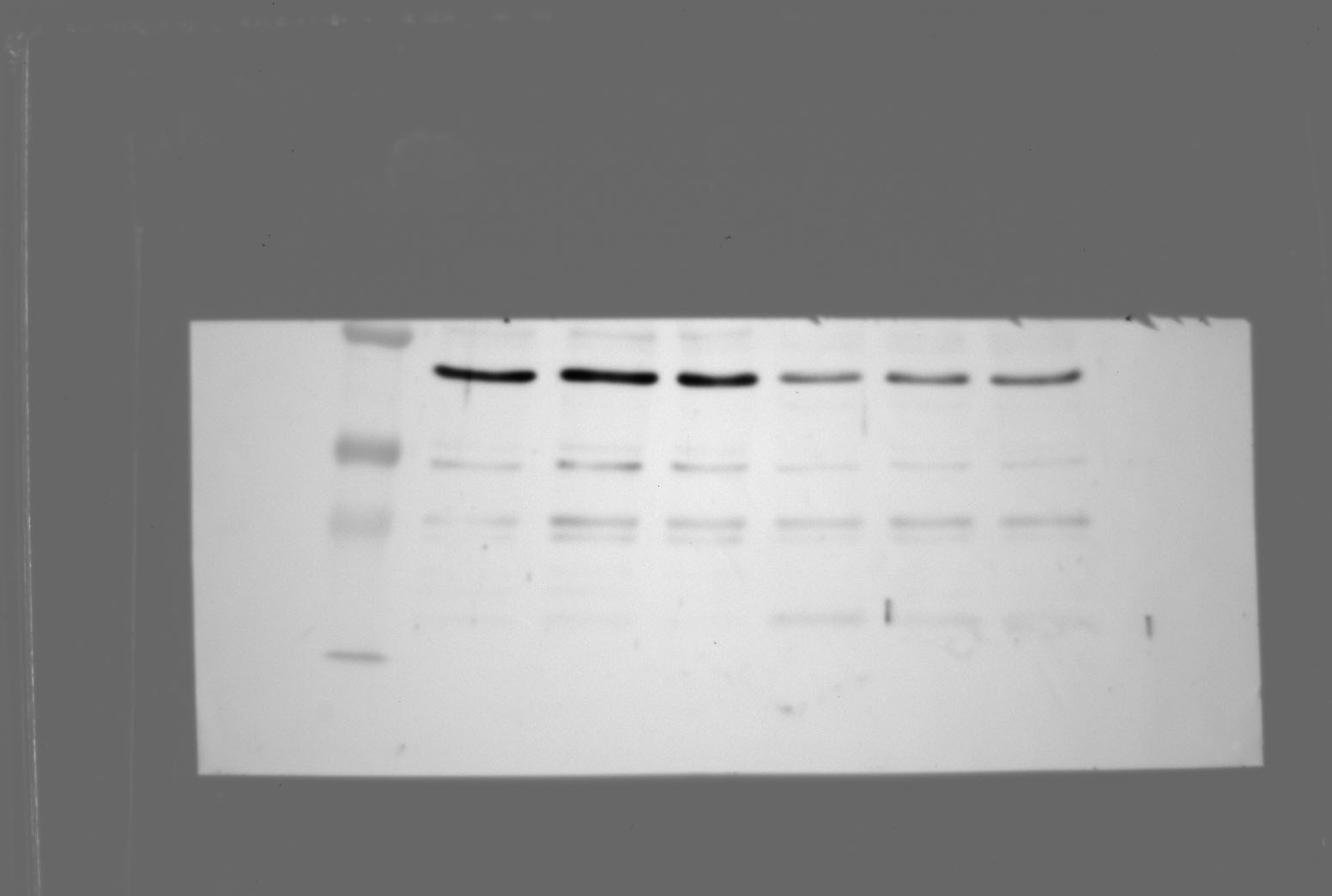

Supplement: Supplementary file 1 [file cancers-12-02188-s001.zip › Figure S4 Western blots/U937/JEPG/YB1.jpg]

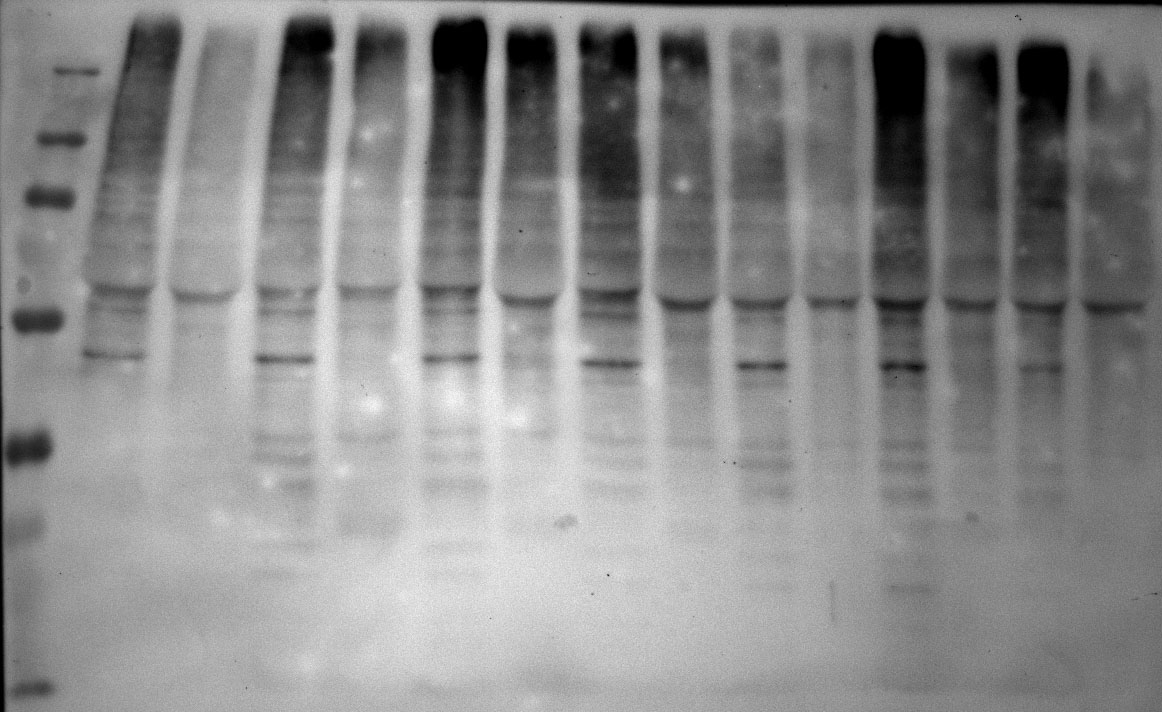

Supplement: Supplementary file 1 [file cancers-12-02188-s001.zip › Figure S4 Western blots/U937/TIFF/FK2.tif]

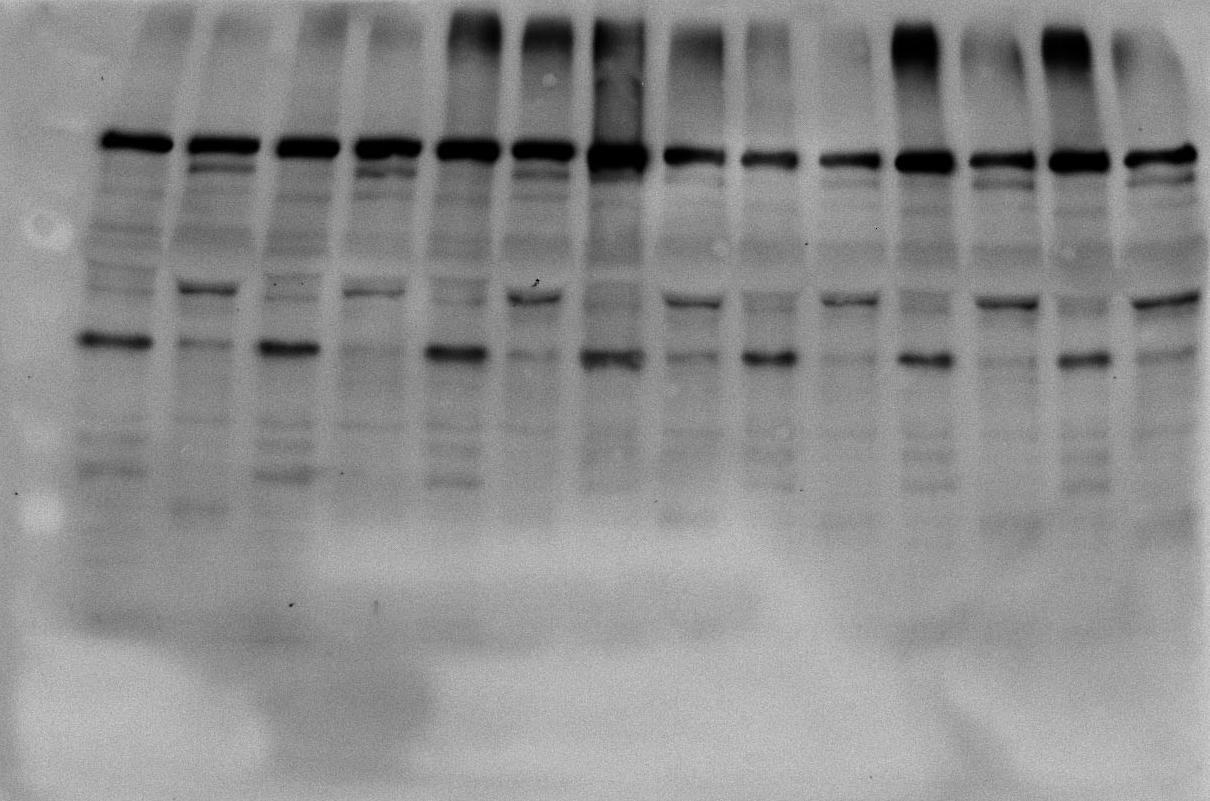

Supplement: Supplementary file 1 [file cancers-12-02188-s001.zip › Figure S4 Western blots/U937/TIFF/Mg132 YB1-Vinculin.tif]

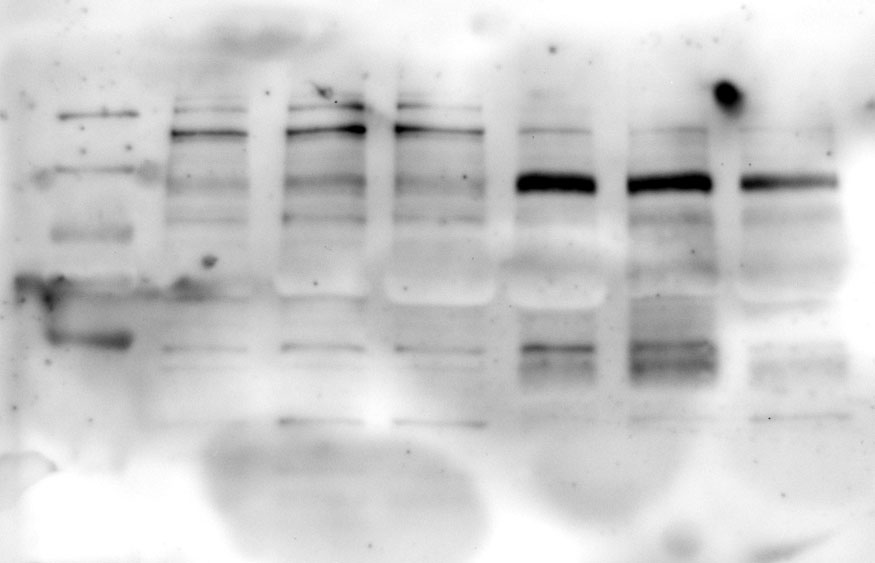

Supplement: Supplementary file 1 [file cancers-12-02188-s001.zip › Figure S4 Western blots/U937/TIFF/p-IkBa.tif]

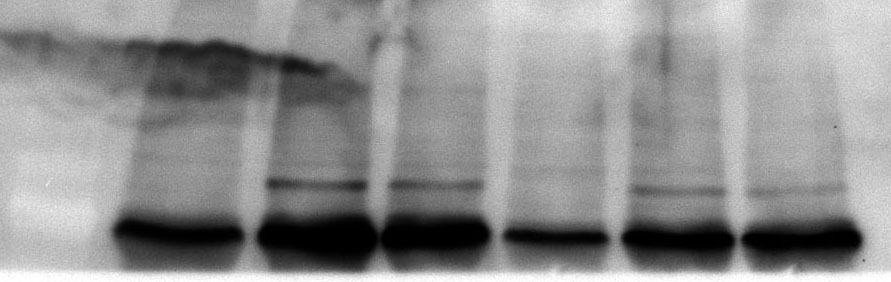

Supplement: Supplementary file 1 [file cancers-12-02188-s001.zip › Figure S4 Western blots/U937/TIFF/p-Ikkb-a.tif]

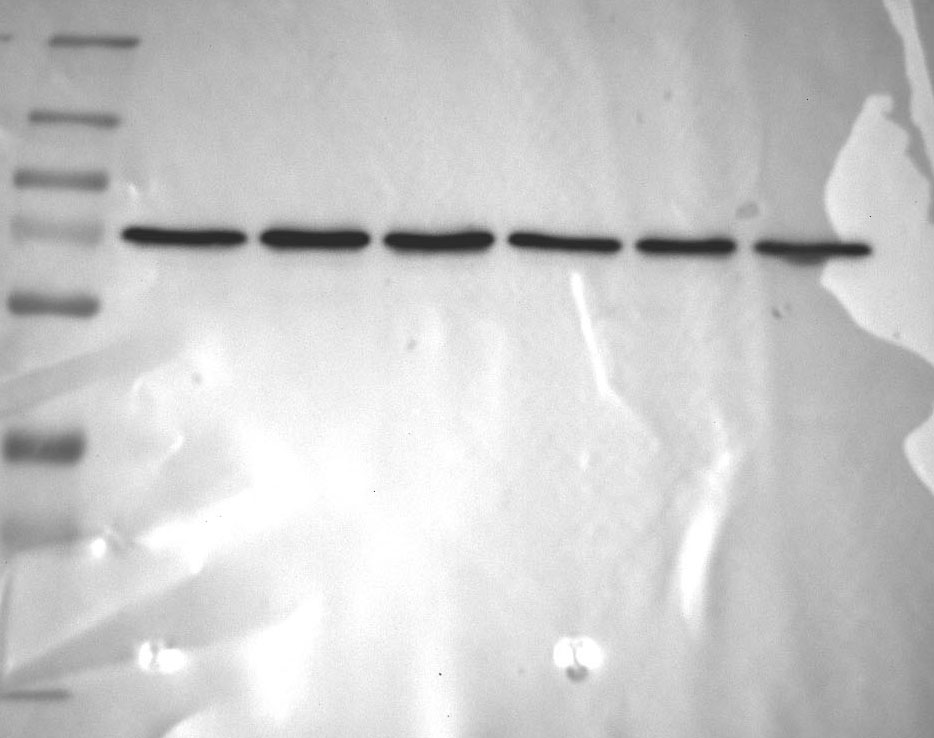

Supplement: Supplementary file 1 [file cancers-12-02188-s001.zip › Figure S4 Western blots/U937/TIFF/p65.tif]

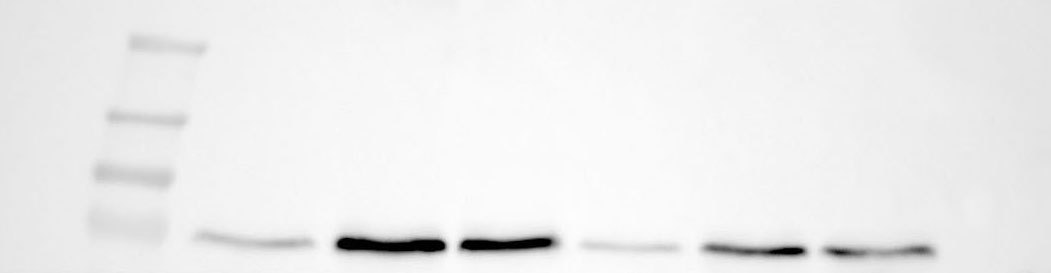

Supplement: Supplementary file 1 [file cancers-12-02188-s001.zip › Figure S4 Western blots/U937/TIFF/pp65.tif]

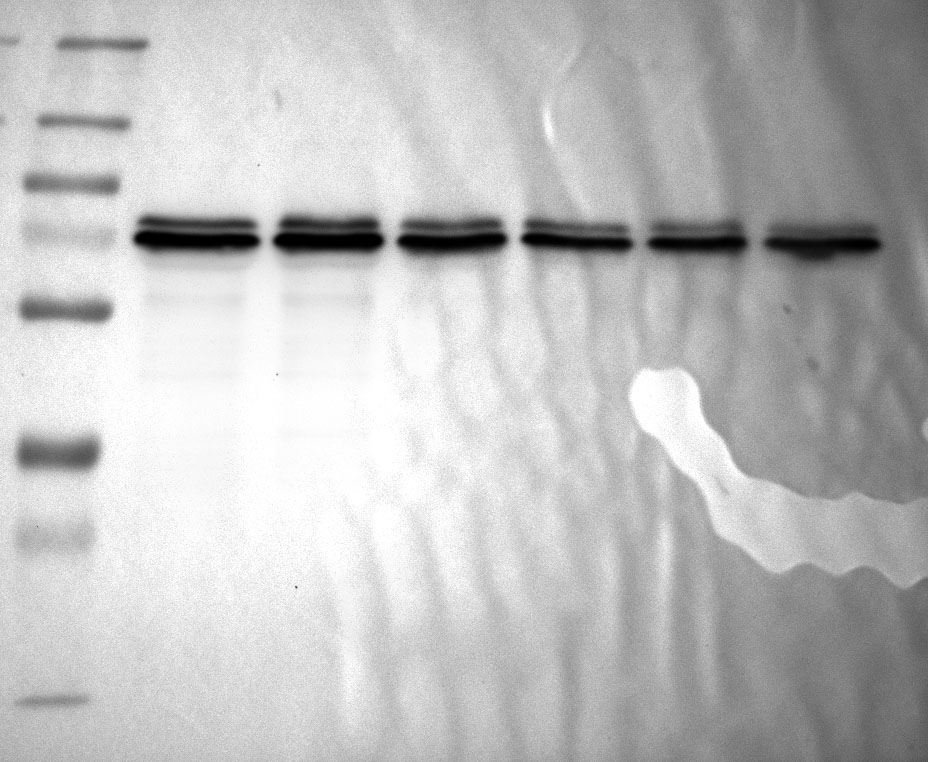

Supplement: Supplementary file 1 [file cancers-12-02188-s001.zip › Figure S4 Western blots/U937/TIFF/RIP1.tif]

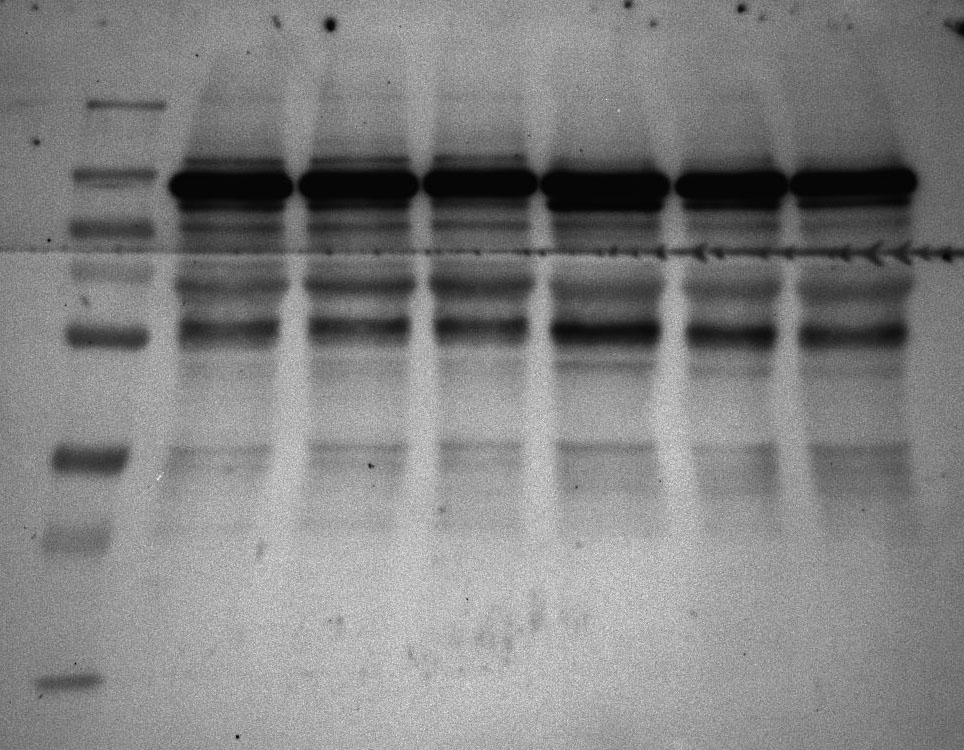

Supplement: Supplementary file 1 [file cancers-12-02188-s001.zip › Figure S4 Western blots/U937/TIFF/TNFR1.tif]

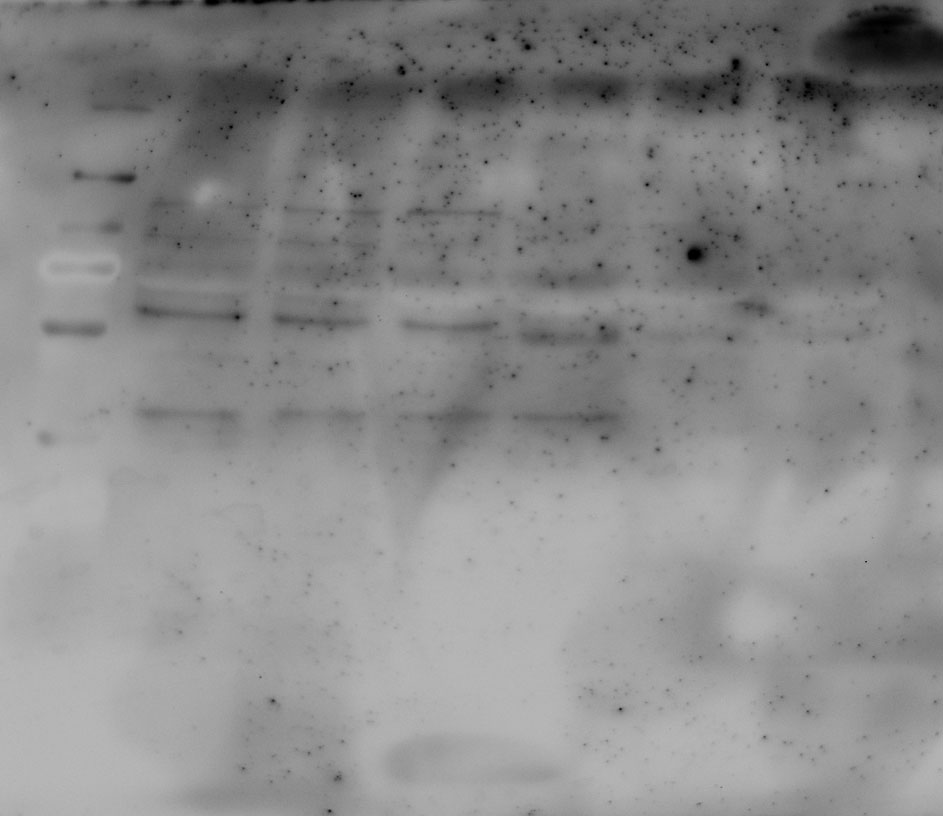

Supplement: Supplementary file 1 [file cancers-12-02188-s001.zip › Figure S4 Western blots/U937/TIFF/Total IkBa.tif]

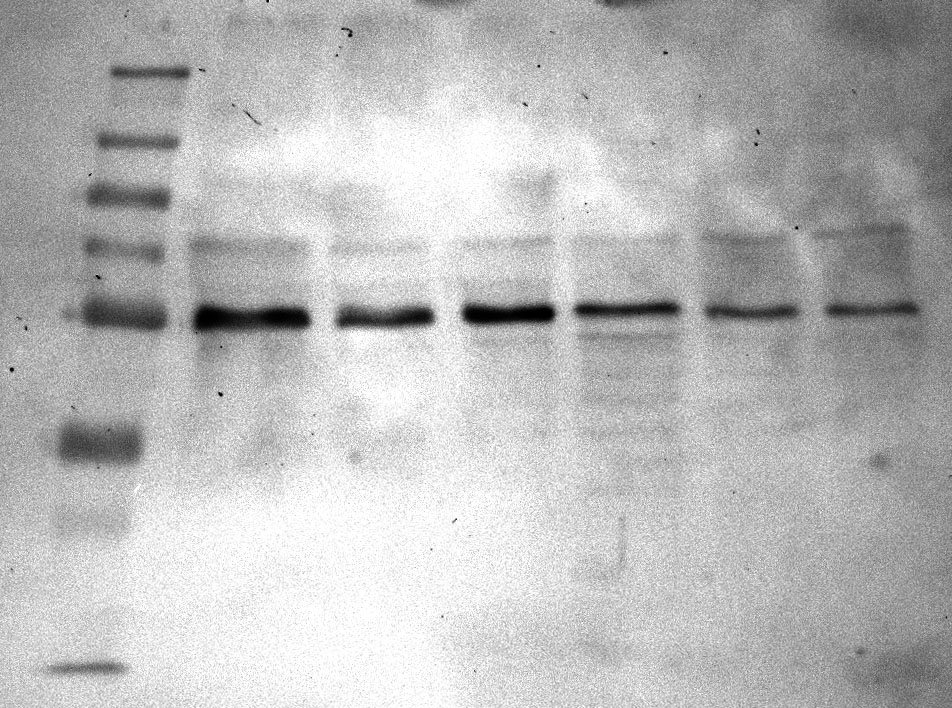

Supplement: Supplementary file 1 [file cancers-12-02188-s001.zip › Figure S4 Western blots/U937/TIFF/TRAF2.tif]

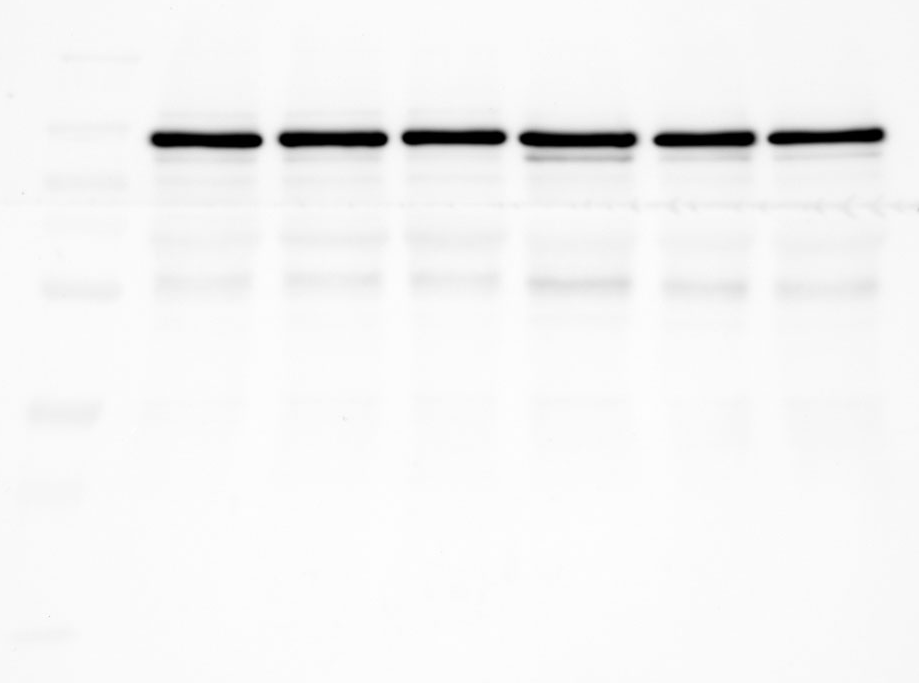

Supplement: Supplementary file 1 [file cancers-12-02188-s001.zip › Figure S4 Western blots/U937/TIFF/Vinculin.tif]

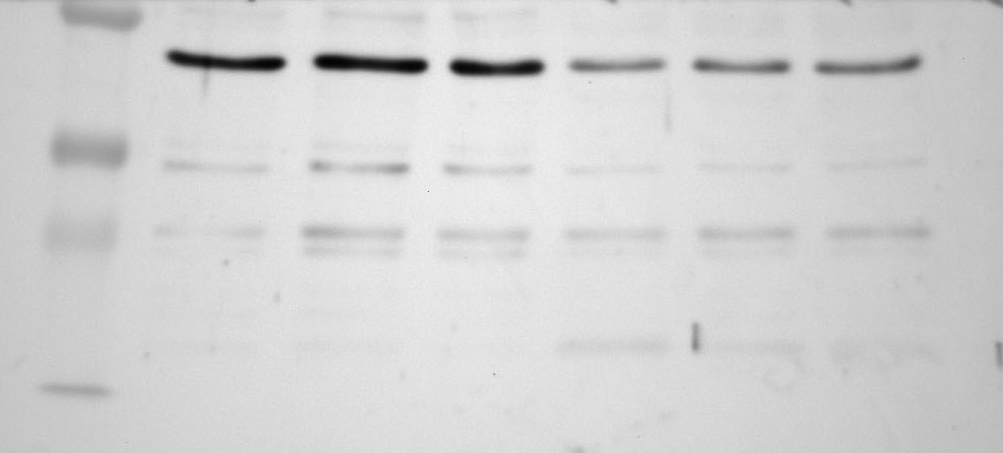

Supplement: Supplementary file 1 [file cancers-12-02188-s001.zip › Figure S4 Western blots/U937/TIFF/YB1.tif]
